# New Tradeoffs for Decremental Approximate All-Pairs Shortest Paths

**DOI:** 10.1007/s00453-026-01407-2

**Published:** 2026-09-28

**Authors:** Michal Dory, Sebastian Forster, Yasamin Nazari, Tijn de Vos

**Affiliations:** 1 University of Haifa, Haifa, Israel; 2 Department of Computer Science, University of Salzburg, Salzburg, Austria; 3 VU Amsterdam, Amsterdam, Netherlands; 4 TU Graz, Graz, Austria

**Keywords:** All-pairs shortest paths, Distance oracle, Decremental graph algorithm, Approximate shortest paths

## Abstract

We provide new tradeoffs between approximation and running time for the decremental all-pairs shortest paths (APSP) problem. For undirected graphs with *m* edges and *n* nodes undergoing edge deletions, we provide four new approximate decremental APSP algorithms, two for weighted and two for unweighted graphs. Our first result is (2+ϵ)-APSP with total update time $\tilde{O}(m^{1/2}n^{3/2})$ (when $m= n^{1+c}$ for any constant 0<c<1). Our second result is $(2+\epsilon , W_{u,v})$-APSP with total update time $\tilde{O}(nm^{3/4})$, where the second term is an additive stretch with respect to $W_{u,v}$, the maximum weight on the current shortest path from *u* to *v*. Prior to our work the fastest algorithm for weighted graphs with approximation at most 3 had total $\tilde{O}(mn)$ update time for (1+ϵ)-APSP (Bernstein [11], SICOMP 2016). Our third result is (2+ϵ)-APSP for unweighted graphs in $\tilde{O}(m^{7/4})$ update time, which for sparse graphs ($m=o(n^{8/7})$) is the first subquadratic (2+ϵ)-approximation. Our last result for unweighted graphs is (1+ϵ,2(k-1))-APSP, for k≥2, with $\tilde{O}(n^{2-1/k}m^{1/k})$ total update time. For comparison, in the special case of (1+ϵ,2)-approximation, this improves over the state-of-the-art algorithm by Henzinger et al. [2], SICOMP 2016 with total update time of $\tilde{O}(n^{2.5})$. All of our results are randomized, work against an oblivious adversary, and have constant query time.

## Introduction

The dynamic algorithms paradigm is becoming increasingly popular for studying algorithmic questions in the presence of gradually changing inputs. A natural goal in this area is to design algorithms that process each change to the input as fast as possible to adapt the algorithm’s output (or a data structure for querying the output) to the current state of the input. The time spent after an update to perform these computations is called the *update time* of the algorithm. In many cases, bounds on the update time are obtained in an *amortized* sense as an average over a long enough sequence of updates. Among dynamic graph problems, the question of maintaining exact or approximate shortest paths has received considerable attention in the past two decades [1–22]. The main focus usually lies on maintaining a distance oracle that answers queries for the distance between a pair of nodes. For this problem, we call an algorithm *fully dynamic* if it supports both insertions and deletions of edges, and *partially dynamic* if it supports only one type of updates; in particular we call it *decremental* if it only supports edge deletions (which is the focus of this paper), and *incremental* if it only supports edge insertions.

The running times of partially dynamic algorithms are usually characterized by their bounds on the *total update time*, which is the accumulated time for processing all updates in a sequence of at most *m* deletions (where *m* is the maximum number of edges ever contained the graph). A typical design choice, which we also impose in this paper, is small (say polylogarithmic) query time. In particular, our algorithms will have constant query time. While fully dynamic algorithms are more general, the restriction to only one type of updates in partially dynamic algorithms often admits much faster update times. In particular, some partially dynamic algorithms have a total update time that almost matches the running time of the fastest static algorithm, i.e., computing *all* updates does not take significantly more time than processing the graph once.

**Decremental shortest paths.** For the decremental single-source shortest paths problem, conditional lower bounds [10, 23] suggest that *exact* decremental algorithms have an Ω(mn) bottleneck in their total update time (up to subpolynomial factors). On the other hand, this problem admits a (1+ϵ)-approximation (also called *stretch*) with total update time $m^{1 + o(1)}$ in weighted, undirected graphs [1, 6, 8, 14, 18], which exceeds the running time of the state-of-the art static algorithm by only a subpolynomial factor. Hence for the single-source shortest paths problem on undirected graphs we can fully characterize for which multiplicative stretches the total update time of the fastest decremental algorithm matches (up to subpolynomial factors) the running time of the fastest static algorithm.

Obtaining a similar characterization for the decremental all-pairs shortest paths (APSP) problem is an intriguing open question. Conditional lower bounds [10, 24] suggest an $\Omega ({mn})$ bottleneck in the total update time of decremental APSP algorithms with (a) any finite stretch on *directed* graphs and (b) with any stretch guarantee with a multiplicative term of $\alpha \ge 1$ and an additive term of $\beta \ge 0$ with $2 \alpha + \beta < 4$ on *undirected* graphs. This motivates the study of decremental $(\alpha , \beta )$-approximate APSP algorithms such that $2 \alpha + \beta \ge 4$, i.e., with a multiplicative stretch $\alpha \ge 2$ or an additive stretch $\beta \ge 2$.

Apart from two notable exceptions [2, 25], all known decremental APSP algorithms fall into one of two categories. They either (a) maintain exact distances or have a relatively small multiplicative stretch of (1+ϵ) or (b) they have a stretch of at least 3. The space in between is largely unexplored in the decremental setting. This stands in sharp contrast to the static setting where for undirected graphs approximation guarantees different from multiplicative (1+ϵ) or “3 and above” have been the focus of a large body of works [24, 26–36]. The aforementioned exceptions in dynamic algorithms [2, 25], concern unweighted undirected graphs and maintain (1+ϵ,2)-approximations that simultaneously have a multiplicative error of 1+ϵ and an additive error of 2, which implies a purely multiplicative (2+ϵ)-approximation. The algorithms run in $\tilde{O}(n^{5/2})$^1^ and $n^{5/2+o(1)}$ total update time respectively. In the next section, we will detail how our algorithms improve over this total update time and generalize to weighted graphs.

Concerning the fully dynamic setting, Bernstein [5] provided an algorithm for (2+ϵ)-APSP which takes $m^{1+o(1)}$ time *per update*. That is, it cannot be directly compared to *total update time*; for example it takes $m^{2+o(1)}$ time for a series of *m* updates. Although we are not aware of any lower bounds, it seems to be hard to beat this: no improvements have been made since. The lack of progress in the fully-dynamic setting motivates the study in a partially dynamic setting, where we obtain improvements for the decremental case.

### Our Results

In this paper, we provide novel decremental APSP algorithms with approximation guarantees that previously were mostly unexplored in the decremental setting. For an overview of our results, see Table 1. Our algorithms are randomized and we assume an oblivious adversary, i.e., the sequence of updates is fixed at the start and thus independent of the random choices in the algorithm. For each pair of nodes, we can provide not only the distance estimate, but also a shortest path π of this length in $\tilde{O}(|\pi |)$ time using standard techniques, see e.g. [22]. In all our algorithms, we maintain an APSP *distance oracle*, that is, a data structure that answers APSP-queries in constant time, see Sect. 2.1 for details on the model. In this section, we assume ϵ to be a constant and hence can hide 1/ϵ factors in our asymptotic notation. Update times with ϵ as a parameter, and hence containing 1/ϵ factors, can be found in our technical sections.

(2+ϵ)-**APSP for weighted graphs.** Our first contribution is a Las Vegas algorithm for maintaining a (2+ϵ)-approximation in weighted, undirected graphs.

#### Theorem 1.1

Given a graph *G* with positive, integer weights and a constant 0<ϵ<1, there is a decremental data structure that maintains a (2+ϵ)-approximation of APSP. The algorithm has constant query time and works against an oblivious adversary. The total update time is w.h.p.

- $\tilde{O}(m^{1/2}n^{3/2}\log ^3(nW))$ if $m=n^{1+\Omega (1)}$ and $m\le n^{2-\rho }$ for an arbitrary small constant ρ,
- $\tilde{O}(m^{1/2}n^{3/2+\rho }\log ^3(nW))$ if $m\ge n^{2-\rho }$ for any constant ρ,
- $$\tilde{O}(m^{1/2}n^{3/2+o(1)}\log ^3(nW))$$ otherwise,

where *W* is the ratio between the maximum and minimum weight.

The fastest known algorithm with an approximation ratio at least as good as ours is the (1+ϵ)-approximate decremental APSP algorithm by Bernstein [11] with total update time $\tilde{O} (m n)$. With our (2+ϵ)-approximation, we improve upon this total update time when $m =n^{1+\eta }$, for any η>0. Furthermore, the fastest known algorithm with a larger approximation ratio than our algorithm is the (3+ϵ)-approximate decremental distance oracle by Ła̧cki and Nazari [20] with total update time $\tilde{O} (m \sqrt{n})$. Our result also has to be compared to the *fully dynamic* algorithm of Bernstein [5] for maintaining a (2+ϵ)-approximation that takes amortized time $m^{1 + o(1)}$ per update. Note that in *unweighted* graphs, (2+ϵ)-approximate decremental APSP algorithm can be maintained with total update time $\tilde{O} (n^{2.5})$ (which is implied by the results of Abraham and Chechik [25] and Henzinger et al. [2]). Our approach improves upon this bound for most densities, and matches it for $m=n^2$.

**Table 1:** An overview of our results and the closest related work

	Approximation	Total update time	
Weighted	1+ϵ	*nm*	[11]
	2+ϵ	$n^{3/2}m^{1/2}$	Theorem 1.1
	$(2+\epsilon ,W_{u,v})$	$nm^{3/4}$	Theorem 1.2
	3+ϵ	$n^{1/2}m$	[20]
Unweighted	(1+ϵ,2) or 2+ϵ	$n^{5/2}$	[2, 25]
	2+ϵ	$m^{7/4}$	Theorem 1.3
	(1+ϵ,2(k-1))	$n^{2-1/k}m^{1/k}$	Theorem 1.4
	$(1+\epsilon ,2(1+\tfrac{2}{\epsilon })^{k-2})$	$n^{2+1/k}$	[7]

We use the simplifying assumptions: (a) subpolynomial factors are omitted from the total update time; (b) the ratio *W* between the maximum and minimum weight is polynomial in *n*; and (c) ϵ is a constant. The combination of (a) and (c) means that 1/ϵ factors are not displayed in the total update time here

(2+ϵ,1)**-APSP for unweighted and** ${(2+\epsilon , W_{u,v})}$**-APSP for weighted graphs.** Our second contribution is a faster algorithm with an additional additive error term of 1 for unweighted graphs, which in turn can be used to obtain our unweighted (2+ϵ)-APSP result. The corresponding generalization to weighted graphs can be formulated as follows.

#### Theorem 1.2

Given a graph *G* with positive, integer weights and a constant 0<ϵ<1, there is a decremental data structure that maintains a $(2+\epsilon ,W_{u,v})$-approximation for APSP, where $W_{u,v}$ is the maximum weight on a current shortest path from *u* to *v*. The algorithm has constant query time and works against an oblivious adversary. The total update time is w.h.p. $\tilde{O}( nm^{3/4}\log ^3(nW))$ if $m=n^{1+\Omega (1)}$, and $\tilde{O}( n^{1+o(1)}m^{3/4}\log ^3(nW))$ otherwise, where *W* is the ratio between the maximum and minimum weight.

Here, *W* is a *global* parameter, while $W_{u,v}$ is *local* and depends on *u* and *v*.

(2+ϵ)**-APSP for unweighted graphs.** We obtain this result by a general reduction from mixed approximations to purely multiplicative approximations, which might be of independent interest beyond the dynamic setting. See Theorem 3.1 for the statement. We can then combine this with Theorem 1.2 to obtain a fast algorithm for non-dense unweighted graphs.

#### Theorem 1.3

Given an undirected unweighted graph *G*, there is a decremental data structure that maintains a (2+ϵ)-approximation for APSP with constant query time. The algorithm works against an oblivious adversary. W.h.p. the total update time is bounded by $\tilde{O}( m^{7/4})$ if $m=n^{1+\Omega (1)}$, and $\tilde{O}( m^{7/4+o(1)})$ otherwise.

Note that for $m=o(n^{8/7})$ this gives the first subquadratic decremental (2+ϵ)-approximate APSP algorithm. For $m\le n^{6/5}$, this approach is faster than our weighted result, Theorem 1.1, which was already beating the unweighted state-of-the-art [2, 25].(1+ϵ,2(k-1))**-APSP for unweighted graphs.** Our fourth contribution is an algorithm for unweighted, undirected graphs that maintains, for any k≥2, a (1+ϵ,2(k-1))-approximation, i.e., a distance estimate that has a multiplicative error of 1+ϵ and an additive error of 2(k-1).

#### Theorem 1.4

Given an undirected unweighted graph *G*, a constant 0<ϵ<1 and an integer $2 \le k \le \log {n}$, there is a decremental data structure that maintains (1+ϵ,2(k-1))-approximation for APSP with constant query time. The algorithm works against an oblivious adversary. The expected total update time is bounded by $\min \{O(n^{2-1/k+o(1)}m^{1/k}), \tilde{O}((n^{2-1/k}m^{1/k}) O(1/\epsilon )^{k/\rho })\}$ where $m=n^{1+\rho }$.

Note that for small values of *k* and if $m=n^{1+\rho }$ for a constant ρ>0, we get total update time of $\tilde{O}(n^{2-1/k}m^{1/k})$, and otherwise we have an extra $n^{o(1)}$ factor. In addition, in the special case of $k=\log {n}$, we get a near-quadratic update time of $O(n^{2+o(1)})$. The state-of-the-art for a purely multiplicative (1+ϵ)-approximation is the algorithm of Roditty and Zwick with total update time $\tilde{O} (m n)$.^2^ It was shown independently by Abraham and Chechik [25] and by Henzinger et al. [2] how to improve upon this total update time bound at the cost of an additional small additive error term: a (1+ϵ,2)-approximation can be maintained with total update time $\tilde{O} (n^{2.5})$. This has been generalized by Henzinger et al. [7] to an additive error term of $2 (1 + \tfrac{2}{\epsilon })^{k-2}$ and total update time $\tilde{O} (n^{2 + 1/k} O(\tfrac{1}{\epsilon })^{k-1})$. We improve upon this tradeoff in two ways: (1) our additive term is independent of 1/ϵ and *linear* in *k* and (2) our algorithm profits from graphs being sparse.

**Static follow-up work.** Interestingly, our techniques inspired a new algorithm for a static 2-approximate distance oracle [37], giving the first such distance oracle algorithm with subquadratic construction time for sparse graphs. Moreover, for m=n they match conditional lower bounds [38, 39].

### Related Work

**Static algorithms.** The baseline for the static APSP problem are the exact textbook algorithms with running times of $O (n^3)$ and $\tilde{O} (m n)$, respectively. There are several works obtaining improvements upon these running times by either shaving subpolynomial factors or by employing fast matrix multiplication, which sometimes comes at the cost of a (1+ϵ)-approximation instead of an exact result. See [40] and [41], and the references therein, for details on these approaches. For the regime of stretch 3 and more in undirected graphs, a multitude of algorithms have been developed with the distance oracle of Thorup and Zwick [42] arguably being the most well-known construction. In the following, we focus on summarizing the state of affairs for approximate APSP with stretch between 1+ϵ and 3.

In *weighted*, undirected graphs, Cohen and Zwick [27] obtained a 2-approximation with running time $$ \tilde{O} (m^{1/2} n^{3/2}) $$. This running time has been improved to $$ \tilde{O} (m \sqrt{n} + n^2) $$ by Baswana and Kavitha [28]. Employing fast matrix multiplication, Kavitha [30] provided an algorithm for (2+ϵ)-APSP that takes $$ \tilde{O} (n^{2.25}) $$ time. Subsequently, Dory et al. [37] provided two algorithms for (2+ϵ)-APSP, for dense graphs: $$ O(n^{2.214})$$ and sparse graphs: $$\tilde{O}(mn^{2/3})$$. In addition, efficient approximation algorithms for stretches of $$ \tfrac{7}{3} $$ [27] and $$ \tfrac{5}{2} $$ [30] have been obtained. These results have recently been generalized by Akav and Roditty [36] who presented an algorithm with stretch $$ 2 + \tfrac{k-2}{k} $$ and running time $$ \tilde{O} (m^{2/k} n^{2 - 3/k} + n^2) $$ for any k≥2. Moreover, Cohen and Zwick [27] obtained an $$\tilde{O}(n^{1-1/k}m^{1/k})$$-time algorithm that outputs an APSP approximation $$\hat{d}(\cdot ,\cdot )$$, which for any two vertices u,v∈V and any *u*-*v* path π satisfies $$d(u,v) \le \hat{d}(u,v) \le w(\pi ) +2\sum _{i=1}^{k-1}\omega _i$$, where $$\omega _i$$ is the *i*-th heaviest edge on π.

In *unweighted*, undirected graphs, a (1, 2) -approximate APSP algorithm with running time $\tilde{O} (n^{2.5})$ has been presented by Aingworth, Chekuri, Indyk and Motwani [26]. This has been improved by Dor, Halperin, and Zwick [24] that showed a (1, 2)-approximation with running time $\tilde{O}(\min \{n^{3/2}m^{1/2}, n^{7/3} \})$. They also show a generalized version of the algorithm that gives stretch (1, *k*) and running time ${\tilde{O}}(\min \{n^{2-{2}/{(k+2)}}m^{{2}/{(k+2)}}, n^{2+{2}/{(3k-2)}}\})$ for every even k>2. Recently, faster (1, *k*)-approximation algorithms based on fast matrix multiplication techniques were developed [43–45], here as well the fastest (1, 2)-approximation runs in $O(n^{2.260})$ time [44]. This was extended to a (1+ϵ,2)-approximation in $O(n^{2.152})$ time [37]. In addition, recently Roditty [46] extended the approach of [24] to obtain a combinatorial (2, 0)-approximation for APSP in $\tilde{O}(n^{2.25})$ time in unweighted undirected graphs. Using fast matrix multiplication, this running time was improved to $O(n^{2.032})$ [37, 45].

Berman and Kasiviswanathan [29] showed how to compute a (2, 1) -approximation in time $\tilde{O} (n^2)$. Subsequent works [28, 31–34] have improved the polylogarithmic factors in the running time and the space requirements for such nearly 2-approximations. Recently, slightly subquadratic algorithms have been given: an algorithm with stretch (2(1+ϵ),5) by Akav and Roditty [35], and an algorithm with stretch (2, 3) by Chechik and Zhang [47].

**Decremental algorithms.** The fastest algorithms for maintaining weighted, exact APSP under edge deletions have total update time $\tilde{O} (n^3)$ [3, 19, 48]. There are several algorithms that are more efficient at the cost of returning only an approximate solution. In particular, a (1+ϵ)-approximation can be maintained in total time $\tilde{O} (m n)$ [4, 11, 15]. If additionally, an additive error of 2 is tolerable, then a (1+ϵ,2)-approximation can be maintained in total time $\tilde{O} (n^{2.5})$ in unweighted, undirected graphs [2, 25]. Note that such a (1+ϵ,2)-approximation directly implies a (2+ϵ)-approximation because the only paths of length 1 are edges between neighboring nodes. All of these decremental approximation algorithms are randomized and assume an oblivious adversary. Deterministic algorithms with stretch 1+ϵ exist for unweighted, undirected graphs with running time $\tilde{O} (m n)$ [2], for weighted, undirected graphs with running time $\tilde{O} (m n^{1+o(1)})$ [18] and for weighted, directed graphs with running time $\tilde{O} (n^3)$ [16].

Decremental approximate APSP algorithms of larger stretch, namely at least 3, have first been studied by Baswana, Hariharan, and Sen [49]. After a series of improvements [4, 6, 8, 12], the state-of-the-art algorithms of [13, 20] maintain (2k-1)(1+ϵ)-approximate all-pairs shortest paths for any integer k≥2 and $0 < \epsilon \le 1$ in total update time $\tilde{O} ( (m+n^{1+o(1)})n^{1/k})$ with query time $O(\log \log (nW))$ and *O*(*k*) respectively. All of these “larger stretch” algorithms are randomized and assume an oblivious adversary.

Deterministic decremental algorithms have been developed by Chuzhoy and Saranurak [21] and by Chuzhoy [17], which have even been improved and generalized to fully dynamic algorithms. For example, Haeupler, Long, and Saranurak[50] provide worst update time $\tilde{O} (m^{\mu })$ for any constant μ and constant stretch. As observed by Mądry [51], decremental approximate APSP algorithms that are deterministic – or more generally work against an adaptive adversary – can lead to fast static approximation algorithms for the maximum multicommodity flow problem via the Garg-Könemann-Fleischer framework [52, 53]. Similarly, decremental Single Source Shortest Paths (again, deterministic or against an adaptive adversary) play a role in the recent fast min cost flow algorithms [18, 54, 55]. The above upper bounds for decremental APSP have recently been contrasted by conditional lower bounds [56] stating that constant stretch cannot be achieved with subpolynomial update and query time under certain hardness assumptions on 3SUM or (static) APSP.

**Fully dynamic algorithms.** Next, we discuss the related work on fully dynamic shortest paths. We would like to point out that all “combinatorial” algorithms discussed in the following (i.e., algorithms that do not rely on “algebraic” techniques like dynamic matrix inverse) are internally employing decremental algorithms.

The reference point in fully dynamic APSP with subpolynomial query time is the *exact* algorithm of Demetrescu and Italiano [57] with update time $\tilde{O} (n^2)$ (with log-factor improvements by Thorup [58]) and constant query time for weighted directed graphs.. Subsequently, researchers have developed algorithms with subcubic worst-case update time and constant query time [59, 60] with some of them being deterministic [61, 62]. Note that one can construct a simple update sequence for which any fully dynamic algorithm maintaining the distance matrix or the shortest-path matrix explicitly needs to perform $\Omega (n^2)$ changes to this matrix per update. Algorithms breaking the $n^2$ barrier at the cost of large query time have been obtained in unweighted directed graphs [23, 63, 64] and sparse weighted directed graph [65].

In addition to exact algorithms, combinatorial and algebraic algorithms have also been developed for the low stretch regime of (1+ϵ)-approximation. In particular, Roditty and Zwick [4] obtained the following trade-off with a combinatorial algorithm: update time $\tilde{O}(mn/t)$ and query time *O*(*t*) for any δ>0 and $t \le m^{1/2 - \delta }$. Subsequently, for $t \le \sqrt{n}$, a deterministic variant was developed [66] and it was generalized to weighted directed graphs [11]. Furthermore, by a standard reduction (see e.g. [67]) using a decremental approximate single-source shortest paths algorithm [8, 18], one obtains a combinatorial deterministic algorithm with update time $O (n m^{1+o(1)} / t)$ and query time *O*(*t*) for any t≤n, for the fully dynamic all-pairs problem in weighted undirected graphs. Algebraic approaches can achieve subquadratic update time and sublinear query time, namely worst-case update time $O(n^{1.863})$ and query time $O (n^{0.666})$ in weighted directed graphs [68], or worst-case update time $O(n^{1.788})$ and query time $O (n^{0.45})$ in unweighted undirected graphs [69]. In directed graphs, van den Brand and Karczmarz [70] provided explicit APSP with $O(n^{2.293}/\varepsilon )$ worst case update time for unweighted graphs.

Several fully dynamic distance oracles have been developed that obtain a larger stretch of 2+ϵ. In particular, Bernstein [5] developed a distance oracle with update time $O (m^{1 + o(1)})$ and query time $O (\log \log \log n)$ in weighted undirected graphs. It can be made deterministic using the deterministic approximate single-source shortest path algorithm by Bernstein, Probst Gutenberg, and Saranurak [18]. The only conditional lower bound in this regime that we are aware of states that no algorithm can maintain a (3-ϵ)-approximation with update time $O (n^{1/2-\delta })$ and query time $O (n^{1-\delta })$ for any δ>0, unless the OMv conjecture fails [10].

In the regime of stretch at least 3, the following trade-offs between stretch and update time have been developed: Abraham, Chechik, and Talwar [12] designed an algorithm for unweighted undirected graphs with stretch $2^{O (\rho k)}$, update time $\tilde{O} (m^{1/2} n^{1/k})$, and query time $O (k^2 \rho ^2)$, where k≥1 is a freely chosen parameter and $\rho = 1 + \lceil \log n^{1-1/k} / \log (m / n^{1-1/k}) \rceil$. Forster, Goranci, and Henzinger [71] designed an algorithm for weighted undirected graphs with stretch $O (\log n)^{3 k - 2}$, update time $m^{1/k + o(1)} \cdot O(\log n)^{4k - 2}$, and query time $O (k \log ^2 n)$, where k≥2 is an arbitrary integer parameter. Further, we note that any algorithm whose update time depends on the sparsity of the graph (possibly also a static one) can be run on a spanner of the input graph maintained by a fully dynamic spanner algorithm [72]. Finally, recent works [50, 73] have developed deterministic fully dynamic distance oracles. In particular, the work of Kyng, Meierhans, and Probst Gutenberg [73] achieves an $n^{o(1)}$ approximation ratio with $n^{o(1)}$ worst-case update time and polylogarithmic query time. Meanwhile, the work of Haeupler, Long, and Saranurak [50] provides a constant-factor approximation with arbitrarily low polynomial worst-case update time and polylogarithmic query time.

### Outline

We give an overview of the notation, terminology, and various decremental tools we use in Sect. 2. Next, we give a high-level overview of our techniques and provide an intuition for our algorithms in Sect. 3. Then follow three technical sections. In Sect. 4, we prove Theorem 1.1. Then we prove Theorem 1.2 and 1.3 in Sect. 5. Lastly, we prove Theorem 1.4 in Sect. 6.

## Preliminaries

### Notation and Terminology

Throughout this paper, neighbors of a node *v* are denoted by *N*(*v*). We denote by $d_G(u,v)$ the distance between *u* and *v* in the graph *G*. If *G* is clear from the context, we use the notation *d*(*u*, *v*). When *G* is a weighted graph, we allow for positive, integer weights. We denote by *W* the ratio between the maximum and minimum weight of the graph. Moreover, we denote by $W_{u,v}$ the maximum weight on a shortest path from *u* to *v*. In particular, $W_{u,v} \le W$ and hence additive approximations in terms of $W_{u,v}$ can be tighter than in terms of *W*.

We are interested in designing *dynamic graph algorithms* for shortest path problems, which support both update and query operations. In particular, we look at *decremental* algorithms, which support weight increases and edge deletions. This is opposed to *fully dynamic* algorithms, which also support weight decreases and edge insertions. To be concrete, we support the following three operations:

- Delete(*u*, *v*): delete the edge $\{u,v\}$ from the graph;
- Update(u,v,Δ): increase the weight of an edge $\{u,v\}$ to Δ, i.e., $w(u,v)\leftarrow \Delta$;
- Distance(*u*, *v*): return a distance approximation $\hat{d}_G(u,v)$ between the nodes *u* and *v* in the current graph *G*.

We assume that we have an *oblivious adversary*, i.e., that the sequence of updates is fixed from the start. This is as opposed to the *adaptive adversary*, which can use the random choices made by the algorithm to determine the next update. More formally, let $S_t$ denote the state of the algorithm after update *t* and let $R_t$ be the random bits used in this update step. An oblivious adversary chooses the entire update sequence $\sigma = (\sigma _1, \dots , \sigma _m)$ before execution begins, and hence purely based on the algorithm’s description and independent of the algorithm’s states $S_t$ and random bits $R_t$. An adaptive adversary selects each input $\sigma _t$ sequentially based on the algorithm’s description, the history of the algorithm’s past states $S_1, \dots S_{t-1}$, and the used random bits $R_1, \dots R_{t-1}$. Since an adversary can determine future states of a deterministic algorithm, any correct deterministic algorithm holds against an adaptive adversary.

Edge weights can be positive integers bounded by *W*, for some parameter $W\in \mathbb {N}$. We say that $\texttt {Distance}(()u,v)$ returns $(\alpha ,\beta )$*-approximate distances*, denoted by $\hat{d}_G$, if $d_G(u,v) \le \hat{d}_G(u,v) \le \alpha d_G(u,v)+\beta$ for all u,v∈V. Moreover we denote (α,0)-approximate by α-approximate. Throughout, we denote $\hat{d}_G$ for the output of our algorithms, and δ (potentially with subscript) for approximate distances used internally.

In all our algorithms, we maintain a *distance oracle* rather than explicit APSP. This means that we maintain a data structure that can answer distance queries $\texttt {Distance}(u,v)$ for any u,v∈V. The time to answer a distance query $\texttt {Distance}(u,v)$ is called the *query time*. All our algorithms use constant query time. Further, we give the *total update time*, which is the time needed to process a sequence of up to *m* edge deletions or weight increases, where *m* is the number of edges before the first deletion.

For any object, value, or data structure *x* that changes over time, we use a superscript $x^{(t)}$ to specify its state at time *t*, i.e., $x^{(t)}$ is *x* after processing *t* updates.

### Bunches and Clusters

We start by reviewing the concepts of bunch and cluster as defined in the seminal distance oracle construction of Thorup and Zwick [42]. Given a parameter p∈[0,1], called the *cluster sampling rate*, we let *A* be a set of sampled nodes, where each node is sampled uniformly at random with probability *p*. Then we define the *pivot* of a node *v* to be the closest node in *A*, i.e., $p(v)=p_A(v)=\mathop {\mathrm {arg\,min}}\limits _{s\in A} d(s,v)$. Since our algorithms contain only one level of sampling, and thus *A* we will be fixed throughout, we omit the subscript *A* henceforth. The *bunch* *B*(*v*) of a node *v* is the set $B(v):=\{ u \in V: d(u,v) < d(v,p(v)) \}$ and the *clusters* are $C(u):= \{ v \in V: d(u,v) < d(v,p(v)) \}=\{v \in V: u\in B(v)\}$. Hence bunches and clusters are inverses of each other: u∈B(v) if and only if v∈C(u).

Thorup and Zwick showed how to compute bunches and clusters in the static setting in $\tilde{O}(\tfrac{m}{p})$ time [42]. However, it is challenging to maintain them exactly under deletions: for starters, we cannot compute exact distances from the pivots efficiently. In the static setting, we can compute such distances in $\tilde{O}(pnm)$ time, by running Dijkstra’s algorithm from every pivot. In the decremental setting, we only have efficient exact shortest path computation (through ES-trees, defined in Sect. 2.4) when we restrict to *bounded depth*, which is not sufficient for our applications. It is possible to maintain *approximate bunches* that can deal with these challenges. We note that Roditty and Zwick [4] provide an algorithm that is efficient for maintaining decremental bunches *exactly*, but again only up to bounded depth, see Lemma B.1. So, this is too expensive for our purpose. Instead we use another type of approximate bunches proposed by Łącki and Nazari [20]. We start by relaxing the definition of the pivot: the approximate pivot $\tilde{p}(v)\in A$ of a node *v* does not have to be the closest node in *A*, but just within a (1+ϵ) factor of it:

$$\begin{aligned} d_G(v,p(v)) \le \delta _{\tilde{B}}(v,\tilde{p}(v)) \le (1+\epsilon ) d_G(v,p(v)). \end{aligned}$$

Next, we define an approximate bunch $\tilde{B}(v)$, which rather than containing the exact bunch *B*(*v*) contains all nodes that are at least a factor 1/(1+ϵ) closer than the exact pivot *p*(*v*). More precisely, for our analysis, our approximate bunch $\tilde{B}(v)$ needs to contain all nodes $\{ w \in V: (1+\epsilon )d_G(v,w) < d_G(v, p(v)) \}$. More precisely, in the stretch analysis of Theorem 1.1 (see Lemma 4.8), we need that if $w\notin \tilde{B}(v)$, that $d_G(v,w) \ge \tfrac{d_G(v,p(v))}{1+\epsilon }$, i.e., that nodes *not* in the bunch are far enough away. Since we can lose a factor (1+ϵ) in our distance estimates, we do not need that the nodes are actually farther away than the pivot (this would mean that the entire bunch $B(v)\subseteq \tilde{B}(v)$), but it suffices that all nodes sufficiently closer than the pivot are in there. It can also contain additional nodes. For efficiency purposes, we still want the approximate bunch to remain small, say only polylogarithmically larger than the exact bunch *B*(*v*).

Finally, for every node v∈V and every node $u\in \tilde{B}(v)$, we need to maintain a distance estimate $\delta _{\tilde{B}}(u,v)$. Here also an approximate distance will suffice:

$$\begin{aligned} d_G(u,v) \le \delta _{\tilde{B}}(u,v) \le (1+\epsilon ) d_G(u,v). \end{aligned}$$

The following lemma provides these approximate objects. It is implicitly proven by Łącki and Nazari [20], but for completeness we sketch a proof in Appendix B. The lemma outputs: approximate pivots, approximate bunches, and a distance estimate $\delta _{\tilde{B}}(u,v)$ for v∈V and $u\in \tilde{B}(v)$.

#### Lemma 2.1

Let G=(V,E,w) be a decremental graph, and let 0<ϵ<1 and 0<ρ<1/2 be parameters, let 0<p<1 be a sampling probability, and let A⊆V be a set of sampled nodes, where each element of *V* is sampled uniformly at random with probability *p*. There exists an algorithm that maintains for each v∈V: (i) an approximate pivot $\tilde{p}(v)\in A$; (ii) an approximate bunch $\tilde{B}(v)$; and (iii) distance estimates $\delta _{\tilde{B}}(v,u)$ for every $u\in \tilde{B}(v)$. These objects satisfy:

- $$d_G(v,p(v)) \le \delta _{\tilde{B}}(v,\tilde{p}(v)) \le (1+\epsilon ) d_G(v,p(v))$$;
- $$ \{ w \in V: (1+\epsilon )d_G(v,w) < d_G(v, p(v)) \}\subseteq \tilde{B}(v) $$;
- $\delta _{\tilde{B}}$ is monotone (non-decreasing over updates);
- for any $u \in \tilde{B}(v)$: $d_G(u,v) \le \delta _{\tilde{B}}(u,v) \le (1+\epsilon ) d_G(u,v)$; and
- with high probability, $|\tilde{B}(v)| = O\left( \frac{\log n\log nW}{p}\right)$.

The algorithm has total update time $\tilde{O}(\tfrac{mn^\rho }{p}\cdot \log ^2(nW)) \cdot O(\frac{1}{\rho \epsilon })^{1+2/\rho }$.

The approximate cluster is the reverse of the approximate bunch: $\tilde{C}(u):=\{v \in V: u\in \tilde{B}(v)\}$.

In our algorithms for balancing out different terms in the running time we set the parameter ρ depending on the graph density so that $m= n^{1+\rho }$. Hence for graphs with $m= n^{1+\Omega (1)}$ edges, we can set ρ to be a constant and for sparser graphs we can set $\rho = \frac{\sqrt{\log n}}{\log \log n}$. For simplicity of our statements in the introduction (Sect. 1) and the overview (Sect. 3) we assume 0<ϵ<1 is a constant, and thus we hide $(1/\epsilon )^c$ factors in $\tilde{O}$ when *c* is constant, or in $n^{o(1)}$ when c=ω(1).

### Decremental Multi-Source Shortest Paths

We will use the following lemma from Łącki and Nazari [20] to maintain (1+ϵ)-approximate distances for S×V, for *S* some given set of sources.

#### Lemma 2.2

([20]) There is a data structure which given a weighted, undirected graph G=(V,E) explicitly maintains (1+ϵ)-approximate distances from a set of sources *S* in *G* under edge deletions, where 0<ϵ<1/2 is a parameter. After each update, the algorithm returns all nodes for which the distance estimate has changed. During the execution of the algorithm, nodes can be added to *S*. We write *s* for the final size of *S*. Assuming that $|E| = n^{1+\Omega (1)}$ and $s = n^{\Omega (1)}$, the total update time is w.h.p. $\tilde{O}(sm/\epsilon )$. If $|E| = n^{1+o(1)}$ or $s= n^{o(1)}$, then the total update time is $\tilde{O}\left( smn(1/\epsilon )^{\tilde{O}(\sqrt{\log n})}\right)$.

Here the factor $(1/\epsilon )^{\tilde{O}(\sqrt{\log n})}$ again simplifies to $n^{o(1)}$ for constant ϵ.

### ES-Trees

An Even-Shiloach tree (ES-tree) is a central data structure for maintaining distances in a decremental setting. It maintains the exact distances from a source *s* under edge deletions, giving the following.

#### Lemma 2.3

([74–76]) There is a data structure called ES-tree that, given an integer-weighted directed graph *G* undergoing deletions and edge weight increases, a root node *s*, and a depth parameter *d*, maintains, for every node *v* a value $\ell _s(v)$ such that $\ell _s(v) = d_G(s,v)$ if $d_G(s,v) \le d$ and $\ell _s(v) = \infty$ if $d_G(s,v) > d$. It has constant query time and a total update time of O(md+n), where *m* is the number of edges in *G*.

A *monotone* ES-tree is a generalization of ES-tree studied in [2, 8, 14, 77] that handles edge insertions. This is a data structure that maintains distances from a source *s* in a graph undergoing deletions, insertions and edge weight increases. Note that when edges are inserted to the graph, it can potentially decrease some distances in the graph. However, the data structure ignores such decreases, and maintains distance estimates $\ell _s(v)$ from *s* to any other node, that can only *increase* during the algorithm. In particular, these distance estimates are not necessarily the correct distances in the graph, and when using this data structure the goal is to prove that these estimates still provide a good approximation for the current distances. We call $\ell _s(v)$ the level of *v* in the monotone ES-tree rooted at *s*.

We next describe the main properties of monotone ES-trees that we use in our algorithms. A pseudo-code appears in Appendix A. The following lemma follows from [2] and from the pseudo-code.

#### Lemma 2.4

The following holds for a monotone ES-tree of a graph *H* rooted at *s*, that is maintained up to distance *d*:

1. At the beginning of the algorithm, $\ell _s(v)=d_H(s,v)$ for all nodes *v* where $d_H(s,v) \le d$.
2. It always holds that $d_H(s,v) \le \ell _s(v)$.
3. The level $\ell _s(v)$ of a node *v* never decreases.
4. When the level $\ell _s(v)$ increases to a value that is at most *d*, we have $\ell _s(v) = \min \limits _{x\in N(v)} \{\ell _s(x)+w_{H}(x,v) \}$.

#### Lemma 2.5

([2]) For every d≥1, the total update time of a monotone ES-tree up to maximum level *d* on a graph *H* undergoing edge deletions, edge insertions, and edge weight increases is $O(\mathcal {E}(H)\cdot d+\mathcal {W}(H)+n)$, where $\mathcal {E}(H)$ is the total number of edges ever contained in *H* and $\mathcal {W}(H)$ is the total number of updates to *H*.

### Near-Additive Decremental APSP

In [7], the authors show an algorithm that obtains near-additive approximations for decremental APSP. This result will be used to handle certain cases in Sect. 6.

#### Theorem 2.6

(Theorem 1.3 in [7]) For any constant 0<ϵ<1 and integer $2 \le k \le \log {n}$, there is a $(1+\epsilon , 2(1+2/\epsilon )^{k-2})$-approximation algorithm for decremental APSP with constant worst-case query time and expected total update time of $\tilde{O}(n^{2+1/k} (37/\epsilon )^{k-1})$.

### Notation Overview and Choice of Parameters

To guide the reader, we give an overview of how notation is used in this paper. Whenever we deviate from these assumptions, this is clearly stated in the text.

Generally, superscripts $\cdot ^{(t)}$ are used to denote a value or object at time step *t*. For readability, this superscript is omitted when it is not necessary. We denote the exact distance from *u* to *v* by *d*(*u*, *v*). As defined in Sect. 2.4, $\ell _s(v)$ denotes the level of *v* in the (monotone) ES-tree rooted at *s*. Hence $\ell _s(v)=d(s,v)$ maintains the exact distance. We denote any (1+ϵ)-approximate distance estimate by δ. It can be equipped with a subscript to denote the origin of the approximation. Given some value *w*, we write $\tilde{w}$ to denote a (1+ϵ) approximation that changes at most $O(\log _{1+\epsilon } (nW))$ times. Formally, this is achieved by setting $\tilde{w} = (1+\epsilon )^{\lceil \log _{1+\epsilon } w\rceil }$. Similarly, $\tilde{\delta }$ is a (1+ϵ)-approximate distance that only changes $O(\log _{1+\epsilon } (nW))$ times. Finally, we denote the approximate APSP that the algorithm outputs by $\hat{d}$, e.g., a (2+ϵ)-approximation of APSP.

## High-Level Overview

In this section we provide a high-level overview of our algorithms. First we describe our (2+ϵ)-APSP algorithms for weighted graphs, by giving a simpler static version first. Then we describe our $(2+\epsilon ,W_{u,v})$-APSP algorithm, where we introduce a notion of *bunch overlap threshold* to overcome the challenge of dynamically maintaining a well-known static adaptive sampling technique [78] (see Sect. 3.2). We give a reduction from mixed approximations to purely multiplicative approximations, which together with the previous result gives our (2+ϵ)-APSP algorithm for unweighted graphs. Finally, we describe our (1+ϵ,2(k-1))-APSP algorithm for any k≥2 in unweighted graphs.

As in Sect. 1, we assume ϵ to be a constant in this section. Hence, we can hide 1/ϵ factors in our asymptotic notation. Update times with ϵ as a parameter, and hence containing 1/ϵ factors, can be found in our technical sections. In all cases, these factors are inherited from the use of decremental *single source shortest path (SSSP)* algorithms (see, e.g., [79]), and hence do not form a critical part of our paper.

### (2+ϵ)-APSP for Weighted Graphs

We first present a warm-up static algorithm that we later turn into a dynamic algorithm.

#### Static 2-APSP

We consider the bunches and clusters of the well-known distance oracles of Thorup and Zwick [42], as introduced in Sect. 2.2. In particular, we consider sampling probability *p*, set of sampled nodes *A*, and bunches *B*(*v*) and clusters *C*(*v*) for v∈V. We store the distances A×V, together with the clusters and bunches of each node. Moreover, we store the distances *d*(*u*, *v*) for all u∈V and v∈B(u). With this information, we can query 3-approximate distances for any pair $u,v\in V\times V$. At a high-level, this works as follows. We either have v∈B(u) and so we explicitly have stored their distance, or we can get a 3-approximate estimate by computing d(u,p(u))+d(p(u),v), since

$$\begin{aligned} d(u,p(u))+d(p(u),v) \le d(u,p(u))+d(u,p(u))+d(u,v) \le 3d(u,v), \end{aligned}$$

where the first step follows by the triangle inequality and the second step by the fact that d(u,p(u))≤d(u,v) if v∉B(u). In the following, we explain that in some special cases we can directly use the estimate d(u,p(u))+d(p(u),v) to obtain a 2-approximation for a pair *u*, *v*. In other cases, we can store more information depending on how *B*(*u*) and *B*(*v*) overlap and use this to obtain a 2-approximation to *d*(*u*, *v*).

Let us assume that for all nodes *v* we have computed *B*(*v*) and have access to all the distances from *v* to each node u∈B(v). Also assume that we have computed the distances from the set *A* to all nodes. We now describe how using this information we can get a 2-approximate estimate of *any* pair of nodes *u*, *v* in the first case. In another case we will discuss what other estimates we need to precompute. Let π be a shortest path between *u* and *v*. We consider two cases depending on how the bunches *B*(*u*) and *B*(*v*) interact with π. A similar case analysis was used in [80, 81] in distributed approximate shortest paths algorithms.

**Fig. 1:**
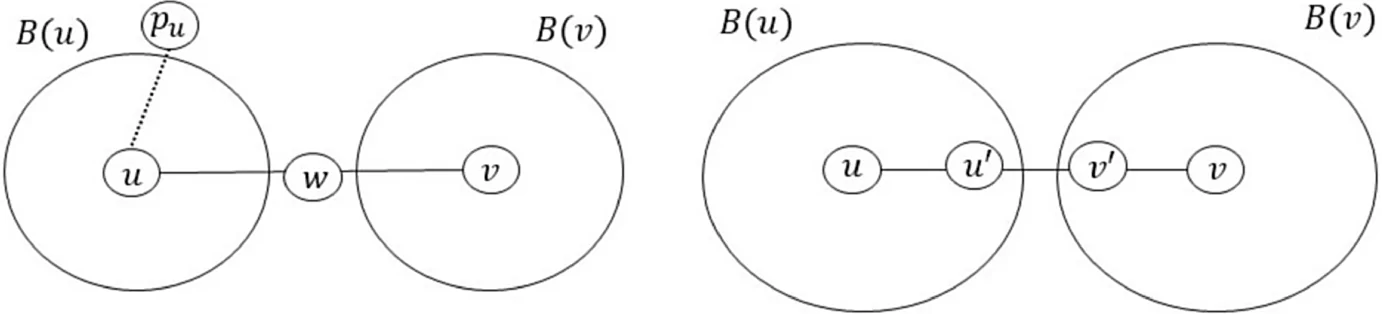
Possible scenarios for the overlap between the bunches *B*(*u*), *B*(*v*) and a shortest path π from *u* to *v*

1. There exists $w \in \pi$ such that $w \not \in B(u) \cup B(v)$ (left case in Fig. 1): Since *w* is on the shortest path π, we either have d(w,u)≤d(u,v)/2 or d(v,w)≤d(u,v)/2. Suppose d(w,u)≤d(u,v)/2. We observe that by definition d(u,p(u))≤d(u,w), hence we obtain $d(u,p(u))+d(p(u),v)\le d(u,p(u))+d(u,p(u))+d(u,v) \le 2d(u,v)$, where the first step follows by the triangle inequality and the second step by the fact that $d(u,p(u))\le d(u,w)\le d(u,v)/2$. The case that d(v,w)≤d(u,v)/2 is analogous, and hence by computing $\min \{ d(u,p(u))+d(p(u),v), d(v,p(v))+d(p(v),u)\}$ we get a 2-approximation.
2. There exists no $$w \in \pi $$ such that $$w \not \in B(u) \cup B(v)$$. In other words, $$(B(u) \cup B(v)) \cap \pi = \pi $$ and hence there exists at least one edge $$\{u',v'\}$$ on π where $u^{\prime }\in B\left(u\right)$ and $v^{\prime }\in B\left(v\right)$ (right case in Fig. 1,^3^): in this case we can find the minimum over estimates obtained through all such pairs $u^{\prime },v^{\prime }$, i.e. by computing $d\left(u,u^{\prime }\right)+w\left(u^{\prime },v^{\prime }\right)+d\left(v^{\prime },v\right)$. We note that this case includes the arguably easier situation that the bunches overlap, i.e., $$B(u)\cap B(v)\ne \emptyset $$ (not reflected in the picture).

If we had access to all the above distances for the bunches and the pivots, we could then use them to query 2-approximate distances between any pair u,v∈V. As we will see, in our algorithms we only have *approximate bunches and pivots* which lead to (2+ϵ)-approximate queries.

**Running time.** We can compute bunches and clusters in $$\tilde{O}(\tfrac{m}{p})$$ time [42]. We need to compute shortest paths from the set of pivots *A* for the estimates in Case 1. This takes $$O(|A|m)=\tilde{O}(pnm)$$ time using Dijkstra’s algorithm. Finally, we consider Case 2. A straight-forward approach is to consider each pair of vertices *u*, *v*, and all $u^{\prime }\in B\left(u\right)$ and $v^{\prime }\in B\left(v\right)$. This takes $$O(\tfrac{n^2}{p^2})$$ time.

We use an intermediate step that computes the distance between $u^{\prime }$ and *v* if there is an edge $$\{u',v'\}\in E$$ such that $v^{\prime }\in B\left(v\right)$. For each node v∈V, we consider all $v^{\prime }\in B\left(v\right)$, for which we consider all neighbors $u^{\prime }\in N\left(v^{\prime }\right)$ and compute the estimate $w\left(u^{\prime },v^{\prime }\right)+d\left(v^{\prime },v\right)$ (and replace current $u^{\prime }-v$ minimum if it is smaller). Since $$|B(v)|=\tilde{O}(\tfrac{1}{p})$$, we can compute these estimates for all $d(u^{\prime },v)$ in $$\tilde{O}(\tfrac{n^2}{p})$$ time.

Next, for each pair u,v∈V, we take the minimum over the distances $d\left(u,u^{\prime }\right)+d\left(u^{\prime },v\right)$ for all $u^{\prime }$ in the bunch *B*(*u*) using the precomputed distances from the previous step in time $$\tilde{O}(n^2/p)$$, by iterating over all pairs u,v∈V, and all $u^{\prime }\in B\left(u\right)$. So in total the adjacent case takes time $$\tilde{O}(\tfrac{n^2}{p})$$. Hence the total running time of the algorithm will be $$\tilde{O}(pnm+m/p+n^2/p)=\tilde{O}(pnm+n^2/p)$$. By setting $$p=\sqrt{\tfrac{n}{m}}$$ we obtain the total update time $$\tilde{O}(m^{1/2}n^{3/2})$$.

There are several subtleties that make it more difficult to maintain these estimates in decremental settings. Not only do distances change, but also which nodes are part of a bunch change due to the updates. Moreover, which bunches are adjacent to each other changes. We next discuss how these intricacies can be handled. We obtain a total update time that matches the stated static running time (up to subpolynomial factors).

#### Dynamic Challenges

**Maintaining bunches and pivots.** First, we need to dynamically maintain bunches efficiently as nodes may join and leave a bunch throughout the updates using an adaptation of prior work. One option would be maintaining the clusters and bunches using the [4] framework, however using this directly is slow for our purposes. Hence we maintain *approximate* clusters and bunches using hopsets of Łącki and Nazari [20]. This algorithm also maintains approximate pivots, i.e. pivots that are within (1+ϵ)-approximate distance of the true closest sampled node. These estimates let us handle the first case (up to (1+ϵ) approximation). One subtlety is that the type of approximate bunches used in [20] is slightly different from the type of approximate bunches we need for other parts of our algorithm. Specifically, we need to bound the number of times nodes in a bunch can change. Roughly speaking, we can show this since the graph is decremental and the estimates obtained from the algorithm of [20] are monotone. Hence we perform *lazy bunch updates*, where we only let a bunch grow if the distance to the pivot grows by at least a factor 1+ϵ. This means a bunch grows at most $O(\log _{1+\epsilon }(nW))$ times. In addition, these approximate bunches need to be taken into account in our stretch analysis.

We note that (1+ϵ)-approximate decremental SSSP takes $\tilde{O}(m^{1+o(1)})$ total update time. However, we use the multi-source shortest path result from [20], where the $n^{o(1)}$-term vanishes when applied with polynomially many sources, giving us a total update time of $\tilde{O}(|A|m)$.

**Maintaining estimates for Case 2.** When we are in Case 2, we need more tools to keep track of estimates going through these nodes since both the bunches and the distances involved are changing. In particular, we explain how by using heaps in different parts of our algorithm we get efficient update and query times. Moreover, note that we have an additional complication: there are two data structures in this step, where one impacts the other. We need to ensure that an update in the original graph does not lead to many changes in the first data structure, which all need to be processed for the second data structure.

To be precise, we need intermediate data structures $$Q_{u',v}$$ that store approximate distances for $u^{\prime }$ and *v* of the form $w\left(u^{\prime },v^{\prime }\right)+\delta \left(v^{\prime },v\right)$ for each $v^{\prime }\in B\left(v\right)$ such that $$\{u',v'\}\in E$$, as explained for the static algorithm. The static algorithm used the exact distance $d(v^{\prime },v)$, while here we use an estimate: throughout this section (Sect. 3.1.2), $$\delta (\cdot ,\cdot )$$ denotes (1+ϵ)-approximate distances in our dynamic data structures. Also as opposed to the static algorithm, we do not only keep the minimum, but instead maintain a min-heap, from which the minimum is easily extracted.

However, this approach has a problem: $\delta (v^{\prime },v)$ might change many times, and for each such change we need to update at most $|N(v^{\prime })|\leq n$ heaps. To overcome this problem, we use another notion of *lazy distance update*: we only update a bunch if the estimate of a node changes by at least a factor 1+ϵ. Combining this with the fact that, due to the lazy *bunch* update, nodes only join a cluster at most $$O(\log _{1+\epsilon }(nW))$$ times, and thus there is only an $$ O(\log _{1+\epsilon }(nW)\log (nW))$$ overhead in maintaining these min-heaps $$Q_{u',v}$$ dynamically due to bunch updates. Note that at this stage, it is not relevant which node is declared the pivot: only the distance to the pivot influences the bunch.^4^

Similarly, $w(u^{\prime },v^{\prime })$ might change many times, which has an impact for every $v\in C(v^{\prime })$. Since this can be many nodes, we need to use an additional trick so that an adversary cannot increase our update time to *mn*. Again the solution is a lazy update scheme: instead of $w(u^{\prime },v^{\prime })$ we use $$\tilde{w}(u', v')=(1+\epsilon )^{\lceil \log _{1+\epsilon } w(u',v')\rceil }$$, which can only change $$\log _{1+\epsilon } W$$ times. This comes at the cost of a (1+ϵ) approximation factor.

We note that our bunches are approximate bunches in three different ways. We have one notion of approximation due to the fact that we are using hopsets (that implicitly maintain bunches on scaled graphs). We have a second notion of approximation due to our lazy bunch update, which only lets nodes join a bunch a bounded number of times. We have a third notion of approximation due to the lazy distance update, which only propagates distance changes O(log(nW)) times. We need to carefully consider how these different notions of approximate bunch interact with each other and with the stretch of the algorithm.

Next, we want to use the min-heaps $$Q_{u',v}$$ to maintain the distance estimates for u,v∈V (see again the right case in Fig. 1). We construct min-heaps $$Q'_{u,v}$$, for each pair *u*, *v* with entries $$\delta (u,u')+\delta (u',v)$$ for $u^{\prime }\in B\left(u\right)$. Here $\delta (u^{\prime },v)$ is the minimum from the intermediate data structure $$Q_{u',v}$$.

An entry $$\delta (u,u')+\delta (u',v)$$ in $$Q'_{u,v}$$ has to be updated when either $\delta (u,u^{\prime })$ or $\delta (u^{\prime },v)$ change. We have to make sure we do not have to update these entries too often. For the first part of each entry, we bound updates to the distance estimates $\delta (u,u^{\prime })$ by the lazy distance updates. One challenge here is that when the distance estimates in a bunch change we need to update all the impacted heaps i.e. if $\delta (u,u^{\prime })$ inside a bunch *B*(*u*) changes, we must update all the heaps $$Q'_{u,v}$$ such that there exists $$\{u',v'\} \in E$$ with $v^{\prime }\in B\left(v\right)$. For this purpose one approach is the following: for each $$u \in V,u' \in B(u)$$ we keep an additional data structure $$\text {Set}_{u,u'}$$ that stores for which v∈V we have $v^{\prime }\in B\left(v\right)$ with $$\{u',v'\}\in E$$. This means that given a change in $\delta (u,u^{\prime })$, we can update each of the heaps in logarithmic time.

For the second part of the entry, which is the distance estimate $\delta (u^{\prime },v)$ from the first data structure, this is more complicated. Another subtlety for our decremental data structures is the fact that $\delta (u^{\prime },v)$ may also decrease due to a new node being added to *B*(*v*). However, our lazy bunch update ensures that $\delta (u^{\prime },v)$ can only decrease $$O(\log _{1+\epsilon }(nW))$$ times. Further, we use lazy distance increases *in between* such decreases. Hence in total we propagate changes to $\delta (u^{\prime },v)$ at most $$O(\log _{1+\epsilon }(nW)\log (nW))$$ times, and thus our decremental algorithm has $$\tilde{O}(\log _{1+\epsilon }(nW)\log (nW))$$ overhead compared to the static algorithm.

### $(2+\epsilon , W_{u,v})$-APSP for Weighted Graphs

For a moment, let us go back to the first static algorithm. It turns out, that if instead of computing an estimate for two ‘adjacent’ bunches, we keep an estimate for bunches that overlap in at least one node, we obtain a $(2, W_{u,v})$-approximation, where $W_{u,v}$ is the maximum weight on a shortest path from *u* to *v*.

**Fig. 2:**
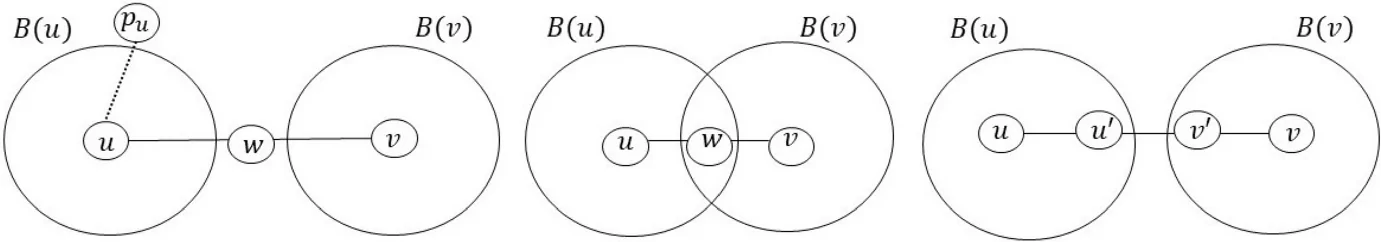
Possible scenarios for the overlap between the bunches *B*(*u*) and *B*(*v*) and a shortest path π from *u* to *v*

We fix an arbitrary shortest path from *u* to *v* denoted by π. The stretch analysis comes down to the following cases:

1. There exists no $w\in \pi \cap B(u)\cap B(v)$ (the left and right case in Fig. 2).
2. There exists $w\in \pi$ such that $w\in B(u)\cap B(v)$ (the middle case in Fig. 2).

The crucial observation here is that the distance estimate via the pivot (Case 1 in the previous section) already gives a $(2, W_{u,v})$-approximation for the adjacent case (the right case in Fig. 2), see Sect. 5.1 for the details. So if we maintain an estimate for *d*(*u*, *v*) if there exists $w\in \pi$ such that $w\in B(u)\cap B(v)$ (the ‘overlap case’), then we obtain a $(2, W_{u,v})$-approximation in total.

Here we need to create a min-heap for the overlap case, later we show we can maintain it in $\tilde{O}(nC_{\max }/p)$ time, where $C_{\max }$ is the size of the largest cluster. First we focus on the challenge of bounding $C_{\max }$.

**Efficiency challenge.** While the bunches are small, there is no bound on the size of the clusters. To overcome this issue in the static case, Thorup and Zwick developed an alternative way to build the bunches and clusters, that guarantees that the clusters are also small [78]. At a high-level, their approach is to adaptively change (grow) *A* by sampling big clusters into smaller clusters using a sampling rate proportional to the cluster size and adding these nodes to the set of pivots *A*. In total, in $\tilde{O}(m/p)$ time we obtain bunches and clusters, *both* of size $\tilde{O}(1/p)$. This idea was developed in the context of obtaining compact routing schemes, and later found numerous applications in static algorithms for approximating shortest paths and distances (see e.g., [28, 35, 36, 82]).

Using this trick, the static algorithm has running time $\tilde{O}(\tfrac{m}{p}+pnm+\tfrac{n}{p^2})=\tilde{O}(nm^{3/4}+m^{4/3})$, for $p=m^{-1/3}$ or $\tilde{O}(mn^{1/2}+n^2)$ for $p=n^{-1/2}$.

However, the adaptive sampling procedure does not seem to be well-suited to a dynamic setting. The reason is that the sampling procedure is adaptive in such a way that when the bunches/clusters change, the set of sampled nodes can also change. Although (a dynamic adaptation of) the algorithm can guarantee that the set of sampled nodes is small at any particular moment, this would require to both add nodes to and remove nodes from *A* at any time. This would add a significant amount of computation necessary to propagate these changes to other parts of the algorithm. A possible solution is to enforce *A* to only grow, by never letting nodes leave *A*. This would require us to bound the *total* number of nodes that would ever be sampled, which seems impossible with the current approach. In that approach, we could only bound the number of new nodes for a single deletion, which can become too larger after many deletions. To overcome this issue, we next suggest a new approach that gives us a set that is only increasing in size, but remains bounded. For simplicity, we first present this in a static setting.

**Introducing bunch overlap thresholds.** Our proposed approach is to divide the nodes into two types depending on the number of bunches they appear in. In particular, we consider a threshold parameter τ and we define a node to be a *heavy node* if it is in more than τ bunches (equivalently its cluster contains more than τ nodes), and otherwise we call it a *light node*. In other words, for the set of light nodes we have $C_{\max }\le \tau$. This threshold introduces a second type of ‘pivot’ for each node *u*, which we denote by *q*(*u*) that is defined to be the closest heavy node to *u*. In particular, instead of computing all distance estimates going through nodes $w \in B(u) \cap B(v)$, we only compute them for the case that *w* is light, and otherwise it is enough to compute the minimum estimates going through the closest heavy node, i.e. $\min \{ d(u,q(u))+d(q(u),v), d(v,q(v))+d(q(v),u)\}$. We emphasize that although the heavy nodes fullfill a similar role to the pivots, they are *not a subset* of our pivots p(v),v∈V. Moreover, heavy nodes have a different behavior in bounding the running time than the pivots. As we will see later, introducing these thresholds is crucial in efficiently obtaining the estimates required for Case 2 as we can handle the light and heavy case separately.

**Stretch of the algorithm with bunch overlap thresholds.** As discussed above, the stretch analysis was divided into two cases. In the new variant of the algorithm we add a third case, where we look at the distance estimates going through the closest heavy node, i.e. $\min \{ d(u,q(u))+d(q(u),v), d(v,q(v))+d(q(v),u)\}$. The main idea is that now we only need to consider the difficult case, Case 2, if the relevant node is light, which allows us to implement the algorithm efficiently.

If the relevant node $w\in B(u)\cap B(v)$ in Case 2 is heavy, we obtain a 2-approximation through the closest heavy node: we get that $\min \{d(u,w),d(v,w)\} \le d(u,v)/2$, since d(u,v)=d(u,w)+d(w,v). W.l.o.g. say d(u,w)≤d(u,v)/2. Then $d(u,q(u))\le d(u,w)\le d(u,v)/2$, hence $d(u,q(u))+d(q(u),v)\le d(u,q(u))+d(q(u),u)+d(u,v) \le 2d(u,v)$.

**Maintaining estimates through heavy nodes.** For the stretch analysis above, we need to maintain a shortest path tree from each heavy node. We ensure a bunch grows at most $O(\log _{1+\epsilon }(nW))$ times. Hence by a total load argument we get that the *total* number of nodes in all bunches over all updates is at most $O(\tfrac{n}{p}\cdot \log _{1+\epsilon }(nW))$. This implies there cannot be too many heavy nodes in total, hence we can enforce that $V_\mathrm{{heavy}}$ only grows by keeping in $V_\mathrm{{heavy}}$ all nodes that were once heavy (for details see Lemma 5.1). As a consequence, we can maintain multi-source approximate distances from all heavy nodes efficiently: we can compute shortest paths from this set to all other nodes in $O(|V_\mathrm{{heavy}}|m)=\tilde{O}(\tfrac{nm}{p\tau })$ time.

**Running time of the algorithm with bunch overlap thresholds.** Summarizing, we compute bunches and clusters in *O*(*m*/*p*) time, compute distances from *A* to *V* in $O(|A|m)=\tilde{O}(pnm)$ time, and we can compute shortest paths from the heavy nodes in $O(|V_\mathrm{{heavy}}|m)=O(\tfrac{nm}{p\tau })$ time.

Finally, we consider Case 2. For each node u∈V, we consider every light node w∈B(u), and then all nodes v∈C(w). Since *w* is light, we have $|C(w)|\le \tau$, so this takes time $\tilde{O}(n\cdot \tfrac{1}{p}\cdot \tau )$.

Hence, in the total update time, we obtain $\tilde{O}((pnm+\tfrac{nm}{p\tau }+n\tau /p)\log ^2(nW))$. Setting $p=m^{-1/4}$ and $\tau = m^{1/2}$ gives a total update time of $\tilde{O}(nm^{3/4}\log (nW))$. We provide the details in Sect. 5.1.

Again, for a decremental algorithm, we need to combine this new idea with the subtleties we had in the previous section: the bunches, their overlaps, and the heavy nodes keep on changing over the updates. We obtain a total update time that matches the stated static running time (up to subpolynomial factors) for the algorithm using bunch overlap thresholds.

#### (2+ϵ)-APSP for Unweighted Graphs

We observe that in unweighted graphs mixed approximation can always be turned into a multiplicative approximation – at the cost of a blow-up in the number of vertices. More precisely, we prove the following reduction.

##### Theorem 3.1

Let $$\mathcal {A}$$ be an algorithm that provides a (a+ϵ,k)-approximation for APSP in $$\tau _{\mathcal {A}}(n',m')$$ time, for any unweighted $n^{\prime }$-node $m^{\prime }$-edge graph $G^{\prime }$, and any constants $$a,k\in \mathbb {N}_{\ge 1}$$ and $$\epsilon \ge 0$$. Given an unweighted graph G=(V,E) on *n* nodes and *m* vertices, we can compute (a+(k+2)ϵ,0)-APSP in $$\tau _{\mathcal {A}}(n+km,(k+1)m)$$ time.

If $\mathcal {A}$ is a dynamic algorithm, then each update takes $(k+1)\cdot [\text {update time of }\mathcal {A}]$ time. The query time remains the same (up to a constant factor).

This reductions allows for ϵ=0 as well. However, for our applications the update time of $\mathcal {A}$ will depend on 1/ϵ, so we only apply this reduction for ϵ>0. Theorem 1.3 is obtained by splitting every edge into *k* edges, by introducing new nodes on the edge. Now a path corresponding to a +k approximation cannot take a non-trivial detour. We combine this result with Theorem 1.2 to obtain a (2+ϵ)-approximation for unweighted graphs in $\tilde{O}(m^{7/4})$ total update time. For details, see Sect. 5.2.

### (1+ϵ,2(k-1))-APSP for Unweighted Graphs

In this section, we describe our near-additive APSP algorithm. Our work is inspired by a classic result of Dor, Halperin, and Zwick [24], that presented a static algorithm that computes purely additive +2(k-1) approximation for APSP in $\tilde{O}(n^{2-1/k}m^{1/k})$ time in unweighted graphs. Our goal is to obtain similar results dynamically. More concretely, we obtain decremental (1+ϵ,2(k-1))-approximate APSP in $O(n^{2-1/k+o(1)}m^{1/k})$ total update time in unweighted graphs.

#### (1+ϵ,2)-APSP

To explain the high-level idea of the algorithm, we first focus on the special case that k=2, and that we only want to approximate the distances between pairs of nodes at distance at most *d* from each other, for a parameter *d*. In this case, we obtain a +2-additive approximation in $\tilde{O}(n^{3/2}m^{1/2} d )$ time. This case already allows to present many of the high-level ideas of the algorithm. Later we explain how to extend the results to the more general case. We start by describing the static algorithm from [24], and then explain the dynamic version. At a high level, we compute exact distances v×V for some nodes $v\in A_1\subseteq V$, and then use this to obtain approximate distances v×V for all other nodes $v\in V\setminus A_1$. The static version of the data structure is as follows. See also the pseudocode in Algorithm 1.

- Let $s_1= (\frac{m}{n})^{1/2}$. Let $V_1$ be the set of *heavy nodes*: $V_1:= \{ v \in V: \deg (v) \ge s_1\}$. Also let $E_2$ be the set of *light edges*, i.e. edges with at least one endpoint with degree less than $s_1$.
- **Node set** $A_1$. Construct a hitting set $A_1$ of neighborhoods of nodes in $V_1$. This means that every node $v \in V_1$ has a neighbor in $A_1$. The size of $A_1$ is $O(n \log {n}/s_1)$.
- **Edge set** $E^*$. Let $E^*$ be a set of size *O*(*n*) such that for each $v \in V_1$, there exists $u \in A_1 \cap N(v)$ such that $\{u,v\} \in E^*$.
- **Computing distances from** $A_1$. Store distances $A_1 \times V$ by running a BFS from each $u \in A_1$ on the input graph *G*.
- **Computing distances from** $V \setminus A_1$. For each $u \in V \setminus A_1$ store a shortest path tree, denoted by $T_u$ rooted at *u* by running Dijkstra on $(V, E_2 \cup E^* \cup E^{A_1}_u)$, where $E^{A_1}_u$ is the set of weighted edges corresponding to distances in $(\{u\} \times A_1)$ computed in the previous step.

**Figure:**
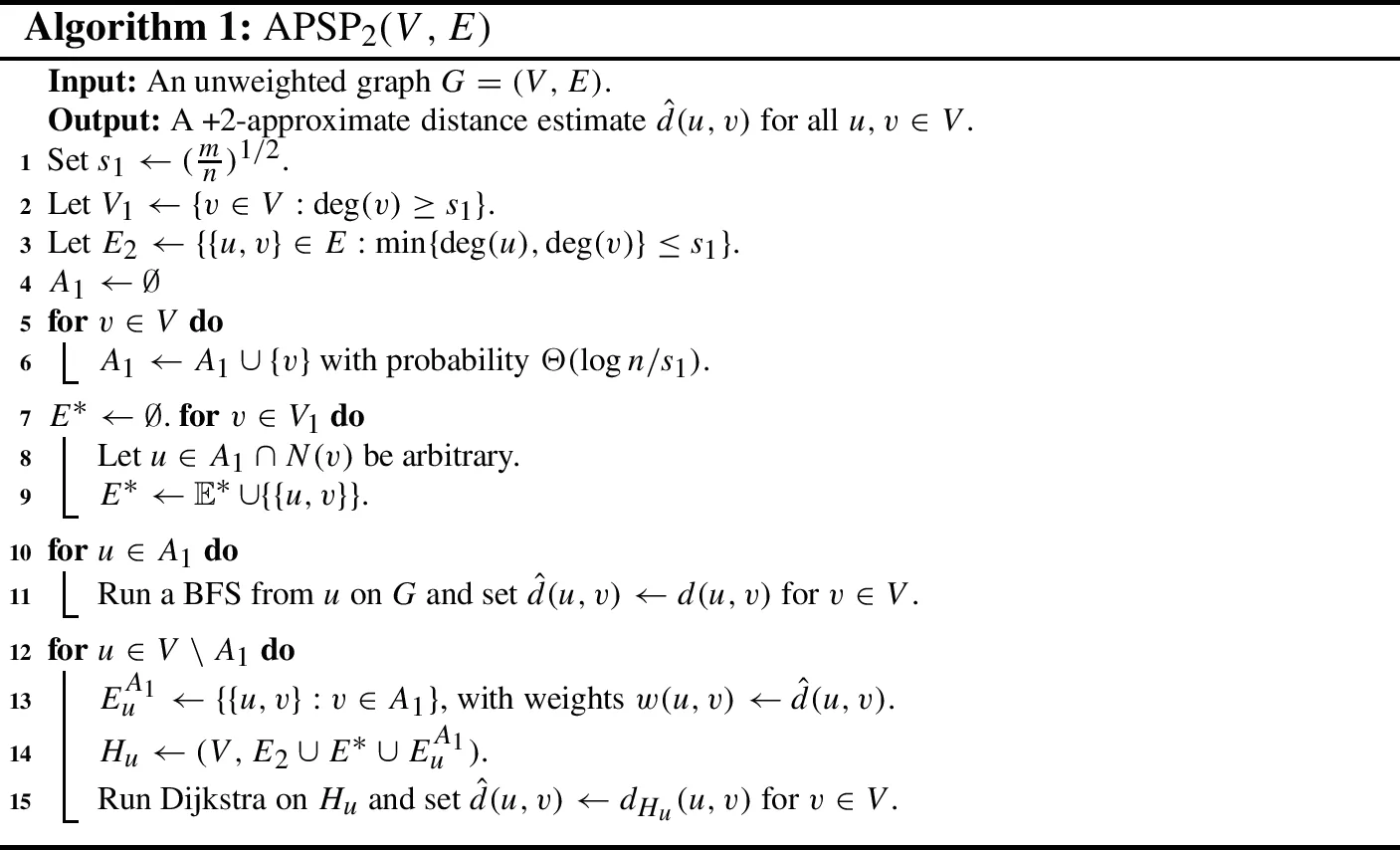

**Dynamic data structure.** The static algorithm computes distances in two steps. First, it computes distances from $A_1$ by computing BFS trees in the graph *G*. This step can be maintained dynamically by using *Even-Shiloach trees (ES-trees)* [74], a data structure that maintains distances in a decremental graph. Maintaining the distances up to distance *O*(*d*) takes $O(|A_1|md)$ time. The more challenging part is computing distances from the remaining nodes $V \setminus A_1$. Here the static algorithm computes distances in the graphs $H_u=(V, E_2 \cup E^* \cup E^{A_1}_u)$. Note that the graphs $H_u$ change dynamically in several ways. First, in the static algorithm, the set $E_2$ is the set of light edges. To keep the correctness of the dynamic algorithm, every time that a node *v* no longer has a neighbor in $A_1$, we should add all its adjacent edges to $E_2$. Second, when an edge in $E^*$ is deleted from *G*, we should replace it by an alternative edge if such an edge exists. Finally, the weights of the edges in $E^{A_1}_u$ can change over time, when the estimates change. This means that other than deletions of edges, we can also add new edges to the graphs $H_u$ or change the weights of their edges. Because of these edge insertions we can no longer use the standard ES-tree data structure to maintain the distances, because that only works in a decremental setting.

To overcome this, we use *monotone ES-trees*, a generalization of ES-trees proposed by [2, 7]. In this data structure, to keep the algorithm efficient, when new edges are inserted, the distance estimates do not change. In particular, when edges are inserted, some distances may decrease, however, in such cases the data structure keeps an old larger estimate of the distance. The main challenge in using this data structure is to show that the stretch analysis still holds. In particular, in our case, the stretch analysis of the static algorithm was based on the fact that distances from the nodes $V \setminus A_1$ are computed in the graphs $H_u$, where in the dynamic setting, we do not have this guarantee anymore.

**Stretch analysis.** The high-level idea of the stretch analysis uses the special structure of the graphs $H_u$. To prove that the distance estimate between *u* and *v* is at most d(u,v)+2, we distinguish between two cases: 1) $N(v)\cap A_1\ne \emptyset$; and 2) $N(v)\cap A_1=\emptyset$.

In the first case, an edge of the form $\{v,x\}$ is in $E^*$ for some $x \in A_1 \cap N(v)$, and since we maintain correctly the distances from $A_1$, we can prove that we get an additive stretch of at most 2 in this case.

In the second case, all the edges adjacent to *v* are in $E_2 \subseteq H_u$, and here we can use an inductive argument on the length of the path to prove that we get an additive stretch of at most 2. Note that the estimate that we get can be larger compared to the distance between *u* and *v* in the graph $H_u$, but we can still show that the additive approximation is at most 2 as needed. For a detailed stretch analysis, see Sect. 6.1.2.

**Update time.** Our update time depends on the size of the graphs $H_u$. While in the static setting it is easy to bound the number of edges in $E_2$ and $E^*$, in the decremental setting, as new edges are added to these sets, we need a more careful analysis. For example, in the static setting $|E^*|=O(n)$, as any node only adds one adjacent edge to the set $E^*$, where in the decremental setting, we may need to add many different edges to this set because the previous ones got deleted. However, we can still prove that even if a node needs to add to $E^*$ edges to all its adjacent neighbors in $A_1$ during the algorithm, the size of $H_u$ is small enough as needed. A detailed analysis of the update time appears in Sect. 6.1.3, where we show that the total update time is $\tilde{O}(m^{1/2}n^{3/2}d)$ in expectation, this matches the static complexity up to the factor *d*.

**Handling large distances.** The algorithm described above is efficient if *d* is small, to obtain an efficient algorithm for the general case, we combine our approach with a decremental near-additive APSP algorithm. More concretely, we use an algorithm from Henzinger et al. [7] that computes $(1+\epsilon ,\beta )$-approximate APSP in a total update time that depends on β. For $\beta =n^{o(1)}$, their total update time is bounded by $O(n^{2+o(1)})$. Now we set $d=\Theta (\beta /\epsilon )$, and distinguish between two cases. For pairs of nodes at distance at most *d* from each other, we get a +2-additive approximation as discussed above, in $O(m^{1/2}n^{3/2+o(1)})$ time. For pairs of nodes at distance larger than *d*, the near-additive approximation is already a (1+O(ϵ))-approximation by the choice of the parameter *d*. For such pairs, the additive β term becomes negligible, as their distance is $\Omega (\beta /\epsilon )$. Overall, we get a (1+ϵ,2)-approximation for APSP in $O(m^{1/2}n^{3/2+o(1)})$ time. In fact, if the graph is dense enough ($m=n^{1+\Omega (1)}$), the $n^{o(1)}$ term is replaced by a poly-logarithmic term. For more details see Sect. 6.2.4.

#### General *k*

We can extend the algorithm to obtain a (1+ϵ,2(k-1))-approximate APSP in $O(n^{2-1/k+o(1)}m^{1/k})$ total update time, by adapting the general algorithm of [24] to the dynamic setting. At a high-level, instead of having one hitting set $A_1$, we have a series of hitting sets $A_1,A_2,...,A_k$, such that the set $A_i$ hits nodes of degree $s_i$. We compute distances from $A_i$ in appropriate graphs $H_u^i$, that are sparser when the set $A_i$ is larger. Balancing the parameters of the algorithm leads to the desired total update time. For full details and proofs see Sect. 6.2.

#### Limitations of previous approaches

We next explain why approaches used in previous decremental algorithms cannot be generalized to obtain our near-additive results. First, the unweighted (1+ϵ,2)-approximate APSP algorithm that takes $$\tilde{O}(n^{5/2}/\epsilon )$$ time [2], is inspired by static algorithms for +2-additive emulators, sparse graphs that preserve the distances up to a +2-additive stretch. The main idea is to maintain a sparse emulator of size $$m' =\tilde{O}(n^{3/2})$$, and exploit its sparsity to obtain a fast algorithm taking $$\tilde{O}(m'n)= \tilde{O}(n^{5/2}/\epsilon )$$ time. This construction however is specific for the +2 case, and cannot be generalized to a general *k*. Note that the update time of the algorithm crucially depends on the size of the emulator, and to obtain a better algorithm for a general *k*, we need to be able to construct a sparser emulator for a general *k*. However, a beautiful lower bound result by Abboud and Bodwin [83] shows that for any constant *k* and δ>0, a purely additive +k emulator should have $$\Omega (n^{4/3-\delta })$$ edges. This implies that dynamic algorithms that are based on purely additive emulators with $m^{\prime }$ edges seem to require $$\Theta (n\cdot m')=\Omega (n^{7/3-\delta })$$ time, and we cannot get running time arbitrarily close to $$O(n^2)$$. An alternative approach is to build a *near-additive* emulator. This is done in [7], where the authors show a $$(1+\epsilon , 2(1+2/\epsilon )^{k-2})$$-approximation algorithm for decremental APSP with expected total update time of $$\tilde{O}(n^{2+1/k} (37/\epsilon )^{k-1})$$. Here the running time indeed gets closer to $$O(n^2)$$, but this comes at a price of a much worse additive term of $$2(1+2/\epsilon )^{k-2}$$. While in the static setting there are also other bounds obtained for near-additive emulators, such as $$O(1+\epsilon ,O(k/\epsilon )^{k-1})$$ emulators of size $$O(kn^{1+\frac{1}{2^{k+1}-1}})$$ [84–87], in all of the constructions the additive term depends on ϵ, and the dependence on ϵ is known to be nearly tight for small *k* by lower bounds from [86]. Hence this approach cannot lead to small constant additive terms that do not depend on ϵ.

## (2+ϵ)-Approximate APSP for Weighted Graphs

Given an undirected, weighted graph G=(V,E,w), where *w* takes positive, integer values, our goal is to maintain (2+ϵ)-approximate all-pairs distances in *G*, denoted by $\hat{d}$. We provide an algorithm  with parameters $0< p,\epsilon \le 1$, and $0< \rho \le 1$ that gives a (2+ϵ)-approximation of APSP for weighted graphs, with ratio between the maximum and minimum weight *W*. Overall, the total update time scales with $pnm + \tfrac{n^2}{p}$, for an exact statement see Lemma 4.7, which explicitly states the dependency on ρ, *W*, and 1/ϵ. The algorithm has a constant query time.

We recommend the reader to first read Sect. 3.1, where we provide a static version of the algorithm and we discuss the challenges and techniques of making this dynamic. This section starts with our high-level dynamic algorithm in Sect. 4.1, we provide further details and parametrized update time analysis of each step in Sect. 4.2, and we provide the stretch analysis in Sect. 4.3. In Sect. 4.4, we combine these and set the parameter *p* appropriately to obtain our final result.

### Decremental Data Structures

We will make use of the $(1+\epsilon _1)$-approximate^5^ bunches and clusters that are introduced in Sect. 2.2. However, for efficiency purposes, we do not use these bunches themselves, but instead look at bunches with some additional approximation. The goal is to ensure that the bunches do not increase too often.

Let *A* be a set of nodes sampled with probability p≥1/n. Recall that, for each node *v*, the *pivot* *p*(*v*) is the closest node in *A* to *v*.

Lemma 2.1 gives us an approximate pivot $\tilde{p}(v)$, together with a distance estimate $\delta _{\tilde{B}}$, such that $d_G(v,p(v)) \le \delta _{\tilde{B}}(v,\tilde{p}(v)) \le (1+\epsilon _1) d_G(v,p(v))$. Further, we have $(1+\epsilon _1)$-approximate bunches: $\tilde{B}(v):= \{w\in V: (1+\epsilon _1)d(v,w) < \delta _{\tilde{B}}(v,\tilde{p}(v))\}$, and distance estimate $\delta _{\tilde{B}}$ such that $d(u,v)\le \delta _{\tilde{B}}(u,v)\le (1+\epsilon _1)d(u,v)$ for all $u\in \tilde{B}(v)$.

We now define our final approximate bunch, which we denote by *B*(*v*).^6^

#### Definition 1

We need the following notions related to bunches and clusters:

- For all v∈V, we keep track of a $(1+\epsilon _2)$-approximate *bunch radius* *r*(*v*), which is a $(1+\epsilon _2)$-approximation of the approximate distance to the approximate pivot, i.e., of $\delta _{\tilde{B}}(v,\tilde{p}(v))$. We denote by $r^{(t)}$ the bunch radius at time *t*. Initially, we set $r^{(0)}(v):=\delta _{\tilde{B}}(v,\tilde{p}(v))$. We update $r^{(t)}(v)$ whenever $\delta _{\tilde{B}}(v,\tilde{p}(v)) > (1+\epsilon _2)r^{(t-1)}(v)$, if so then we set $r^{(t)}(v):=\delta _{\tilde{B}}(v,\tilde{p}(v))$.
- Initially, we set $B^{(0)}(v):= \tilde{B}^{(0)}(v)$. Whenever $r^{(t)}(v)\ne r^{(t-1)}(v)$, we set $B^{(t)}(v):=\tilde{B}^{(t)}(v)$, otherwise we set $B^{(t)}(v):=B^{(t-1)}(v)$.
- The *cluster* of a node u∈V is the set $C(u):= \{ v \in V: u \in B(v) \}$.

Note that these bunches *B*(*v*) increase at most $\log _{1+\epsilon _2}(nW)$ times by construction. Next, let us investigate the sizes of these sets.

#### Lemma 4.1

At any given time

(i) With high probability, *A* has size $\tilde{O} (p n)$;
(ii) With high probability, each bunch *B*(*v*) has size $\tilde{O} \left( \tfrac{\log (nW)}{p}\right)$;

#### Proof

(i) Since *A* is created by sampling each element of *V* with probability p≥1/n, we have $|A| \sim \text {Binomial}(n, p)$, so $\mathbb {E}[|A|] = pn$. By Chernoff bounds, for any δ>0:
  $$\begin{aligned} \Pr [|A| \ge (1+\delta )pn] \le e^{-\frac{\delta ^2 pn}{2+\delta }}. \end{aligned}$$
  Setting $\delta = 3c\log n$ gives $\Pr [|A| \ge (1+3c\log n)pn] \le e^{-pnc\log n}\le n^{-c}$, so with high probability |A|=O(pnlogn).
(ii) This holds trivially as $B(v)\subseteq \tilde{B}(v)$, and $|\tilde{B}(v)|=\tilde{O}\left( \tfrac{\log (nW)}{p}\right)$ at any moment in time due to Lemma 2.1.

□

Now, we give a description of the algorithm in Algorithm 2, which is a dynamic version of the static algorithm given in Sect. 3.1.1. Next, we give a proof of correctness and show how to implement each of the steps. We provide a separate initialization procedure, as for some steps it will turn out that the initialization step and the update steps are quite different.

Throughout the algorithm, we set $\tilde{\delta }:= (1+\epsilon _3)^{\lceil \log _{1+\epsilon _3}(\delta )\rceil }$ whenever we want a distance estimate δ to update at most $\log _{1+\epsilon _3}(nW)$ times. Note that we can always maintain such a $\tilde{\delta }$ with only a constant multiplicative factor in the total update time. Moreover, it is clear that $\delta \le \tilde{\delta } \le (1+\epsilon _3)\delta$. Below we define the functions Initialization(), and functions used after each edge update $\{u,v\}\in E$: Delete(*u*, *v*), Update(*u*, *v*, *w*(*u*, *v*)). Finally we have the Query() function that returns a final estimate over different data structures maintained.

**Algorithm 1:**
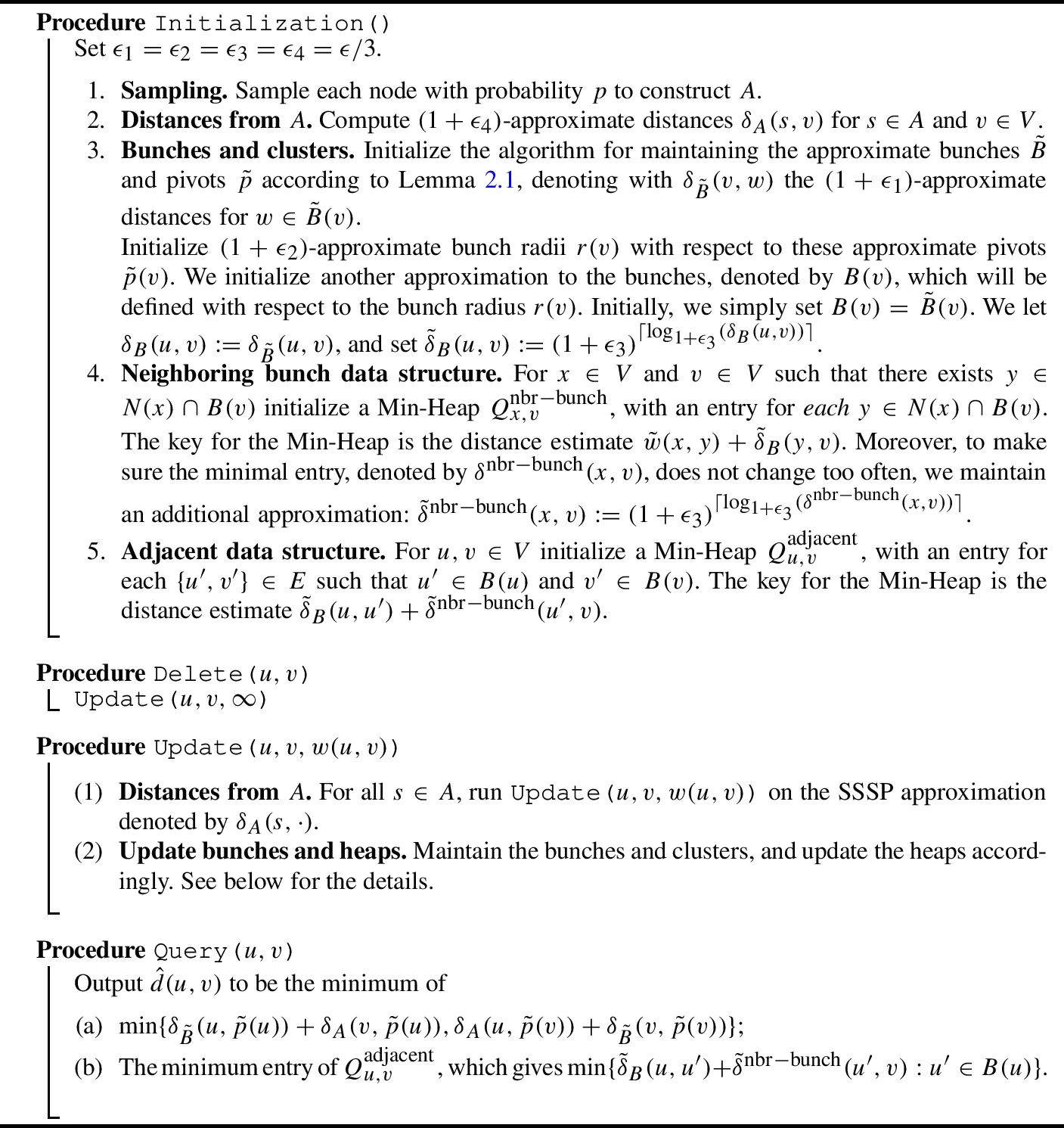
APSP$$_2$$(V,E,w,p,ϵ)

**Update bunches and heaps (Update Step (2)).** Now let us look more closely at how we maintain the bunches and clusters, and how these effect the heaps. We maintain the approximate bunches $\tilde{B}(v)$ for all v∈V by Lemma 2.1. From here maintain the additional approximate bunches *B*(*v*) for all v∈V as given in Definition 1, i.e., If there is a change to $\delta _{\tilde{B}}(v',\tilde{p}(v'))$, do the following two things:

- We check if $\delta _{\tilde{B}}^{(t)}(v',\tilde{p}^{(t)}(v'))> (1+\epsilon _2)r^{(t-1)}(v')$, and if so we set $r^{(t)}(v')=\delta _{\tilde{B}}^{(t)}(v',p^{(t)}(v'))$ and we set $B^{(t)}(v):=\tilde{B}^{(t)}(v)$. If not, we set $r^{(t)}(v')=r^{(t-1)}(v')$ and $B^{(t)}(v)=B^{(t-1)}(v)$.

Let $u^{\prime }\in V$ be a node for which there is a change to the bunch involving a node w∈V. Such a change can be: (i) *w* can leave the bunch of $u^{\prime }$ (ii) the distance estimate of *w* to $u^{\prime }$ can change while *w* stays within the bunch, or (iii) *w* can join the bunch of $u^{\prime }$. We formalize this as follows, using Δ to denote which case we are in.

(i) $$w\in B^{(t-1)}(u')$$ and $$w\notin B^{(t)}(u')$$, we set $$\Delta =\infty $$ to indicate that *w* is no longer in $B(u^{\prime })$.
(ii) $w\in B^{(t-1)}(u')$ and $w\in B^{(t)}(u')$ with an increased distance estimate $\tilde{\delta }_B^{(t)}(u',w)$, we set $\Delta =\tilde{\delta }_B^{(t)}(u',w)$ to be the new distance.
(iii) $w\notin B^{(t-1)}(u')$ and $w\in B^{(t)}(u')$, we set $\Delta =\tilde{\delta }_B^{(t)}(u',w)$ to be the distance.

Next, we do the following.

- Nbr-BunchChange($w,u^{\prime },\infty$) to update the corresponding entries $$\tilde{w}(v',w)+\tilde{\delta }_B(w,u')$$ in the data structures $$Q^\mathrm{{nbr-bunch}}_{v',u'}$$ for every $$v'\in \text {Set}^\mathrm{{nbr-bunch}}_{w,u'}$$. If this changes the minimal entry $$\delta ^\mathrm{{nbr-bunch}}(x,y)$$ in $$Q^\mathrm{{nbr-bunch}}_{x,y}$$ for some x,y∈V, we check whether we need to update $$\tilde{\delta }^\mathrm{{nbr-bunch}}(x,y)$$, if so we run the procedure
  Adj-MinHeapChange($$x,y,\tilde{\delta }^\mathrm{{nbr-bunch}}(x,y)$$).
- UpdateAdjacent($w,u^{\prime },\Delta$) to update the corresponding entries $$\tilde{\delta }_B(u',w)+\tilde{\delta }^\mathrm{{nbr-bunch}}(w,v')$$ in the data structures $$Q^\mathrm{{adjacent}}_{u',v'}$$ for every $$v'\in \text {Set}^\mathrm{{adj-bunch}}_{u',w}$$.

### Further Details and Time Analysis

In this section, we add further details of the procedures described and analyze the total update time. Considering the sampling of *A* in the algorithm, we note that *A* remains unchanged throughout the algorithm, hence it is only sampled once at the initialization. This takes *O*(*n*) time. To maintain distances from *A*, we use the decremental shortest path algorithm from Lemma 2.2 together with Lemma 4.1, which bounds the size of *A*, to obtain the following lemma.

#### Lemma 4.2

With high probability, we can maintain $(1+\epsilon _1)$-approximate SSSP from each node in *A*, providing us with $\delta _A(s,v)$ for s∈A and v∈V. We can do this in time $\tilde{O}(pnm/\epsilon _1)$ if $m=n^{1+\Omega (1)}$ and $pn=n^{\Omega (1)}$, and in time $\tilde{O}(pnm(1/\epsilon _1)^{\tilde{O}(\sqrt{\log n})})$ otherwise.

**Bunches and clusters.** We have approximate bunches $\tilde{B}(v)$ and pivots $\tilde{p}(v)$ by Lemma 2.1, now we maintain the bunch radii *r*(*v*) and approximate bunches *B*(*v*) and clusters *C*(*v*) according to Definition 1. As before, we simply set $\tilde{\delta }_B(u,v):= (1+\epsilon _3)^{\lceil \log _{1+\epsilon _3}(\delta _B(u,v))\rceil }$. It is immediate that these extra steps do not incur more than a constant factor in the update time with respect to Lemma 2.1, so we obtain the following result.

#### Lemma 4.3

We can maintain $(1+\epsilon _2)$-approximate bunch radii *r*(*v*) with respect to the approximate pivots $\tilde{p}$. With high probability, we can maintain approximate bunches *B*(*v*) and clusters *C*(*v*), and distance estimates $\delta _B(v,u)$ for u∈B(v), for all nodes v∈V. Moreover, we can maintain $\tilde{\delta }_B(u,v)$, a $(1+\epsilon _3)$-approximation of $\delta _B(u,v)$ that updates at most $O(\log _{1+\epsilon _3}(nW))$ times. This takes total update time $\tilde{O} (\tfrac{mn^\rho }{p}\log ^2(nW))\cdot O(\frac{1}{\rho \epsilon _2})^{1+2/\rho }$, for some arbitrarily small constant ρ, if $m=n^{1+\Omega (1)}$, and $\tilde{O}\left( \tfrac{m}{p}(1/\epsilon _2)^{\tilde{O}(\sqrt{\log n})}\right)$ otherwise.

**Neighboring bunch data structure.** To maintain $Q^\mathrm{{adjacent}}_{u,v}$, we maintain an auxiliary data structure, which is another Min-Heap $Q^\mathrm{{nbr-bunch}}_{x,v}$. This data structure consists of the following ingredients. We say *B*(*v*) is a *neighboring (approximate) bunch* of *x* if there exists an edge from *x* to *B*(*v*). For every node *x*, and every neighboring bunch *B*(*v*) our goal is to store all neighbors *y* such that $y \in B (v) \cap N(x)$ together with $\tilde{w} (x, y) + \tilde{\delta }_B(y, v)$, which allows us to find the neighbor *y* minimizing this sum for each neighboring bunch.^7^ Later, we will combine this with the distance estimate $\tilde{\delta }_B(u,x)$ for u∈B(x) to get a distance estimate from *u* to *v*, which will be stored in $Q^\mathrm{{adjacent}}_{u,v}$. To make sure we do not have to update the entries in $Q^\mathrm{{adjacent}}_{u,v}$, we do not maintain the minimum of $Q^\mathrm{{nbr-bunch}}_{x,v}$ exactly, but instead we maintain an additional approximation, denoted $\tilde{\delta }^\mathrm{{nbr-bunch}}(x,v)$ that only changes at most a polylogarithmic number of times.

#### Lemma 4.4

With high probability, we can maintain the data structures $Q^\mathrm{{nbr-bunch}}_{x,v}$ for all x,v∈V in total update time $\tilde{O} (\tfrac{n^2}{p}\cdot \log (nW)\cdot (\log _{1+\epsilon _3}(nW))+\log _{1+\epsilon _2}(nW)))$. Moreover, in the same total update time, we can maintain an approximation $\tilde{\delta }^\mathrm{{nbr-bunch}}(x,v)$ of the minimum entry of $Q^\mathrm{{nbr-bunch}}_{x,v}$ that only changes at most $\log _{1+\epsilon _2}(nW)\log _{1+\epsilon _3}(nW)$ times for each pair (*x*, *v*).

#### Proof

This data structure is initialized as follows:

- Iterate over every vertex v∈V, and over all y∈B(v). Then iterate over all neighbors x∈N(y) of *y* and add an entry to the data structure $Q^\mathrm{{nbr-bunch}}_{x,v}$ with key $\tilde{w}(x,y) + \tilde{\delta }_B(y,v)$. This takes $\tilde{O} (\tfrac{n^2}{p}\cdot \log (nW))$ initialization time, since there are *n* options for *v*, it has bunch size $\tilde{O}(\tfrac{\log (nW)}{p})$, so $\tilde{O}(\tfrac{\log (nW)}{p})$ options for *y*, which has at most *n* neighbors.

We need to facilitate two types of updates for an entry of $Q^\mathrm{{nbr-bunch}}_{x,v}$ corresponding to an edge $\{x,y\}$: increases in the weight $\tilde{w}(x,y)$ and increases in the distance estimates $\tilde{\delta }_B(y,v)$. We define the functions Nbr-EdgeChange(a)nd Nbr-BunchChange(r)espectively.

Next, we consider how to update this data structure. Suppose a value $\tilde{\delta }_B(y,v)$ changed for some y∈B(v) due to an update to the bunches (Lemma 4.3), then we would need to change the corresponding entries in $Q^\mathrm{{nbr-bunch}}_{x,v}$. Lemma 4.3 gives us *y*, *v*, and the new distance $\tilde{\delta }_B(y,v)$, which we need to update in all data structures $Q^\mathrm{{nbr-bunch}}_{x,v}$ for which $\{x,y\}\in E$. To do this efficiently, we maintain a set $\text {Set}_{x,y}^\mathrm{{nbr-bunch-edge}}$ for each edge $\{x,y\}\in E$ that stores for which v∈V there is an entry in $Q^\mathrm{{nbr-bunch}}_{x,v}$ and a pointer to where in that data structure it appears. We equip this set with the structure of a self-balancing binary search tree, so we have polylogarithmic insertion and removal times. We update this every time the appearance or location in $Q^\mathrm{{nbr-bunch}}_{x,v}$ changes. Now if $\tilde{w}(x,y)$ changes due to an update, we can efficiently change the corresponding places in $Q^\mathrm{{nbr-bunch}}_{x,v}$ for all relevant v∈V.

Moreover, for the changes in the distances from the bunches, we maintain a set $\text {Set}_{y,v}^\mathrm{{nbr-bunch}}$ for each $v\in V, y\in B(v)$ that stores for which x∈V there is an entry in $Q^\mathrm{{nbr-bunch}}_{x,v}$ and a pointer to where in that data structures it appears. We equip this set with the structure of a self-balancing binary search tree, so we have polylogarithmic insertion and removal times. We update this every time the appearance or location in $Q^\mathrm{{nbr-bunch}}_{x,v}$ changes. Now if $\tilde{\delta }_B(y,v)$ changes due to an update, we can efficiently change the corresponding places in $Q^\mathrm{{nbr-bunch}}_{x,v}$ for all relevant x∈V.

We define the function Nbr-EdgeChange(x,y,Δ), where $\{x,y\}\in E$, and Δ is the new value $\tilde{w}^{(t)}(x,y)$, or ∞ if the edge $\{x,y\}$ is deleted. Depending on Δ, we do one of the following:

(i) If $\Delta <\infty$, we update the corresponding estimates:
  For each entry $v\in \text {Set}_{x,y}^\mathrm{{nbr-bunch-edge}}$, update the corresponding entry $\tilde{w}(x,y)+\tilde{\delta }_B(y,v)$ in $Q^\mathrm{{nbr-bunch}}_{x,v}$ and update the entry in $\text {Set}_{x,y}^\mathrm{{nbr-bunch-edge}}$ accordingly.
(ii) If $\Delta =\infty$, we remove the corresponding estimates:
  For each entry $v\in \text {Set}_{x,y}^\mathrm{{nbr-bunch-edge}}$, remove the corresponding entry in $Q^\mathrm{{nbr-bunch}}_{x,v}$.

Concerning the total update time of Nbr-EdgeChange(,) we recall that $\tilde{w}(x,y) = (1+\epsilon _3)^{\lceil \log _{1+\epsilon _3}(w(x,y))\rceil }$. This means that each weight $\tilde{w}(x,y)$ gets updated at most $\log _{1+\epsilon _3}(W)$ times, giving total update time $\tilde{O}(\tfrac{n^2}{p}\cdot \log (nW)\cdot \log _{1+\epsilon _3}(W))$.

We define the function Nbr-BunchChange(y,v,Δ), where *y* is an element of $B^{(t-1)}(v)$ and/or $B^{(t)}(v)$, and Δ is the new value $\tilde{\delta }_B^{(t)}(y,v)$, or ∞ if *y* is no longer in *B*(*v*). Nbr-BunchChange(d)oes one of three things:

(i) If $\Delta <\infty$, $y\notin B^{(t-1)}(v)$, and $y\in B^{(t)}(v)$, we add the corresponding new estimates:
  We initialize $\text {Set}_{y,v}^\mathrm{{nbr-bunch}}$ to be empty.
  For all neighbors x∈N(y) of *y*, we add $\tilde{w}(x,y)+\tilde{\delta }_B(y,v)$ to $Q^\mathrm{{nbr-bunch}}_{x,v}$ and store *x* together with its location in $Q^\mathrm{{nbr-bunch}}_{x,v}$ in $\text {Set}_{y,v}^\mathrm{{nbr-bunch}}$.
(ii) If $\Delta <\infty$, $y\in B^{(t-1)}(v)$, and $y\in B^{(t)}(v)$, we update the corresponding estimates:
  For each entry $x\in \text {Set}_{y,v}^\mathrm{{nbr-bunch}}$, update the corresponding entry $\tilde{w}(x,y)+\tilde{\delta }_B(y,v)$ in $Q^\mathrm{{nbr-bunch}}_{x,v}$ and update the entry in $\text {Set}_{y,v}^\mathrm{{nbr-bunch}}$ accordingly.
(iii) If $\Delta =\infty$, $y\in B^{(t-1)}(v)$, and $y\notin B^{(t)}(v)$, we remove the corresponding estimates:
  For each entry $x\in \text {Set}_{y,v}^\mathrm{{nbr-bunch}}$, remove the corresponding entries from $Q^\mathrm{{nbr-bunch}}_{x,v}$ and from $\text {Set}_{y,v}^\mathrm{{nbr-bunch}}$.

Moreover, if one of these three actions change the minimum of $Q^\mathrm{{nbr-bunch}}_{x,v}$, this has an impact on the data structures $Q^\mathrm{{adjacent}}_{\cdot ,v}$. We run Adj-MinHeapChange(x,v,Δ), which is defined and analyzed in Lemma 4.5, to propagate this change. Here Δ is the minimum of $Q^\mathrm{{nbr-bunch}}_{x,v}$. The total update time of Adj-MinHeapChange(i)s included in Lemma 4.5, and not in this lemma.

As $\tilde{\delta }_B(y,v)$ can change at most $O(\log _{1+\epsilon _3}(nW)+\log _{1+\epsilon _2}(nW))$ times, and for each time we need at most $\tilde{O}(\deg (y))=O(n)$ time to update the data structure, we get total update time $\tilde{O}(\deg (y)(\log _{1+\epsilon _3}(nW))+\log _{1+\epsilon _2}(nW)))$ for the pair (*y*, *v*). Now note that there are at most $\tilde{O}(\tfrac{n}{p}\cdot \log (nW))$ such pairs, since there are *n* options for *v*, with $\tilde{O}(\tfrac{\log (nW)}{p})$ options for y∈B(v). So we get total update time $\tilde{O} (\tfrac{n^2}{p} \cdot \log (nW)\cdot (\log _{1+\epsilon _3}(nW))+\log _{1+\epsilon _2}(nW)))$.

Further, we maintain $\tilde{\delta }^\mathrm{{nbr-bunch}}(x,v)$, which is a $(1+\epsilon _3)$-approximation of the minimal entry $\delta ^\mathrm{{nbr-bunch}}(x,v)$ of $Q^\mathrm{{nbr-bunch}}_{x,v}$, defined by $\tilde{\delta }^\mathrm{{nbr-bunch}}(x,v):= (1+\epsilon _3)^{\lceil \log _{1+\epsilon _3}(\delta ^\mathrm{{nbr-bunch}}(x,v))\rceil }$. Note that $\tilde{\delta }^\mathrm{{nbr-bunch}}(x,v)$ is *not* monotone over time, so it is not trivial how often the estimate changes. However, note that $\tilde{\delta }^\mathrm{{nbr-bunch}}(x,v)$ increases at most $\log _{1+\epsilon _3}(nW)$ times in a row. It can only decrease $\log _{1+\epsilon _2}(nW)$ times, since the values $\tilde{w}(x,y)+\tilde{\delta }_B(y,v)$ are non-decreasing, except if new vertices $y\in B^{(t)}(v)$ are considered. This is only the case when the bunch $B^{(t)}(v)$ grows, which happens at most $\log _{1+\epsilon _2}(nW)$ times by Lemma 4.3. □

**Adjacent data structure.** Using $Q^\mathrm{{nbr-bunch}}_{x,v}$, we construct the required Min-Heap $Q^\mathrm{{adjacent}}_{u,v}$ for each pair u,v∈V. For every pair of nodes u,v∈V our goal is to store all edges $\{x, y\}$ such that x∈B(u) and y∈B(v), together with the distance $\tilde{\delta }_B(u,x)+\tilde{w}(x,y)+\tilde{\delta }_B(y,v)$.

#### Lemma 4.5

With high probability, we can maintain the data structures $Q^\mathrm{{adjacent}}_{u,v}$ for all u,v∈V in total update time $\tilde{O} (\tfrac{n^2}{p}\cdot \log (nW)\cdot \log _{1+\epsilon _3}(nW)\log _{1+\epsilon _2}(nW))$.

#### Proof

This data structure is initialized as follows:

- Iterate over all nodes *u*, all x∈B(u), and all neighboring bunches *B*(*v*) of *x* and add an entry for *x* to $Q^\mathrm{{adjacent}}_{u,v}$ with key $\tilde{\delta }_B(u,x)+\tilde{\delta }^\mathrm{{nbr-bunch}}(x,v)$. As there are at most *n* bunches in total, this takes $\tilde{O} (n \cdot \tfrac{\log (nW)}{p} \cdot n) = \tilde{O} (\tfrac{n^2}{ p}\cdot \log (nW))$ time.

Again, we maintain additional data structures to facilitate updates: for each x,v∈V, we maintain $\text {Set}_{x,v}^\mathrm{{adjacent}}$ which shows for which u∈V there is an entry in $Q^\mathrm{{adjacent}}_{u,v}$ and a pointer to where in that data structure it appears. We update this every time the appearance or location in $Q^\mathrm{{adjacent}}_{u,v}$ changes. Now if $\tilde{\delta }_B(u,w)$ changes due to an update, we can efficiently change the corresponding places in $Q^\mathrm{{adjacent}}_{u,v}$.

We define Adj-BunchChange(a)nalogously to Nbr-BunchChange(.) Adj-BunchChange(u,x,Δ) does one of the following three things:

(i) If $\Delta <\infty$, $x\notin B^{(t-1)}(u)$, and $x\in B^{(t)}(u)$, we add the corresponding new estimates:
  We initialize $\text {Set}_{u,x}^\mathrm{{adj-bunch}}$ to be empty.
  For all nodes with non-empty $\tilde{\delta }^\mathrm{{nbr-bunch}}(x,v)$, we add $\tilde{\delta }_B(u,x)+\tilde{\delta }^\mathrm{{nbr-bunch}}(x,v)$ to $Q^\mathrm{{adjacent}}_{u,v}$ and store *v* together with its location in $Q^\mathrm{{adjacent}}_{u,v}$ in $\text {Set}_{u,x}^\mathrm{{adj-bunch}}$.
(ii) If $\Delta <\infty$, $x\in B^{(t-1)}(u)$, and $x\in B^{(t)}(u)$, we update the corresponding estimates:
  For each entry $v\in \text {Set}_{u,x}^\mathrm{{adj-bunch}}$, update the corresponding entry $\tilde{\delta }_B(u,x)+\tilde{\delta }^\mathrm{{nbr-bunch}}(x,v)$ in $Q^\mathrm{{adjacent}}_{u,v}$ and update the entry in $\text {Set}_{u,x}^\mathrm{{adj-bunch}}$ accordingly.
(iii) If $\Delta =\infty$, $x\in B^{(t-1)}(u)$, and $x\notin B^{(t)}(u)$, we remove the corresponding estimates:
  For each entry $v\in \text {Set}_{u,x}^\mathrm{{adj-bunch}}$, remove the corresponding entries from $Q^\mathrm{{adjacent}}_{u,v}$ and from $\text {Set}_{u,x}^\mathrm{{adj-bunch}}$.

As updates in bunches/distance estimates in bunches happen $O(\log _{1+\epsilon _3}(nW)+\log _{1+\epsilon _2}(nW))$ times, see Definition 1, we get total update time $\tilde{O} (\tfrac{n^2}{p}\cdot \log (nW)\cdot (\log _{1+\epsilon _3}(nW)+\log _{1+\epsilon _2}(nW)))$.

We also define the function Adj-MinHeapChange(t)o process changes in $\tilde{\delta }^\mathrm{{nbr-bunch}}(\cdot ,\cdot )$. The function Adj-MinHeapChange(x,v,Δ) does one of the following three things:

(i) If $\Delta <\infty$ and the distance estimate from *x* to *v* is new,, we add the corresponding new estimates:
  We initialize $\text {Set}_{x,v}^\mathrm{{adj-heap}}$ to be empty.
  For all u∈C(x), we add $\tilde{\delta }_B(u,x)+\tilde{\delta }^\mathrm{{nbr-bunch}}(x,v)$ to $Q^\mathrm{{adjacent}}_{u,v}$ and store *u* together with its location in $Q^\mathrm{{adjacent}}_{x,v}$ in $\text {Set}_{x,v}^\mathrm{{adj-heap}}$.
(ii) If $\Delta <\infty$ and there already was a distance estimate from *x* to *v*,, we update the corresponding estimates:
  For each entry $x\in \text {Set}_{x,v}^\mathrm{{adj-heap}}$, update the corresponding entry $\tilde{\delta }_B(u,x)+\tilde{\delta }^\mathrm{{nbr-bunch}}(x,v)$ in $Q^\mathrm{{adjacent}}_{u,v}$ and update the entry in $\text {Set}_{x,v}^\mathrm{{adj-heap}}$ accordingly.
(iii) If $\Delta =\infty$ and there is no longer a distance estimate from *x* to *v*,, we remove the corresponding estimates:
  For each entry $x\in \text {Set}_{x,v}^\mathrm{{adj-heap}}$, remove the corresponding entries from $Q^\mathrm{{adjacent}}_{u,v}$ and from $\text {Set}_{x,v}^\mathrm{{adj-heap}}$.

Since each $\tilde{\delta }^\mathrm{{nbr-bunch}}(\cdot ,\cdot )$ updates at most $\log _{1+\epsilon _2}(nW)\log _{1+\epsilon _3}(nW)$ times by Lemma 4.4, we get total update time $\tilde{O}(\tfrac{n^2}{p}\cdot \log (nW)\cdot \log _{1+\epsilon _2}(nW)\cdot \log _{1+\epsilon _3}(nW))$ for this operation.

Together, Nbr-BunchChange(a)nd Adj-BunchChange(f)orm the function UpdateAdjacent(.) To be precise, we define UpdateAdjacent(a)s follows:

UpdateAdjacent(u,v,Δ):

1. Nbr-BunchChange(u,v,Δ)
2. Adj-BunchChange(u,v,Δ)

This means we obtain total update time

$$\begin{aligned}&\tilde{O}(\tfrac{n^2}{p}\cdot \log (nW)\cdot (\log _{1+\epsilon _3}(nW)+\log _{1+\epsilon _2}(nW)+\log _{1+\epsilon _2}(nW)\cdot \log _{1+\epsilon _3}(nW)))\\ =&\tilde{O}(\tfrac{n^2}{p}\cdot \log (nW) \cdot \log _{1+\epsilon _3}(nW)\cdot \log _{1+\epsilon _2}(nW)). \end{aligned}$$

□

**Query time.** Finally, we observe that the query is relatively straightforward and can be done in constant time.

#### Lemma 4.6

 has constant query time.

#### Proof

A query $\texttt {Query}(u,v)$ consists of the minimum over four values. The first two can be computed in constant time, as the components are all maintained explicitly. The last two are maintained explicitly. This gives us constant query time in total. □

We summarize this section in the following lemma.

#### Lemma 4.7

With high probability, the algorithm  has total update time $\tilde{O} ((pnm + \tfrac{n^2}{ p})\log ^3(nW)(\tfrac{1}{\rho \varepsilon })^{O(1/\rho )})$ if $m=n^{1+\Omega (1)}$, $p=n^{\Omega (1)-1}$, and $m\le n^{2-\rho }$ for an arbitrarily small constant ρ. If $m\ge n^{2-\rho }$, we have total update time $\tilde{O} ((pnm + \tfrac{mn^ \rho +n^2}{ p})\log ^3(nW)(\tfrac{1}{\rho \varepsilon })^{O(1/\rho )})$. Otherwise we have total update time $\tilde{O} ((pnm + \tfrac{n^2}{ p})\log ^3(nW)(1/\varepsilon )^{\tilde{O}(\sqrt{\log n})})$.

#### Proof

We inspect the total update times from all components of Algorithm 2, using that $\varepsilon _1=\varepsilon _2=\varepsilon _3=\varepsilon _4=\varepsilon /3$. First of all, we can perform the sampling of *A* in *O*(*n*) time. By Lemma 4.2, we can maintain distances from *A* in $\tilde{O}(pnm/\varepsilon )$ total update time if $m=n^{1+\Omega (1)}$ *and* $pn=n^{\Omega (1)}$, or in $\tilde{O}\left( pnm(1/\varepsilon )^{\tilde{O}(\sqrt{\log n})}\right)$ total update time otherwise. By Lemma 4.3, we can maintain the bunches and clusters in $\tilde{O} (\tfrac{mn^\rho }{p}\cdot \log ^2(nW)))\cdot O(\tfrac{1}{\rho \varepsilon })^{1+2/\rho }$ total update time, for some arbitrarily small constant ρ, if $m=n^{1+\Omega (1)}$, or in $\tilde{O}(\tfrac{m}{p}\cdot \log ^2(nW))(1/\varepsilon )^{\tilde{O}(\sqrt{\log n})})$ total update time otherwise. Moreover, by Lemma 4.4, we can maintain our neighboring bunch data structure in $\tilde{O} (\tfrac{n^2}{p} \cdot \log (nW))\cdot (\log _{1+\epsilon _2}(nW)+\log _{1+\epsilon _3}(nW)))$ total update time. Finally, by Lemma 4.5, we can maintain our adjacent data structure in $\tilde{O} (\tfrac{n^2}{p} \cdot \log (nW))\cdot (\log _{1+\epsilon _2}(nW)\cdot \log _{1+\epsilon _3}(nW))$ total update time.

In total, this means for the case that $m=n^{1+\Omega (1)}$ and $p=n^{\Omega (1)-1}$, we have total update time

$$\begin{aligned}&\tilde{O} ((pnm + \tfrac{mn^{\rho }}{p} + \tfrac{n^2}{ p})\log (nW)\log _{1+\epsilon _2}(nW)\log _{1+\epsilon _3}(nW)(\tfrac{1}{\rho \varepsilon })^{1/\rho })\\ =&\tilde{O} ((pnm + \tfrac{mn^{\rho }}{p} + \tfrac{n^2}{ p})\log ^3(nW)(\tfrac{1}{\rho \varepsilon })^{O(1/\rho )}), \end{aligned}$$

If we furthermore assume that $n^\rho = O(n/p)$, i.e., $p = O(n^{1-\rho })$ for some arbitrarily small ρ, then we obtain the simplified version

$$\begin{aligned} \tilde{O} ((pnm + \tfrac{n^2}{ p})\log ^3(nW)(\tfrac{1}{\rho \varepsilon })^{O(1/\rho )}). \end{aligned}$$

For other choices of *m*, *n*,  and *p*, we obtain

$$\begin{aligned} \tilde{O} ((pnm + \tfrac{n^2}{ p})\log ^3(nW)(1/\varepsilon )^{\tilde{O}(\sqrt{\log n})}). \end{aligned}$$

□

### Stretch Analysis

We can now follow the same structure as outlined in the high-level overview to prove the stretch in different cases for each pair u,v∈V. In our analysis we also discuss how maintaining approximate bunches and distances impacts the final stretch.

#### Lemma 4.8

For all u,v∈V, the distance estimate $\hat{d}(u,v)$ returned by a query to  is a (2+ϵ)-approximation of the distance *d*(*u*, *v*).

#### Proof

Let u,v∈V. We will show that at any time *t*, if Query(*u*, *v*) returns $\hat{d}^{(t)}(u,v)$, then we have $d^{(t)}(u,v)\le \hat{d}^{(t)}(u,v)\le (2+\epsilon )d^{(t)}(u,v)$. Since we look at all data structures at the same time *t*, we omit the superscripts $\cdot ^{(t)}$ in the remainder of this proof.

First, let us note that $\hat{d}(u,v)$ consists of different distance estimates that all correspond to actual paths in *G*, hence we clearly have $d(u,v)\le \hat{d}(u,v)$. Heaps do not contain ‘old’ entries, entries that correspond to paths that are no longer in *G* at the time of query, since the update functions Nbr-BunchChange(,) Nbr-EdgeChange(,) Adj-MinHeapChange(,) and Adj-BunchChange(t)ake such entries out as soon as they are no longer relevant.

Next, we do a case distinction as follows. Let π denote the shortest path from *u* to *v*.

1. There exists $w\in \pi$ such that $w\notin (B(u)\cup B(v))$ (the left case in Fig. 1). Since w∉B(u), we have $d(u,w)\ge r(u) \ge \tfrac{\delta _{\tilde{B}}(u,\tilde{p}(u))}{1+\epsilon _2}$, so $\delta _{\tilde{B}}(u,\tilde{p}(u))\le (1+\epsilon _2)d(u,w)$. Similarly $\delta _{\tilde{B}}(v,\tilde{p}(v)) \le (1+\epsilon _2)d(w,v)$. Since d(u,w)+d(w,v)=d(u,v), we have either $d(u,w)\le \tfrac{d(u,v)}{2}$ or $d(v,w)\le \tfrac{d(u,v)}{2}$. Without loss of generality, assume $d(u,w) \le \tfrac{d(u,v)}{2}$, then by Query Step (a) in the algorithm we have:
  $$\begin{aligned} \hat{d}(u,v)&\le \delta _{\tilde{B}}(u,\tilde{p}(u))+\delta _A(v,\tilde{p}(u)) \\&\le \delta _{\tilde{B}}(u,\tilde{p}(u)) +(1+\epsilon _4)d(v,\tilde{p}(u))\\&\le \delta _{\tilde{B}}(u,\tilde{p}(u))+(1+\epsilon _4)(d(u,\tilde{p}(u))+d(u,v))\\&\le \delta _{\tilde{B}}(u,\tilde{p}(u))+(1+\epsilon _4)(\delta _{\tilde{B}}(u,\tilde{p}(u))+d(u,v))\\&\le (2+\epsilon _4)(1+\epsilon _2)d(u,w)+(1+\epsilon _4)d(u,v)\\&\le (2+\tfrac{3}{2}\epsilon _4+\tfrac{1}{2}\epsilon _4\epsilon _2+\epsilon _2)d(u,v)\\&\le (2+\epsilon )d(u,v), \end{aligned}$$
  where in the last step we use $\epsilon _4=\epsilon _2=\epsilon /3$.
2. There is no $$w\in \pi $$ such that $$w\notin (B(u)\cup B(v))$$ (the right case in Fig. 1). This means there has to be a direct edge from the cluster of *u* to the cluster of *v*: the shortest path π consists of a path from *u* to $u^{\prime }$, for some $u^{\prime }\in B\left(u\right)$, an edge $$\{u',v'\}\in E$$, for some $v^{\prime }\in B\left(v\right)$, and a path from $v^{\prime }$ to *v* (see the right case in Fig. 1). Note that in particular this includes the cases where u∈B(v) or v∈B(u), or more general when $$B(u)\cap B(v)\ne \emptyset $$, since we do not require $u\neq u^{\prime }$, $v\neq v^{\prime }$, $u^{\prime }\notin B\left(v\right)$, or $v^{\prime }\notin B\left(u\right)$. By Query Step (b) we have
  $$\begin{aligned} \hat{d}(u,v)&\le \tilde{\delta }_B(u,u')+\tilde{\delta }^{\mathrm{nbr-bunch}}(u',v)\\&\le (1+\epsilon _3)\delta _B(u,u')+(1+\epsilon _3)\delta ^{\mathrm{nbr-bunch}}(u',v)\\&\le (1+\epsilon _3)(1+\epsilon _1)d(u,u')+(1+\epsilon _3)(\tilde{w}(u',v')+\tilde{\delta }_B(v',v))\\&\le (1+\epsilon _3)(1+\epsilon _1)d(u,u')+(1+\epsilon _3)^2(w(u',v')+\delta _B(v',v))\\&\le (1+\epsilon _3)(1+\epsilon _1)d(u,u')+(1+\epsilon _3)^2(w(u',v')+(1+\epsilon _1)d(v',v))\\&\le (1+\epsilon _3)^2(1+\epsilon _1)d(u,v)\\&\le (2+\epsilon )d(u,v), \end{aligned}$$
  where in the last step we use $\epsilon _1=\epsilon _3=\epsilon /3$, and ϵ<1.

□

### Putting Everything Together

In this section we combine our lemmas in the following theorem. To obtain this result, we pick *p* to balance the different terms in the total update time.

**Theorem** 1.1*Given a graph* *G* *with positive, integer weights and a constant* 0<ϵ<1, *there is a decremental data structure that maintains a* (2+ϵ)*-approximation of APSP. The algorithm has constant query time and works against an oblivious adversary. The total update time is w.h.p.*

- $\tilde{O}(m^{1/2}n^{3/2}\log ^3(nW))$ *if* $m=n^{1+\Omega (1)}$ *and* $m\le n^{2-\rho }$ *for an arbitrary small constant* ρ,
- $\tilde{O}(m^{1/2}n^{3/2+\rho }\log ^3(nW))$ *if* $m\ge n^{2-\rho }$ *for any constant* ρ,
- $$\tilde{O}(m^{1/2}n^{3/2+o(1)}\log ^3(nW))$$ *otherwise,*

*where* *W* *is the ratio between the maximum and minimum weight.*

#### Proof

This follows from Lemma 4.7 and 4.8, by picking *p* to minimize $pnm + \tfrac{n^2}{ p}$. This gives $p=\sqrt{\tfrac{n}{m}}$, implying $pnm=\tfrac{n^2}{p}=m^{1/2}n^{3/2}$. Note that whenever $m=n^{1+\Omega (1)}$ and $m\le n^{2-\rho }$, this choice of *p* indeed satisfies the other requirements of Lemma 4.7: $p=n^{\Omega (1)-1}$. Since we now assume that ε and ρ are constants, the factor $(\tfrac{1}{\rho \varepsilon })^{O(1/\rho )}$ simplifies to *O*(1).

When $m=n^{1+o(1)}$, we obtain an extra factor $(1/\varepsilon )^{\tilde{O}(\sqrt{\log n})}$ in the total update time, which simplifies to $n^{o(1)}$ for constant ε. And when $m\ge n^{2-\rho }$, we obtain the extra factor $n^{\rho }$ and $(1/\varepsilon )^{\tilde{O}(\sqrt{\log n})}$, where the latter again simplifies to *O*(1) for constant ρ and ϵ. □

## $(2+\epsilon ,W_{u,v})$-APSP for Weighted Graphs and Unweighted 2-APSP

In this section, we show that an adapted version of the algorithm from Sect. 4 returns $(2+\epsilon ,W_{u,v})$-approximate distances with improved update time, where $W_{u,v}$ is the maximum weight on a shortest path from *u* to *v*. This result follows by the fact that in this case we do not need to handle the adjacent bunch case, but instead can consider an *overlap* bunch case (see the middle case in Fig. 2), which leads to an improved total update time.

To make this efficient, there are some additional complications. We introduce a parameter τ, the *bunch overlap threshold*, which bounds the cluster size. For nodes $w\in B(u)\cap B(v)$ with cluster smaller than τ, we can compute the estimate in the overlap case efficiently. If all nodes in the overlap B(u)∩B(v) have larger clusters, we obtain an estimate by computing shortest paths from the heavy nodes.

We recommend the reader to first read the static version of this algorithm in Sect. 3.2. We then present the details of the dynamic version in Sect. 5.1. Moreover, in Sect. 5.2, we show our general reduction from mixed APSP to purely multiplicative APSP for unweighted graphs. We use this reduction to obtain our unweighted (2+ϵ,0)-APSP result.

### $(2+\epsilon ,W_{u,v})$-APSP for Weighted Graphs

We use the same structure as in Sect. 4: we have bunches, and clusters exactly as before. We introduce the parameter $1\le \tau \le n$ to be set later. Now we define nodes to be heavy or light based on their cluster size.

- We call a node *u* *heavy* if it is or ever was contained in at least τ approximate bunches *B*(*v*), i.e., *u* is heavy at time *t* if $$|C^{(t')}(u)|\ge \tau $$ for some $t^{\prime }\leq t$. If a node is not heavy, we call it *light*.
- We denote $V_\mathrm{{heavy}}$ for the set of heavy nodes, and $V_\mathrm{{light}}=V\setminus V_\mathrm{{heavy}}$ for the light nodes. By definition of heavy nodes, a light node can become heavy, but a heavy node can never become light.
- Suppose we have a distance estimate $\delta _H(s,v)$ for all $s\in V_\mathrm{{heavy}},v\in V$. For each node u∈V, *q*(*u*) is the closest node in $V_\mathrm{{heavy}}$ to *u* according to the distance estimate $\delta _H$.

We can show that the size of $V_\mathrm{{heavy}}$ is bounded.

#### Lemma 5.1

At any given time, with high probability, $V_\mathrm{{heavy}}$ has size $\tilde{O} (\tfrac{n }{p\tau }\cdot \log (nW) \cdot \log _{1+\epsilon _3}(nW))$.

#### Proof

Let $B^{(t)}(v)$ and $C^{(t)}(w)$ denote the bunch of *v* and the cluster of *w* respectively at time *t*. Since bunch radius *r*(*v*) increases by at least a factor $1+\epsilon _3$ if the bunch changes, we have at most $\log _{1+\epsilon _3}(nW)$ different bunch radii. Until the bunch radius increases, the bunch can only lose vertices, which does not contribute to the total size. Since each bunch is of size $\tilde{O}(\tfrac{\log (nW)}{p})$ with high probability by Lemma 4.1, we obtain that the total size of a bunch over the course of the algorithm is bounded by $\left| \bigcup _{t} B^{(t)}(v)\right| =\tilde{O}\left( \tfrac{\log (nW)\cdot \log _{1+\epsilon _3}(nW)}{p}\right)$. Hence we have

$$\begin{aligned} |V_\mathrm{{heavy}}|\tau&\le \sum _{w\in V_\mathrm{{heavy}}} \tau \le \sum _{w\in V_\mathrm{{heavy}}} \left| \bigcup _t C^{(t)}(w) \right| \le \sum _{w\in V} \left| \bigcup _t C^{(t)}(w) \right| \\&= \sum _{v\in V} \left| \bigcup _t B^{(t)}(v) \right| = \tilde{O} ( \tfrac{n}{p}\cdot \log (nW) \cdot \log _{1+\epsilon _3}(nW)). \end{aligned}$$

Thus $$|V_\mathrm{{heavy}}| = \tilde{O} (\tfrac{n }{p\tau }\cdot \log (nW) \cdot \log _{1+\epsilon _3}(nW))$$. □

Now we are ready to present our new algorithm, see Algorithm 3.

**Algorithm 2:**
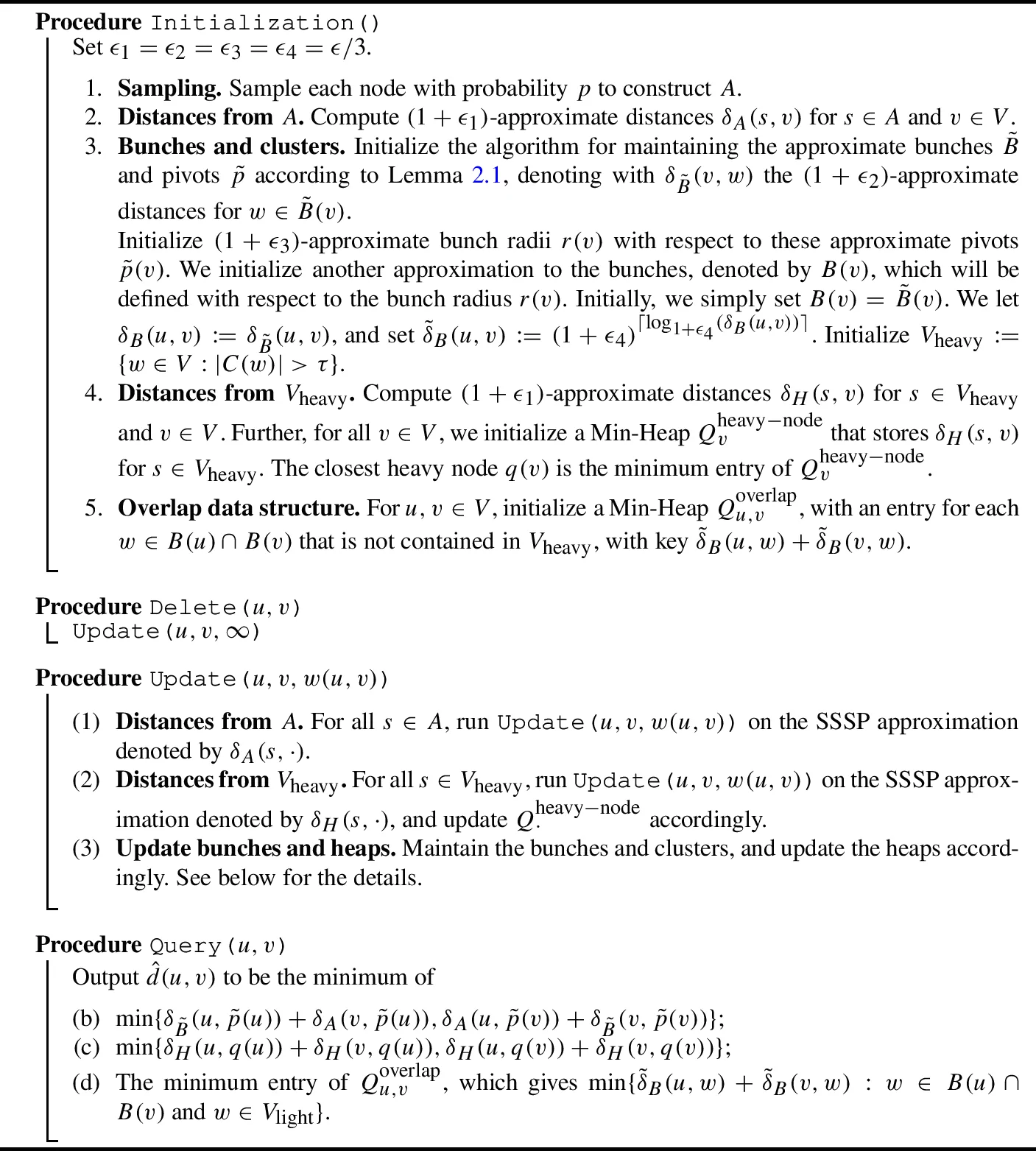
APSP$$_{2,W}$$($$V,E,w,p,\tau ,\epsilon $$)

**Updating bunches and heaps.** For completeness, we recall how we update the bunches and clusters. We now maintain different heaps. Again, we maintain the approximate bunches $\tilde{B}(v)$ for all v∈V by Lemma 2.1. From here maintain the additional approximate bunches *B*(*v*) for all v∈V as given in Definition 1, i.e., If there is a change to $\delta _{\tilde{B}}(v',\tilde{p}(v'))$, do the following two things:

- We check if $\delta _{\tilde{B}}^{(t)}(v',\tilde{p}^{(t)}(v'))> (1+\epsilon _3)r^{(t-1)}(v')$, and if so we set $r^{(t)}(v')=\delta _{\tilde{B}}^{(t)}(v',p^{(t)}(v'))$ and we set $B^{(t)}(v):=\tilde{B}^{(t)}(v)$. If not, we set $r^{(t)}(v')=r^{(t-1)}(v')$ and $B^{(t)}(v)=B^{(t-1)}(v)$.

Let $u^{\prime }\in V$ be a node for which there is a change to the bunch involving a node w∈V. Such a change can be: (i) *w* can leave the bunch of $u^{\prime }$ (ii) the distance estimate of *w* to $u^{\prime }$ can change while *w* stays within the bunch, or (iii) *w* can join the bunch of $u^{\prime }$. We formalize this as follows, using Δ to denote which case we are in.

(i) $$w\in B^{(t-1)}(u')$$ and $$w\notin B^{(t)}(u')$$, we set $$\Delta =\infty $$ to indicate that *w* is no longer in $B(u^{\prime })$.
(ii) $w\in B^{(t-1)}(u')$ and $w\in B^{(t)}(u')$ with an increased distance estimate $\tilde{\delta }_B^{(t)}(u',w)$, we set $\Delta =\tilde{\delta }_B^{(t)}(u',w)$ to be the new distance.
(iii) $w\notin B^{(t-1)}(u')$ and $w\in B^{(t)}(u')$, we set $\Delta =\tilde{\delta }_B^{(t)}(u',w)$ to be the distance.

Next, we do the following. If we are in Case (iii), we check the cluster size of *w*: if $$|C^{(t)}(w)|\ge \tau $$, then we add *w* to $$V_\mathrm{{heavy}}$$ and we initialize a SSSP approximation, denoted by $$\delta _H(w,\delta )$$. Moreover, for each $u^{\prime }$ in $$C^{(t-1)}(w)$$ we do the following:

- UpdateOverlap($w,u^{\prime },\infty$) to remove the corresponding entries $$\tilde{\delta }_B(u',w)+\tilde{\delta }_B(v',w)$$ from the data structures $$Q^\mathrm{{overlap}}_{u',v'}$$ for all $$v'\in \text {Set}^\mathrm{{overlap}}_{w,u'}$$. Here $$\text {Set}^\mathrm{{overlap}}_{w,u'}$$ stores exactly those $v^{\prime }$ such that there is an entry for *w* in $$Q^\mathrm{{overlap}}_{u',v'}$$. The details on $$\text {Set}^\mathrm{{overlap}}$$ can be found together with the definition and total update time of UpdateOverlap(a)re given in Lemma 5.3.

If we are in Case (iii) but $|C^{(t)}(w)|<\tau$, or we are in Case (i) or (ii), then we do the following

- UpdateOverlap($w,u^{\prime },\Delta$) to update the corresponding entries $$\tilde{\delta }_B(u',w)+\tilde{\delta }_B(v',w)$$ in the data structures $$Q^\mathrm{{overlap}}_{u',v'}$$ for all $$v'\in \text {Set}^\mathrm{{overlap}}_{w,u'}$$.

**Distances from** $V_\mathrm{{heavy}}$ **.** First, we note that a node is heavy if it is contained in at least τ bunches, or equivalently, if |C(w)|>τ. Hence by Lemma 4.3, we can easily keep track when nodes become heavy and should join $V_\mathrm{{heavy}}$.

Next, we use the shortest path algorithm from Lemma 2.2, which we combine with Lemma 5.1, which states that $V_\mathrm{{heavy}}$ might be growing over time, but its size is always bounded by $\tilde{O} (\tfrac{n }{p\tau }\cdot \log (nW) \cdot \log _{1+\epsilon _3}(nW))$. Moreover, we maintain the closest heavy nodes *q*(*v*) by maintaining a Min-Heap $Q^\mathrm{{heavy~pivot}}_v$ for each node *v*, with all nodes $u\in V_\mathrm{{heavy}}$ as entries with key $\delta _H(u,v)$. Throughout, we will use that we can extract the minimum entry of a Min-Heap in constant time, that we can insert entries in log(n) time, and that we can edit or delete entries in log(n) time, given that we know either the key or have a pointer to their location in the Min-Heap.

This clearly does not incur more than a constant factor in the update time, so together we obtain the following lemma.

#### Lemma 5.2

With high probability, we can maintain $(1+\epsilon _1)$-SSSP from each node in $V_\mathrm{{heavy}}$, providing us with $\delta _H(s,v)$ for $s\in V_\mathrm{{heavy}}$ and v∈V. Further, for all v∈V, we can maintain the closest heavy nodes *q*(*v*) if d(v,q(v))≤d. We can do this in total update time $\tilde{O} (\tfrac{m n }{p\tau \varepsilon _1}\cdot \log (nW) \cdot \log _{1+\epsilon _3}(nW))$ if $m=n^{1+\Omega (1)}$ and $\tfrac{n}{p\tau }=n^{\Omega (1)}$, and $\tilde{O} (\tfrac{m n }{p\tau }\cdot \log (nW) \cdot \log _{1+\epsilon _3}(nW)(1/\varepsilon _1)^{\tilde{O}(\sqrt{\log n})})$ otherwise.

**Overlap data structure.** Now we provide the details of how we maintain the data structure $Q^\mathrm{{overlap}}_{u,v}$ for all u,v∈V, which has entries for all nodes $w\in B(u)\cap B(v)\cap V_\mathrm{{light}}$.

#### Lemma 5.3

With high probability, we can maintain the data structures $Q^\mathrm{{overlap}}_{u,v}$ for all u,v∈V in total update time $\tilde{O} (\tfrac{n\tau }{p} \cdot \log (nW) \cdot \log _{1+\epsilon _3}(nW)\cdot \log _{1+\epsilon _4}(nW))$.

#### Proof

Conceptually, we initialize a Min-Heap $Q^\mathrm{{overlap}}_{u,v}$ for each pair $(u,v)\in V\times V$. In practice, we only create the Min-Heap if the first entry is about to be added. This avoids a $\Theta (n^2)$ initialization time. Now fix v∈V, and consider the moment we add a node *w* to *B*(*v*). For this data structure, we only need to consider $w\in V_\mathrm{{light}}$, hence *w* is part of at most τ bunches. When we add *w* to to *B*(*v*), we know it is part of at most τ other bunches *B*(*u*). For each such tuple (*u*, *w*, *v*), we add an entry to the Min-Heap $Q^\mathrm{{overlap}}_{u,v}$ for (*u*, *v*), which stores *w* with as key the distance estimate $\delta _w(u,v):=\tilde{\delta }_B(u,w)+\tilde{\delta }_B(w,v)$. For the total time, notice that for each v∈V, we have at most $\tilde{O}(\tfrac{\log (nW)}{p})$ different w∈B(v) for which we store at most τ distances, hence we have total initialization time $\tilde{O} (n \cdot \tfrac{\log (nW)}{p} \cdot \tau )$.

Next, we consider how to update this data structure. Suppose a value $\tilde{\delta }_B(u,w)$ changed for some w∈B(u) due to an update to the bunches (Lemma 4.3), then we would need to change the corresponding entries in $Q^\mathrm{{overlap}}_{u,v}$. Lemma 4.3 gives us *u*, *w*, and the new distance $\tilde{\delta }_B(u,w)$, which we need to update in all data structures $Q^\mathrm{{overlap}}_{u,v}$ for which $w\in B(u) \cap B(v)$. Clearly, checking all possible *v* and looking through the entire $Q^\mathrm{{overlap}}_{u,v}$ is too slow. Instead, we keep an additional data structure for each node w∈B(u): a set $\text {Set}_{w,u}^\mathrm{{overlap}}$ that shows for which v∈V it appears in $Q^\mathrm{{overlap}}_{u,v}$ and a pointer to where in $Q^\mathrm{{overlap}}_{u,v}$ it appears. We equip this set with the structure of a self-balancing binary search tree, so we have polylogarithmic insertion and removal times. We update this every time the appearance or location in $Q^\mathrm{{overlap}}_{u,v}$ changes. Now, if $\tilde{\delta }_B(u,w)$ changes due to an update, we can efficiently change the corresponding places in $Q^\mathrm{{overlap}}_{u,v}$ as follows.

We define the function UpdateOverlap(u,w,Δ), where *w* is an element of $B^{(t-1)}(u)$ and/or $B^{(t)}$, and Δ is the new value $\tilde{\delta }_B^{(t)}(u,w)$, or ∞ if *w* is no longer in $B^{(t)}(u)$. Note that we will only call this function for light *w*.^8^UpdateOverlap(d)oes one of three things:

(i) If $\Delta <\infty$, $w\notin B^{(t-1)}(u)$ and $w\in B^{(t)}(u)$. We add the new estimates:
  We initialize $\text {Set}_{w,u}^\mathrm{{overlap}}$ to be empty.
  For each v∈C(w), we add $\delta _w(u,v):= \tilde{\delta }_B(u,w)+\tilde{\delta }_B(w,v)$ to $Q^\mathrm{{overlap}}_{u,v}$ and store *v* together with its location in $Q^\mathrm{{overlap}}_{u,v}$ as entries in $\text {Set}_{w,u}^\mathrm{{overlap}}$ and $\text {Set}_{w,v}^\mathrm{{overlap}}$.
(ii) If $\Delta <\infty$, $w\in B^{(t-1)}(u)$ and $w\in B^{(t)}(u)$. We update the estimates:
  For each entry of *v* in $\text {Set}_{w,u}^\mathrm{{overlap}}$, update $\delta _w(u,v)= \tilde{\delta }_B(u,w)+\tilde{\delta }_B(w,v)$ in $Q^\mathrm{{overlap}}_{u,v}$ and update the entries in $\text {Set}_{w,u}^\mathrm{{overlap}}$ and $\text {Set}_{w,v}^\mathrm{{overlap}}$ accordingly.
(iii) If $\Delta =\infty$, then $w\in B^{(t-1)}(u)$ and $w\notin B^{(t)}(u)$. We remove the corresponding estimates:
  For each entry of *v* in $\text {Set}_{w,u}^\mathrm{{overlap}}$, remove $\delta _w(u,v)$ from $Q^\mathrm{{overlap}}_{u,v}$ and remove the corresponding entries from $\text {Set}_{w,u}^\mathrm{{overlap}}$ and $\text {Set}_{w,v}^\mathrm{{overlap}}$.

For the total update time, notice that in total at most $\tilde{O}(\tfrac{n}{p}\cdot \log (nW)\cdot \log _{1+\epsilon _3}(nW))$ nodes get added to a bunch, see Lemma 4.1. Since we only call this function when the added node *w* is light at that time, we have $|C(w)|\le \tau$ and thus step (i) takes $O(\tfrac{n\tau }{p}\cdot \log (nW)\cdot \log _{1+\epsilon _3}(nW))$ total update time. For step (ii), note that the distance can be updated at most $O(\log _{1+\epsilon _4}(nW))$ times and therefore this step has total update time $O(\tfrac{n\tau }{p}\cdot \log (nW)\cdot \log _{1+\epsilon _3}(nW)\log _{1+\epsilon _4}(nW))$. Further nodes get deleted from bunches at most as often as they get added, so the total update time of step (iii) is dominated by step (i). We obtain total update time $\tilde{O} ( \tfrac{n\tau }{p} \cdot \log (nW)\cdot \log _{1+\epsilon _3}(nW)\cdot \log _{1+\epsilon _4}(nW))$. □

We summarize the update times in the following lemma.

#### Lemma 5.4

With high probability, the algorithm  has total update time $\tilde{O} ((pnm + \tfrac{m n }{p\tau }+\tfrac{n\tau }{ p})\log ^2(nW)(1/\varepsilon )^{O(1)})$ if $m=n^{1+\Omega (1)}$, $p=n^{\Omega (1)-1}$, $\tfrac{n}{p\tau }=n^{\Omega (1)}$, and $\tau = O(n^{1-\rho })$. Otherwise we have total update time $\tilde{O} ((pnm +\tfrac{m n }{p\tau } +\tfrac{n\tau }{ p})\cdot \log ^3(nW)\cdot (1/\varepsilon )^{\tilde{O}(\sqrt{\log n})})$.

#### Proof

We inspect the total update times from all components of Algorithm 3. First of all, we can perform the sampling of *A* in *O*(*n*) time. By Lemma 4.2, we can maintain distances from *A* in $\tilde{O}(pnm/\varepsilon _1)$ total update time if $m=n^{1+\Omega (1)}$ *and* $pn=n^{\Omega (1)}$, or in $\tilde{O}(pnm(1/\varepsilon _1)^{\tilde{O}(\sqrt{\log n})})$ total update time otherwise. By Lemma 4.3, we can maintain the bunches and clusters in $\tilde{O} (\tfrac{mn^\rho }{p}\log ^2(nW))\cdot O(\frac{1}{\rho \epsilon _2})^{1+2/\rho }$ total update time, for some arbitrarily small constant ρ, if $m=n^{1+\Omega (1)}$, or in $\tilde{O}\left( \tfrac{m}{p}(1/\epsilon _2)^{\tilde{O}(\sqrt{\log n})}\right)$ total update time otherwise. Moreover, by Lemma 5.2, we can maintain distances from $V_\mathrm{{heavy}}$ in $\tilde{O} (\tfrac{m n }{p\tau \varepsilon _1}\cdot \log (nW)\cdot \log _{1+\epsilon _3}(nW))$ total update time if $m=n^{1+\Omega (1)}$ and $\tfrac{n}{p\tau }=n^{\Omega (1)}$, or in $\tilde{O} (\tfrac{m n }{p\tau }\cdot \log (nW)\cdot \log _{1+\epsilon _3}(nW)(1/\epsilon _1)^{\tilde{O}(\sqrt{\log n})})$ total update time otherwise. Finally, by Lemma 5.3, we can maintain our overlap data structure in $\tilde{O} (\tfrac{n\tau }{p} \cdot \log (nW)\cdot \log _{1+\epsilon _3}(nW)\cdot \log _{1+\epsilon _4}(nW))$ total update time.

In total, this means for the case that $m=n^{1+\Omega (1)}$, $p=n^{\Omega (1)-1}$, and $\tfrac{n}{p\tau }=n^{\Omega (1)}$, we have total update time

$$\begin{aligned} \tilde{O} \left( (pnm + \tfrac{mn^{\rho }}{p} + \tfrac{m n }{p\tau }+\tfrac{n\tau }{ p})\cdot \log ^3(nW)(1/\varepsilon )^{O(1)}\right) , \end{aligned}$$

using that ρ is a constant. If we furthermore assume that $n^\rho = O(n/\tau )$, i.e., $\tau = O(n^{1-\rho })$ for some arbitrarily small ρ, then we obtain the simplified version

$$\begin{aligned} \tilde{O} ((pnm + \tfrac{m n }{p\tau }+\tfrac{n\tau }{ p}\cdot \log ^3(nW)). \end{aligned}$$

For other choices of *m*, *n*, *p*,  and τ, we obtain

$$\begin{aligned} \tilde{O} ((pnm + \tfrac{m n }{p\tau }+\tfrac{n\tau }{ p})\cdot \log ^3(nW)\cdot (1/\epsilon )^{\tilde{O}(\sqrt{\log n})}). \end{aligned}$$

□

**Theorem** 1.2*Given a graph* *G* *with positive, integer weights and a constant* 0<ϵ<1, *there is a decremental data structure that maintains a* $(2+\epsilon ,W_{u,v})$*-approximation for APSP, where* $W_{u,v}$ *is the maximum weight on a current shortest path from* *u* *to* *v*. *The algorithm has constant query time and works against an oblivious adversary. The total update time is w.h.p.* $\tilde{O}( nm^{3/4}\log ^3(nW))$ *if* $m=n^{1+\Omega (1)}$, *and* $\tilde{O}( n^{1+o(1)}m^{3/4}\log ^3(nW))$ *otherwise, where* *W* *is the ratio between the maximum and minimum weight.*

#### Proof

This result follows from exchanging the adjacent data structure for the overlap data structure as described in this section.

By Lemma 5.4, we have a total update time scaling with $pnm+mn/(p\tau )+n\tau /p$. To balance out these terms, we set $mn/(p\tau )=n\tau /p$, hence $\tau =\sqrt{m}$. We set pnm=mn/(pτ), hence $p=m^{-1/4}$. Note that for $m=n^{1+o(1)}$, this choice for ρ and τ also satisfies the additional constraints of Lemma 5.4, so in this case we obtain total update time $\tilde{O}( (pnm+mn/(p\tau )+n\tau /p)\log ^3(nW)(1/\varepsilon )^{O(1)})= \tilde{O} (nm^{3/4}\log ^3(nW)(1/\varepsilon )^{O(1)})= \tilde{O} (nm^{3/4}\log ^3(nW))$, where the last equality holds for constant ε. If $m=n^{1+o(1)}$, we obtain $\tilde{O} (nm^{3/4}\log ^3(nW)(1/\epsilon )^{\tilde{O}(\sqrt{\log n})})=\tilde{O} (n^{1+o(1)}m^{3/4}\log ^3(nW))$, where again the equality holds for constant ε.

**Stretch analysis.** It remains to show that $\hat{d}(u,v)$ is a $(2+\epsilon ,W_{u,v})$-approximation of the distance *d*(*u*, *v*). We do a similar case distinction as in Lemma 4.8. Case 1 is exactly the same, since we maintain the relevant estimate through the pivots. For the remainder, we know there is no $w\in \pi$ such that $w\notin (B(u)\cup B(v))$, hence the two bunches are adjacent. We know create cases depending on whether the bunches overlap, and if so, if the node in the overlap is heavy or light.

**Fig. 3:**
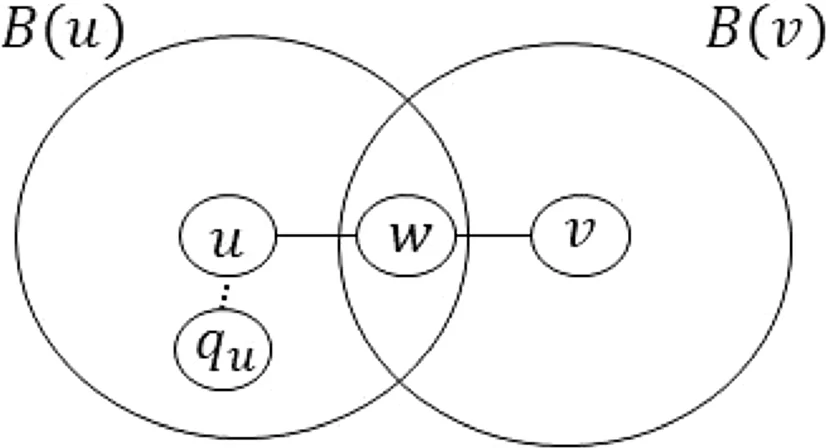
Possible scenario for the overlap between the bunches *B*(*u*) and *B*(*v*) and the shortest path π from *u* to *v* where the estimate can be obtained using the closest heavy node $q_u$ and $q_v$

Case 2: $$\pi \cap B(u)\cap B(v) =\emptyset $$ (the right case in Fig. 2). Hence π consists of a path from *u* to $u^{\prime }\in B\left(u\right)$, the edge $$\{u',v'\}$$ with $v^{\prime }\in B\left(v\right)$, and a path from $v^{\prime }$ to *v*. Since $v^{\prime }\notin B\left(u\right)$, we have $$d(u,v')\ge r(u) \ge \tfrac{\delta _{\tilde{B}}(u,\tilde{p}(u))}{1+\epsilon _3}$$, so $$\delta _{\tilde{B}}(u,\tilde{p}(u))\le (1+\epsilon _3)d(u,v')$$. Similarly, we have $$\delta _{\tilde{B}}(v,\tilde{p}(v))\le (1+\epsilon _3)d(u',v)$$. Combining this, we obtain $$\delta _{\tilde{B}}(u,\tilde{p}(u))+ \delta _{\tilde{B}}(v,\tilde{p}(v))\le (1+\epsilon _3)(d(u',v)+d(u,v')) = (1+\epsilon _3)(d(u,v)+w(u',v'))$$. Without loss of generality, assume that $$\delta _{\tilde{B}}(u,\tilde{p}(u))\le \tfrac{1+\epsilon _3}{2}(d(u,v)+w(u',v'))$$. By Query Step (a) we have:
  $$\begin{aligned} \hat{d}(u,v)&\le \delta _{\tilde{B}}(u,\tilde{p}(u))+\delta _A(v,\tilde{p}(u)) \\&\le \delta _{\tilde{B}}(u,\tilde{p}(u)) +(1+\epsilon _1)d(v,\tilde{p}(u))\\&\le \delta _{\tilde{B}}(u,\tilde{p}(u))+(1+\epsilon _1)(d(u,\tilde{p}(u))+d(u,v))\\&\le \delta _{\tilde{B}}(u,\tilde{p}(u))+(1+\epsilon _1)(\delta _{\tilde{B}}(u,\tilde{p}(u))+d(u,v))\\&\le (2+\epsilon _1)\tfrac{1+\epsilon _3}{2}(d(u,v)+w(u',v'))+(1+\epsilon _1)d(u,v)\\&\le (2+\tfrac{3}{2}\epsilon _1+\tfrac{1}{2}\epsilon _1\epsilon _3+\epsilon _3)d(u,v)+(1+\tfrac{1}{2}\epsilon _1+\tfrac{1}{2}\epsilon _1\epsilon _3+\epsilon _3)w(u',v') \\&\le (2+\epsilon /2)d(u,v)+(1+\epsilon /2)W_{u,v}\\&\le (2+\epsilon )d(u,v)+W_{u,v}. \end{aligned}$$
Case 3: There exists $w\in \pi$ such that $w\in B(u)\cap B(v)$ and $w\in V_\mathrm{{light}}$ (the middle case in Fig. 2). By Query Step (c), we have
  $$\begin{aligned} \hat{d}(u,v)&\le \tilde{\delta }_B(u,w)+\tilde{\delta }_B(v,w)\\&\le (1+\epsilon _4)(\delta _B(u,w)+\delta _B(v,w))\\&\le (1+\epsilon _4)(1+\epsilon _2)(d(u,w)+d(v,w))\\&= (1+\epsilon _2+\epsilon _2\epsilon _4+\epsilon _4)d(u,v)\\&\le (1+\epsilon )d(u,v). \end{aligned}$$
Case 4: There exists $w\in \pi$ such that $w\in B(u)\cap B(v)$ and $w\in V_\mathrm{{heavy}}$ (See Fig. 3). If w=q(u) then by Query Step (b), we have $\hat{d}(u,v)\le \delta _H(u,q(u))+\delta _H(v,q(u))\le (1+\epsilon _1)(d(u,q(u))+d(v,q(u))=(1+\epsilon _1)d(u,v)\le (1+\epsilon )d(u,v)$. If w=q(v), the argument is analogous. If $q(u)\ne w \ne q(v)$, then by definition of *q*(*u*) and *q*(*v*) we obtain $\delta _H(u,q(u))\le \delta _H(u,w)$ and $\delta _H(v,q(v))\le \delta _H(v,w)$. So we have $\delta _H(u,q(u))+\delta _H(v,q(v))\le (1+\epsilon _1)d(u,v)$, hence either $\delta _H(u,q(u))\le (1+\epsilon )\tfrac{d(u,v)}{2}$ or $\delta _H(v,q(v))\le (1+\epsilon _1)\tfrac{d(u,v)}{2}$. Without loss of generality, assume $\delta _H(u,q(u))\le (1+\epsilon _1) \tfrac{d(u,v)}{2}$. Combining this with the triangle inequality, we obtain
  $$\begin{aligned} \hat{d}(u,v)&\le \delta _H(u,q(u))+\delta _H(q(u),v)\\&\le \delta _H(u,q(u))+(1+\epsilon _1)d(q(u),v)\\&\le \delta _H(u,q(u))+(1+\epsilon _1)(d(u,q(u))+d(u,v))\\&\le (2+\epsilon _1)\delta _H(u,q(u))+(1+\epsilon _1)d(u,v)\\&\le (2+\tfrac{5}{2}\epsilon _1+\tfrac{1}{2}\epsilon _1^2)d(u,v)\\&\le (2+\epsilon )d(u,v). \end{aligned}$$

**Query time.** A query $\texttt {Query}(u,v)$ consists of the minimum over three values. The first two can be computed in constant time, as the components are all maintained explicitly. The last one is maintained explicitly. This gives us constant query time in total. □

In particular, this means we get (2+ϵ,1)-approximate APSP for unweighted graphs.

### Reduction and Unweighted (2+ϵ)-APSP

Next, we provide a reduction that transforms (a+ϵ,k)-APSP to (a+(k+2)ϵ,0)-APSP on unweighted graphs. This reductions allows for ϵ=0 as well. However, for our applications the update time of $\mathcal {A}$ will depend on 1/ϵ, so we only apply this reduction for ϵ>0.

Intuitively, we split every edge into k+1 edges to form $G^{\prime }$. Then we compute (2, *k*)-APSP on $G^{\prime }$, divide the result by k+1, and round down to the nearest integer. Because the additive error *k* is not enough to reach a new node in *G*, we can simply ignore this part of the path. In the following theorem we make this formal.

**Theorem** 3.1*Let* $$\mathcal {A}$$ *be an algorithm that provides a* (a+ϵ,k)-*approximation for APSP in* $$\tau _{\mathcal {A}}(n',m')$$ *time, for any unweighted* $n^{\prime }$-*node* $m^{\prime }$-*edge graph* $G^{\prime }$, *and any constants* $$a,k\in \mathbb {N}_{\ge 1}$$ *and* $$\epsilon \ge 0$$. *Given an unweighted graph* G=(V,E) on *n* *nodes and* *m* *vertices, we can compute* (a+(k+2)ϵ,0)-*APSP in* $$\tau _{\mathcal {A}}(n+km,(k+1)m)$$ *time.*

*If* $\mathcal {A}$ *is a dynamic algorithm, then each update takes* $(k+1)\cdot [\text {update time of }\mathcal {A}]$ *time. The query time remains the same (up to a constant factor).*

#### Proof

We construct the auxiliary graph $G^{\prime }=\left(V^{\prime },E^{\prime }\right)$ as follows.

$$\begin{aligned} V'&:= V \cup \{ \{u,v\}_i : \{u,v\}\in E, i\in \{1, 2, \dots , k\}\}; \\ E'&:= \bigcup _{\{u,v\}\in E} \{ \{u, \{u,v\}_1\}, \{\{u,v\}_1,\{u,v\}_2\}, \dots , \{\{u,v\}_{k-1},\{u,v\}_k\}, \{\{u,v\}_k, v\} \}. \end{aligned}$$

Clearly this graph has n+km vertices and (k+1)m edges. We compute (a+ϵ,k)-APSP on $G^{\prime }$ in $$\tau _{\mathcal {A}}(n+km,(k+1)m)$$ time using algorithm $$\mathcal {A}$$, and denote the output by $$\delta _{G'}$$. We set $$\delta (u,v):= \left\lfloor \tfrac{\delta _{G'}(u,v)}{k+1}\right\rfloor $$ for all u,v∈V.

We now show that δ gives (a+(k+2)ϵ,0)-approximate APSP on *G*. Let u,v∈V. We remark that $d_{G'}(u,v)=(k+1)d_G(u,v)$ for all u,v∈V. Which we will use twice.

First, we show that $d_G(u,v)\le \delta (u,v)$. This follows from $\delta (u,v) = \left\lfloor \tfrac{\delta _{G'}(u,v)}{k+1}\right\rfloor \ge \left\lfloor \tfrac{d_{G'}(u,v)}{k+1}\right\rfloor = \left\lfloor \tfrac{(k+1)d_G(u,v)}{k+1}\right\rfloor =d_G(u,v)$.

Second, we show that $\delta (u,v) \le (a+(k+2)\epsilon )\cdot d_G(u,v)$. This follows from

$$\begin{aligned} \begin{aligned}\delta (u,v)&= \left\lfloor \tfrac{\delta _{G'}(u,v)}{k+1}\right\rfloor \le \left\lfloor \tfrac{(a+\epsilon )\cdot d_{G'}(u,v)+k}{k+1}\right\rfloor = \left\lfloor \tfrac{(a+\epsilon )\cdot (k+1)d_{G}(u,v)+k}{k+1}\right\rfloor \\  &= \left\lfloor (a+\epsilon )\cdot d_G(u,v) +\tfrac{k}{k+1}\right\rfloor .\end{aligned} \end{aligned}$$

Now if ϵ=0, then $a\cdot d_G(u,v)$ is an integer, hence $\left\lfloor a\cdot d_G(u,v) +\tfrac{k}{k+1}\right\rfloor = a\cdot d_G(u,v)$. If ϵ>0, then $\epsilon d_G(u,v)+ \tfrac{k}{k+1}$ can be bigger than 1 – if it is not, we are done by the same argument. If $\epsilon d_G(u,v)+ \tfrac{k}{k+1}>1$, then

$$\begin{aligned} (k+1)\epsilon \cdot d_G(u,v) \ge (k+1)-k =1. \end{aligned}$$

Hence we obtain

$$\begin{aligned} \left\lfloor (a+\epsilon )\cdot d_G(u,v) +\tfrac{k}{k+1}\right\rfloor&\le (a+\epsilon )\cdot d_G(u,v) +1 \\&\le (a+\epsilon )\cdot d_G(u,v) + (k+1)\epsilon \cdot d_G(u,v)\\&= (a+(k+2)\cdot \epsilon )d_G(u,v). \end{aligned}$$

For the claim about dynamic algorithms, note that any update to an edge in *G*, corresponds to updating k+1 edges in $G^{\prime }$. The query consists of computing $$\delta (u,v) = \left\lfloor \tfrac{\delta _{G'}(u,v)}{k+1}\right\rfloor $$, which requires a query to the distance in $G^{\prime }$ plus constant time operations. □

In particular, this can give fast algorithms for sparse graphs. For the decremental case, we obtain the following result.

**Theorem** 1.3*Given an undirected unweighted graph* *G*, *there is a decremental data structure that maintains a* (2+ϵ)*-approximation for APSP with constant query time. The algorithm works against an oblivious adversary. W.h.p. the total update time is bounded by* $\tilde{O}( m^{7/4})$ *if* $m=n^{1+\Omega (1)}$, *and* $\tilde{O}( m^{7/4+o(1)})$ *otherwise.*

#### Proof

We apply the theorem above with the (2+ϵ,1)-APSP of this paper, Theorem 1.2. This takes $\tilde{O}( nm^{3/4})$ time if $m=n^{1+\Omega (1)}$, and $\tilde{O}( n^{1+o(1)}m^{3/4})$ time otherwise. □

## Approximate APSP with Additive Factors

In this section we give our last result, which is maintaining approximate APSP on unweighted graphs with a mixed additive and multiplicative stretch. First, we provide a decremental (1+ϵ,2)-APSP algorithm as a warm-up in Sect. 6.1. In Sect. 6.2, we generalize our techniques to provide an efficient decremental (1+ϵ,2(k-1))-APSP algorithm.

### Warm-up: (1+ϵ,2)-APSP

Our goal in this section is to use a simpler algorithm based on [24] that let us maintain (1+ϵ,2)-APSP in total update time of $O(m^{1/2}n^{3/2}O(1/\epsilon )^{O(\sqrt{\log {n}})})$ in unweighted undirected graphs with m=Ω(n) edges. Such an algorithm would outperform the approach in Sect. 4 when $m= \Omega ( n^{5/3+o(1)} )$, and it also gives a better *near-additive* approximation. We focus on obtaining an algorithm that can maintain +2-additive approximate distances for pairs within distance *d* in total update time $\tilde{O}(m^{1/2}n^{3/2}d)$. This approach can be combined with Theorem 2.6 to get our desired bounds.

#### Dynamic Data Structure

In Sect. 3.3, we described the static algorithm of [24]. We next describe the dynamic version of the algorithm. As mentioned above, we first focus on maintaining a data structure for pairs of nodes at distance at most *d* from each other. The algorithm is a variant of the static algorithm. At a high-level, we use ES-trees to maintain distances from nodes in $A_1$ in the graph *G*, giving us exact distances for these nodes. For the remaining nodes $u \in V \setminus A_1$, we use monotone ES-trees to maintain distances in the graph $H_u:=(V, E_2 \cup E^* \cup E^{A_1}_u)$, giving us approximate distances. The algorithm is summarized as follows.

1. **Node set** $A_1$ **.** We define a random set $A_1$ by sampling each node to $A_1$ with probability $\frac{c\ln {n}}{s_1}$ for $s_1= (\frac{m}{n})^{1/2}$ and a constant c≥2 of our choice.
2. **Edge set** $E^*$ **.** For each node *v* that has at least one neighbor in $A_1$, we add one edge $\{v,u\}$ to $E^*$, where *u* is an arbitrarily chosen neighbor of *v* in $A_1$.^9^
3. **Edge set** $E_2$ **.** For each node *v* that does not have a neighbor in $A_1$, we add all the adjacent edges to *v* to the set $E_2$.
4. **Maintaining ES-trees from nodes in** $A_1$ **.** For every $u \in A_1$, we maintain an ES-tree rooted at *u* up to distance d+2 in the graph *G*.
5. **Maintaining monotone ES-trees from nodes in** $V \setminus A_1$ **.** For each node $u \in V \setminus A_1$, we maintain a monotone ES-tree rooted at *u* up to distance d+2 in the graph $H_u = (V,E_2 \cup E^* \cup E_u^{A_1})$, where $E_u^{A_1}$ are all the edges of the form $\{u,v\}$ where $v \in A_1$. The weight of an edge $\{u,v\} \in E_u^{A_1}$ is $\ell _v(u)$, the level of *u* in the ES-tree rooted at *v* computed in the previous step.

**Maintaining the monotone ES-trees.** Next, we give more details about maintaining the monotone ES-trees from nodes $u \in V {\setminus } A_1$.

1. Initially, we construct a shortest path tree from $u \in V \setminus A_1$ in the original graph $H_u$, up to distance d+2.
2. Each time the distance estimate between *u* and a node $x \in A_1$ increases, we update the weight of the edge $\{u,x\} \in E^{A_1}_u$. Note that we can maintain these distances exactly (up to distance d+2) as we compute them in the ES-tree rooted at $x \in A_1$. We also update the monotone ES-tree rooted at *u* according to the weight change of $\{u,x\}$.
3. Each time an edge $\{v,x\} \in E^*$ between a node $v \in V {\setminus } A_1$ and $x \in A_1$ is deleted from the graph *G*, we distinguish between two cases. If *v* still has a neighbor $w \in A_1$, we first add a new edge $\{v,w\}$ to the set $E^*$ and then delete the edge $\{v,x\}$. Otherwise, we add to $E_2$ all the edges incident to *v* in the current graph *G*, and then delete the edge $\{v,x\}$.
4. If an edge $e \in E_2$ is deleted, we delete it from $H_u$.

There are several challenges in maintaining the monotone ES-trees. First, during the algorithm there are insertions to the graph $H_u$, as new edges are added to $E_2$ and $E^*$, and our goal is to show that our distance estimates still give a good approximation to the actual distances. In the static version of the algorithm, the algorithm computes the exact distances in the graphs $H_u$ and this provides a good approximation for the distances. However, in our case, the monotone ES-trees are not guaranteed to keep the actual distances in $H_u$ because they ignore distance decreases that come from edge insertions, and we should prove that this does not affect the stretch of the algorithm.

In addition, in the static version of the algorithm it is easy to argue that the graphs $H_u$ are sparse. For example, $E^*$ is a set of at most *O*(*n*) edges, since each node *v* added at most one edge. In our case, as $H_u$ changes dynamically, we need to prove that the total number of edges added to it is small. In particular, as each time that an edge in $\{v,x\} \in E^*$ gets deleted, we replace it by another edge, it could be the case that overall in the algorithm we add many different edges to $E^*$ which can affect the total update time of the algorithm. In addition, each time that a node *v* no longer has a neighbor in $A_1$, we add all its adjacent edges to $E_2$, and we should argue that the number of edges added to $E_2$ is small. We will discuss these issues in more detail later, and show that we can implement the monotone ES-trees efficiently. Next, we provide the stretch analysis of the algorithm.

#### Stretch Analysis

For any pair of nodes *u*, *v*, we denote by $\ell _u(v)$ the level of *v* in the ES-tree or monotone ES-tree rooted at *u*. We start with the following simple observation.

##### Claim 6.1

For any $u \in V, x \in A_1$ such that $d_G(u,x) \le d+2$, we have that $\ell _x(u)=\ell _u(x)=d_G(u,x).$

##### Proof

As $x \in A_1$, we maintain the exact distances from *x* up to distance d+2 in the ES-tree rooted at *x*. Hence, we have $\ell _x(u)=d_G(u,x)$. If $u \in A_1$, the same argument holds also for $\ell _u(x)$. If $u \in V \setminus A_1$, in Step 2 of the algorithm for maintaining the ES-tree we make sure that we also maintain the correct distance between *u* and *x* in the monotone ES-tree rooted at *u*, hence $\ell _u(x)=d_G(u,x)$. Note that as the edge $\{u,x\}$ always appears in $H_u$, and by part (4) of Lemma 2.4 we always maintain the exact distance estimate in this case. □

##### Claim 6.2

For any u,v∈V such that $d_G(u,v) \le d$, we have that $d_G(u,v) \le \ell _u(v) \le d_G(u,v) +2.$

##### Proof

If $v \in A_1$, this already follows from Claim 6.1. Hence we focus on the case that $v \in V {\setminus } A_1$. The left side of the inequality follows as $d_G(u,v) \le d_{H_u}(u,v) \le \ell _u(v)$, using part (2) of Lemma 2.4, and since the distances in $H_u$ can only increase compared to distances in *G*. This holds as the edges in $H_u$ are either edges in *G* or have a weight that is at least the weight of the shortest path between the corresponding endpoints in *G*. We next show that $\ell _u(v) \le d_G(u,v) +2$. We prove the claim by double-induction. First, on the time in the algorithm, and second on the distance $d_G(u,v)$.

**Base Case.** At the beginning of the algorithm, this follows from the correctness of the static algorithm, we give the details for completeness. Note that at the beginning of the algorithm, from part (1) of Lemma 2.4, we have that $\ell _u(v)=d_{H_u}(u,v)$ as long as $d_{H_u}(u,v) \le d+2$, and we show that this is indeed the case when $d_G(u,v) \le d$.

1. *v* has a neighbor in $A_1$. In this case there is an edge $\{v,x\} \in E^*$ such that $x \in A_1$. Note that as *x* is a neighbor of *v* it holds that $d_G(u,x) \le d+1$. As $x \in A_1$ and $d_G(u,x) \le d+1$, the graph $H_u$ has an edge $\{u,x\}$ with weight $d_G(u,x)$, and it also has the edge $\{v,x\} \in E^*$. Hence, at the beginning of the algorithm, we have
  $$\begin{aligned} \ell _u(v) = d_{H_u}(u,v) \le d_G(u,x) + d_G(v,x) = d_G(u,x)+1. \end{aligned}$$
  Now, by the triangle inequality
  $$\begin{aligned} d_G(u,x) \le d_G(u,v)+d_G(v,x)=d_G(u,v)+1. \end{aligned}$$
  Hence, overall, we get that $\ell _u(v) \le d_G(u,v) +2$. Note that we get the $d_{H_u}(u,v) \le d+2$, which implies that the distance is indeed maintained correctly in the monotone ES-tree.
2. *v* does not have a neighbor in $A_1$. In this case, all the edges adjacent to *v* are in $E_2 \subseteq H_u$. Let $\{w,v\}$ be the edge adjacent to *v* in the shortest path between *u* and *v* in *G*. Then, $d_G(u,w) = d_G(u,v)-1$. From the induction hypothesis, we have that $\ell _u(w) \le d_G(u,w) +2$. Since the edge $\{w,v\}$ exists in $H_u$, we get that
  $$\begin{aligned} \ell _u(v) = d_{H_u}(u,v) \le d_{H_u}(u,w) + d_{H_u}(w,v)= \ell _u(w)+1 \le d_G(u,w)+3 = d_G(u,v) + 2. \end{aligned}$$
  As $d_{H_u}(u,v) \le d+2$, we indeed maintain the value correctly in the monotone ES-tree.

**Induction Step.** Assume now that the claim holds after t-1 edge deletions, and we will prove it holds after *t* edge deletions. We prove it by induction on the length of the path between *u* and *v* in the current graph *G*. In the base case that u=v it trivially holds. First, if the value $\ell _u(v)$ did not change after the *t*-th edge deletion, then the claim follows from the induction hypothesis (as the distance $d_G(u,v)$ can never decrease). Hence, we focus on the case that $\ell _u(v)$ changed. By parts (3) and (4) of Lemma 2.4, when the level $\ell _u(v)$ is changed it is necessarily increased, and the new value is equal to $\min \limits _{x\in N(v)} \{\ell _u(x)+w_{H_u}(x,v) \}$.

1. When the value $\ell _u(v)$ increases, *v* has a neighbor in $A_1$. By Step 3 of the algorithm for maintaining the ES-tree, it is guaranteed that there is an edge $\{v,x\} \in E^* \subseteq H_u$. Also, $\ell _u(x)=d_G(u,x)$ by Claim 6.1 and since $d_G(u,x) \le d+1$, as *x* is a neighbor of *v*. Hence, when we update the value $\ell _u(v)$, the new value is at most $\ell _u(x)+w_{H_u}(x,v)=d_G(u,x)+1.$ The exact same proof that we used in the base case, shows that $d_G(u,x) \le d_G(u,v)+1$. Hence, $\ell _u(v) \le d_G(u,v) +2$, as needed. Again, as this value is bounded by d+2 it is maintained correctly.
2. When the value $\ell _u(v)$ increases, *v* does not have a neighbor in $A_1$. It follows from Step 3 of the algorithm for maintaining the ES-tree that all the edges adjacent to *v* are in $H_u$. Let *x* be the neighbor of *v* in the shortest u-v path in *G*. By the induction hypothesis, $\ell _u(x) \le d_G(u,x)+2$. Hence,
  $$\begin{aligned} \ell _u(v) \le \ell _u(x)+w_{H_u}(x,v) \le d_G(u,x)+3 = d_G(u,v)+2, \end{aligned}$$
  this value is again at most d+2, hence it is maintained correctly, completing the proof.

□

#### Time Complexity

To analyze the time complexity of the algorithm, we first bound the total number of edges added to $E_2$ and $E^*$ in the algorithm.

##### Claim 6.3

The total number of edges added to $E_2$ during the algorithm is bounded by $O(ns_1)$ with high probability.

##### Proof

By definition, the edges in $E_2$ are the edges adjacent to all nodes that do not have a neighbor in $A_1$. We show that as long as the degree of a node *v* is $s_1$ then with high probability it has a neighbor in $A_1$. This follows as $A_1$ is obtained by sampling each node with probability $\frac{c \ln {n}}{s_1}$. Hence, as long as the degree of *v* is at least $s_1$, the probability that none of its neighbors is in $A_1$ is at most $(1-\frac{c\ln {n}}{s_1})^{s_1} \le e^{-c\ln {n}}=\frac{1}{n^c}$. It follows that with high probability each node adds less than $s_1$ edges to $E_2$, proving the claim. □

##### Claim 6.4

The expected number of edges to ever be present in $E^*$ is $O( (mn)^{1/2}\log {n} )$.

##### Proof

Recall that each node is added to the set $A_1$ with probability $\frac{c \ln {n}}{s_1}$. Hence, in expectation each node *v* has $O(\deg (v) \frac{\log {n}}{s_1})$ neighbors in the set $A_1$, where deg(v) is the degree of *v* at the beginning of the algorithm. These edges are candidates to be added to the set $E^*$, and in the worst case all of them are added to $E^*$ during the algorithm. Summing over all nodes, the expected number of edges added to $E^*$ is bounded by

$$\begin{aligned} \sum _{v \in V} O \left( \frac{\deg (v)\log {n}}{s_1} \right) = O \left( \frac{m\log {n}}{(\frac{m}{n})^{1/2}} \right) = O( (mn)^{1/2}\log {n} ).{{\square }} \end{aligned}$$

##### Lemma 6.5

The total update time of the algorithm is $\tilde{O}(m^{1/2}n^{3/2}d)$ in expectation.

##### Proof

Computing the set $A_1$ takes *O*(*n*) time by sampling each node to the set independently with probability $\frac{c \ln {n}}{s_1}$.

Maintaining the sets $E^*$ and $E_2$ is done as follows. At the beginning of the algorithm, each node *v* scans its neighbors until it finds the first neighbor *u* in the set $A_1$ if exists, and it adds the edge $\{v,u\}$ to the set $E^*$. If there is no such *u*, then *v* adds all its adjacent edges to the set $E_2$. During the algorithm, when the edge $\{v,u\}$ is deleted, *v* continues scanning its neighbors until it finds the next neighbor *w* in the set $A_1$ if exists. If *w* exists it adds the edge $\{v,w\}$ to $E^*$, and otherwise it adds all its adjacent edges to $E_2$. Note that in the whole algorithm, *v* only needs to scan its neighbors once to find all the edges it adds to $E^*$. Hence, overall maintaining the sets $E^*$ and $E_2$ takes *O*(*m*) time. We can also keep a list of the new edges added to $E^*$ and $E_2$ after a deletion, to help maintaining the monotone ES-trees.

We maintain the ES-trees from nodes $u \in A_1$ as follows. Every time that an edge is deleted we delete it from all the trees. In addition, every time that a level $\ell _u(v)$ increases for $u \in A_1, v \in V {\setminus } A_1$, we update the weight of the edge $\{v,u\}$ in the graph $H_v$, and update it in the monotone ES-tree rooted at *v*. Maintaining the ES-trees from nodes in $A_1$ takes $O(|A_1|(md+n))$ time by Lemma 2.3. By the definition of $A_1$, this equals

$$\begin{aligned} O \left( \frac{n \log {n}}{s_1} \left( md \right) + n^2 \right) =O(m^{1/2}n^{3/2} (\log {n}) d) \end{aligned}$$

in expectation, when m=Ω(n).

We maintain the monotone ES-trees rooted at nodes $u \in V {\setminus } A_1$ as follows. After an edge *e* is deleted, we update the weights of the edges $\{u,v\} \in E_u^{A_1}$ when their weights are increased in the ES-trees rooted at nodes $v \in A_1$, as described above. In addition, we add to $H_u$ all the edges that were added to $E_2$ and $E^*$ after the deletion of the edge *e* (we kept them in a list as described above), and finally we delete the edge *e* from the monotone ES-tree. By Lemma 2.5, the total update time for maintaining the monotone ES-tree rooted at the node *u* is $O(\mathcal {E}(H_u)\cdot d+\mathcal {W}(H_u)+n)$, where $\mathcal {E}(H_u)$ is the total number of edges ever contained in $H_u$ and $\mathcal {W}(H_u)$ is the total number of updates to $H_u$. There are *O*(*n*) edges in $E_u^{A_1}$ and each of them is updated *O*(*d*) times, as the distance estimates only increase and can have *O*(*d*) values. This adds *O*(*nd*) term to the complexity of one tree, and $O(n^2 d)$ summing up over all trees. The edges in $E_2$ and $E^*$ are only added and removed once in the algorithm. The total number of edges in $E_2$ is $O(ns_1)$ with high probability from Claim 6.3. Summing up over all trees these edges add $O(n^2 s_1 d)= O(n^{3/2} m^{1/2} d)$ term to the complexity. The total number of edges in $E^*$ is bounded by $O( (mn)^{1/2}\log {n} )$ in expectation from Claim 6.4. Summing up over all trees they add $O(n^{3/2} (\log {n}) m^{1/2} d)$ term to the complexity, completing the proof. □

##### Claim 6.6

The query time of the algorithm is *O*(1).

##### Proof

To answer a distance query for a pair of nodes *u*, *v*, we just return the value $\ell _u(v)$. This can be done in *O*(1) time if we keep for each node *u* an array of the values $\ell _u(v)$ which is updated each time a distance estimate change. □

In conclusion, we get the following.

##### Theorem 6.7

Given an undirected unweighted graph *G*, there is a decremental data structure that maintains +2-additive approximation for APSP for pairs of nodes at distance at most *d* from each other. The expected total update time is bounded by $\tilde{O}(m^{1/2}n^{3/2}d)$, and the query time is constant.

To get rid of the *d* term in the complexity, we can combine our approach with Theorem 2.6. This would lead to a (1+ϵ,2)-approximation and replace the *d* term in the complexity by an $O(1/\epsilon )^{O(\sqrt{\log {n}})}$ term (which simplifies to $n^{o(1)}$ for constant ϵ. We will provide full details of the approach when we describe our general algorithm in the next section (see in particular Sect. 6.2.4). The high-level idea is to use our algorithm with a parameter $d=O(1/\epsilon )^{O(\sqrt{\log {n}})}$, and combine it with Theorem 2.6 for computing $(1+\epsilon ,\beta )$-approximation for large distances. For an appropriate choice of the parameters, we will get a (1+ϵ)-approximation for the distances between pairs of nodes at distance greater than *d* from each other, and a +2 approximation for pairs of nodes at distance at most *d* from each other.

### (1+ϵ,2k-2)-APSP

With this warm-up we can move on to the more general algorithm based on [24] that leads to a larger stretch but a smaller update time. Our goal is to obtain a (1+ϵ,2k-2)-approximation in $\tilde{O}(n^{2-1/k}m^{1/k}(1/\varepsilon )^{O(\sqrt{\log n})})$ time. The algorithm works in unweighted undirected graphs. As before, we start by describing a data structure that maintains the approximate distances for pairs of nodes at distance at most *d* from each other, and then in Sect. 6.2.4 we combine it with Theorem 2.6 to handle the general case.

#### Dynamic Data Structure

The algorithm is summarized as follows.

1. **Node sets** $A_i$. We define a series of sets $A_1,A_2,...,A_k$ such that for i=1,...,k-1, the set $A_i$ is a random set obtained by sampling each node with probability $p_i=\frac{c\ln {n}}{s_i}$, for $s_i=(\frac{m}{n})^{1-i/k} (\ln {n})^{i/k}$, and a constant c≥2 for our choice. If a node is sampled to several different sets we keep it only in the set with minimum index *i* among them. Finally, we define $A_k = V \setminus \bigcup _{i=1}^{k-1} A_i.$ Note that by definition, each node is in exactly one set $A_i$.
2. **Indices** $i_v$ **for all** v∈V. For a node v∈V, we denote by $i_v$ the minimum index *i* such that *v* has a neighbor in $A_i$.
3. **Edge set** $E^*$. For each node *v*, we add one edge $\{v,x\}$ to $E^*$, where *x* is an arbitrary neighbor of *v* in $A_{i_v}$.
4. **Edge sets** $$E_i$$. The edges $$E_i$$ are all the edges $$\{u,v\}$$ where $$\max \{i_u,i_v\} \ge i$$. In other words, it is all the edges adjacent to nodes that do not have a neighbor in $$A_{i'}$$ for $i^{\prime }<i$.
5. **Maintaining monotone ES-trees.** The algorithm maintains monotone ES-trees as follows.
  For every $u \in A_1$, we maintain an ES-tree rooted at *u* up to distance d+3k in the graph *G*.
  For i=2 to *k*:
  For every $$u \in A_i$$, we maintain a monotone ES-tree rooted at *u* up to distance d+3k in the graph $$H_u^i=(V,E_i \cup E^* \cup E_u^i)$$, where $$E_u^i$$ are all the edges $$\{u,v\}$$, where $$v \in A_{i'}$$ for $i^{\prime }<i$. The weight of the edge $$\{u,v\}$$ is equal to the value $$\ell _v(u)$$ in the monotone ES-tree rooted at *v*.

Note that we maintain the monotone ES-trees up to distance d+3k. As we show in the stretch analysis, this is needed in order to guarantee that we maintain correct estimates for any pair of nodes at distance at most *d* from each other. Intuitively, as the distance estimate we maintain is at most d(u,v)+2k, we would like to maintain correct estimates for pairs of nodes at distance d+2k from each other. Another *k* term comes from the inductive argument of the stretch analysis. For more details, see Sect. 6.2.2.

In Sect. 6.2.3, we explain in detail how to maintain the data structures efficiently. When we maintain the graphs $H_u^i$ we make sure that the following holds (for a proof, see Sect. 6.2.3).

##### Claim 6.8

Let $$u \in A_i, v \in V$$. Throughout the updates, it always holds that *v* either has an edge $$\{v,w\} \in H_u^i$$, where $$w \in A_{i'}$$ for $i^{\prime }<i$, or all the edges adjacent to *v* are in $$H_u^i$$.

We are in the first case of the claim when $i_v <i$, and we are in the second case when $i_v \ge i$.

#### Stretch Analysis

We need the following claims. First, from part (1) of Lemma 2.4 we have the following.

##### Claim 6.9

At the beginning of the algorithm, for any $u \in A_i$, and any *v* at distance at most d+3k from *u* in $H_u^i$, we have $\ell _u(v)=d_{H_u^i}(u,v).$

We next show the following.

##### Claim 6.10

Let $u \in A_i, w \in A_j$ such that j<i, then $\ell _u(w) \le \ell _w(u)$.

##### Proof

The claim follows as we always have an edge $\{u,w\}$ with weight $\ell _w(u)$ in the graph $H_u^i$, and the value $\ell _w(u)$ is monotonically increasing during the algorithm. Hence, and from part (4) of Lemma 2.4, every time that the estimate $\ell _u(w)$ changes it always changes to a value that is at most $\ell _w(u)$. □

We next prove the stretch guarantee of the algorithm.

##### Lemma 6.11

For any node $u \in A_i$, and any node v∈V where $d_G(u,v) \le d+(k-i)$, throughout the updates we have $d_G(u,v) \le \ell _u(v) \le d_G(u,v)+ 2(i-1)$.

##### Proof

First, the left inequality holds since $$d_G(u,v) \le d_{H_u^i}(u,v) \le \ell _u(v)$$, where the first inequality follows from the definition of $$H_u^i$$, and the second follows from part (2) of Lemma 2.4. We prove the right inequality by triple-induction, on the value *i*, on the time in the algorithm, and on the distance $$d_G(u,v)$$. For i=1, the claim follows as we maintain ES-trees up to distance d+3k from the nodes of $$A_1$$ in the graph *G*. We next assume that it holds for any $i^{\prime }<i$, and prove that it holds for *i*. The proof is by induction on the time in the algorithm and on the distance $$d_G(u,v)$$.

**Base Case.** We first show that the claim holds at the beginning of the algorithm. From Claim 6.9, at the beginning of the algorithm, for any *u*, *v* where $d_{H_u^i}(u,v) \le d+3k$, we have that $\ell _u(v) = d_{H_u^i}(u,v).$ Hence, our goal is to show that $d_{H_u^i}(u,v) \le d_G(u,v) + 2(i-1)$. Note that if this holds, then since $d_G(u,v) \le d+(k-i)$, we get that $d_{H_u^i}(u,v) \le d+3k$, hence the distance is maintained in the monotone ES-tree as needed. The proof is by induction on $d_G(u,v)$.

1. $$i_v < i$$. In this case, there is an edge $$\{v,w\} \in E^* \subseteq H_u^i$$ such that $$w \in A_{i'}$$ for $i^{\prime }<i$. Note that since $$d_G(u,v) \le d+(k-i)$$, and *w* is a neighbor of *v*, then we have $$d_G(u,w) \le d +(k-i)+1 = d+(k-(i-1)) \le d+(k-i')$$. Hence, we can use the induction hypothesis on *w* and *u*. From the induction hypothesis and Claim 6.9, we have $$\ell _w(u) = d_{H_u^i}(w,u) \le d_G(w,u)+2(i-2)$$ (note that this value is smaller than d+3k, so we can indeed use Claim 6.9). Hence, we get that
  $$\begin{aligned} d_{H_u^i}(u,v) \le d_{H_u^i}(u,w) + d_{H_u^i}(w,v) \le d_G(w,u) + 2(i-2)+1. \end{aligned}$$
  We have, $d_G(w,u) \le d_G(w,v)+d_G(v,u)=d_G(u,v)+1$. Hence, we get, $d_{H_u^i}(u,v) \le d_G(u,v)+2(i-1)$, as needed.
2. $i_v \ge i$. In this case, by definition, all the edges adjacent to *v* are in $E_i \subseteq H_u^i$. Let *w* be the neighbor of *v* on the shortest u-v path. From the induction hypothesis, $d_{H_u^i}(u,w) \le d_G(u,w)+2(i-1)$. So we have
  $$\begin{aligned} d_{H_u^i}(u,v)\le &   d_{H_u^i}(u,w)+d_{H_u^i}(w,v)\\  \le &   d_G(u,w)+1+2(i-1)=d_G(u,v)+2(i-1). \end{aligned}$$

**Induction Step.** We assume that the claim holds after t-1 edge deletions, and show that it holds after *t* edge deletions. The proof is by induction on the distance $d_G(u,v)$. If u=v, the claim trivially holds. If the estimate $\ell _u(v)$ did not change after the *t*’th edge deletion, then the claim holds from the induction hypothesis. Note that an edge deletion can potentially lead to several changes of the value $\ell _u(v)$. It is enough to prove that the stretch guarantee holds in the last time the value $\ell _u(v)$ increases, as the value stays the same afterwards. We divide into cases based on Claim 6.8.

1. When the value $$\ell _u(v)$$ increases in the last time, there is an edge $$\{v,w\} \in H_u^i$$ such that $$w \in A_{i'}$$ for $i^{\prime }<i$. From part (4) of Lemma 2.4, when $$\ell _u(v)$$ changes, it gets the value $$\min \limits _{x\in N(v)} \{\ell _u(x)+w_{H_u^i}(x,v) \}$$, assuming this value is at most d+3k, and we will show that this is indeed the case. Hence,
  $$\begin{aligned} \ell _u(v) \le \ell _u(w)+w_{H^i_u}(w,v) \le \ell _w(u) + 1 \le d_G(u,w)+2(i-2)+1. \end{aligned}$$
  Here we use Claim 6.10 and the fact that $$w \in A_{i'}$$ for $i^{\prime }<i$ (note that since $$d_G(u,v) \le d+(k-i)$$, and *w* is a neighbor of *v*, it follows that $$d_G(u,w) \le d+(k-i')$$, so we can indeed use the induction hypothesis). It also holds that $$d_G(u,w) \le d_G(u,v)+d_G(v,w)=d_G(u,v)+1$$. Hence, overall, we have $$\ell _u(v) \le d_G(u,v) + 2(i-2)+2=d_G(u,v)+2(i-1)$$, as needed. Note that this value is indeed smaller than d+3k, hence we maintain the correct estimate in the monotone ES-tree.
2. When the value $\ell _u(v)$ increases in the last time, all the adjacent edges to *v* are in the graph $H_u^i$. Let *w* be the neighbor of *v* on the shortest u-v path. When the distance estimate of *v* changes we have
  $$\begin{aligned} \ell _u(v) \le \ell _u(w)+d_{H_u^i}(w,v) \le d_G(u,w) + 2(i-1) + 1 = d_G(u,v)+2(i-1), \end{aligned}$$
  where we use the induction hypothesis on *w* and part (4) of Lemma 2.4. Note that it follows that the values $\ell _u(v),\ell _u(w)$ are smaller than d+3k, hence they are maintained correctly in the monotone ES-tree.

□

#### Time Complexity

We next analyze the time complexity of the algorithm. We start by showing that the first steps of the algorithm can be maintained efficiently.

##### Claim 6.12

The first 4 steps of the algorithms can be maintained in O((m+n)k) time.

##### Proof

**Step **1. Computing the sets $A_i$ takes *O*(*kn*) time, by just sampling each node to the set $A_i$ with probability $p_i$. For each node *v*, we can stop the sampling after the first time *v* is sampled to one of the sets. This is only done once at the beginning of the algorithm. For each node *v*, we keep the index *i* such that $v \in A_i$. For each *i*, we create a list of all the nodes in the set $A_i$. Creating all these lists takes *O*(*n*) time by scanning the nodes.

**Steps **2 **and** 3. The indices $i_v$ and the set $E^*$ are maintained dynamically in the algorithm, as follows. At the beginning, each node *v* scans its neighbors to find the minimum index *i* such that *v* has a neighbor in $A_i$, it also saves the name of an edge $\{v,u\}$ to the first neighbor of *v* in $A_{i_v}$, this edge is added to set $E^*$. In the algorithm, if the edge $\{v,u\}$ is deleted, then *v* goes over its next neighbors, and finds the next neighbor $w \in A_{i_v}$ if exists. If such a neighbor exists, then $\{v,w\}$ is added to $E^*$. Otherwise, the value $i_v$ increases, and *v* scans its neighbors again to determine the new index $i_v$ and adds the first edge $\{v,w\}$ from *v* to a neighbor in $A_{i_v}$ to $E^*$. Note that overall in the algorithm each node needs to scan its neighbors *O*(*k*) times, because as long as the value $i_v$ stays the same, we just need to go over the neighbors once during the whole algorithm to determine all the edges added to $E^*$. Overall these steps take *O*(*mk*) time.

**Step **4. Maintaining the sets $$E_i$$ is done as follows. Initially, we add to $$E_i$$ all the edges adjacent to nodes *v* where $$i_v \ge i$$. During the algorithm, every time that the value $$i_v$$ of a node *v* changes, say from *j* to $j^{\prime }$, then for all $j<i\leq j^{\prime }$, we add the edges adjacent to *v* to the set $$E_i$$. We also keep a list of all the newly added edges in $$E_i$$ after a deletion of an edge, so that we can update them in all the relevant monotone ES-trees. The time complexity for maintaining the set $$E_i$$ is proportional to the number of edges added to $$E_i$$ during the algorithm, which can trivially be bounded by *O*(*m*), hence this step takes at most *O*(*mk*) time. □

**Maintaining the monotone ES-trees.** The main challenge in the algorithm is maintaining the monotone ES-trees in Step 5 of the algorithm. Before analyzing the time complexity of the algorithm, we start by describing the algorithm in more detail. Note that each time an edge is deleted it can lead to many changes, including edge insertions and weight changes in all the trees we maintain. We next explain in detail in which order we make these changes in order to maintain the correctness of the algorithm. We use the algorithms for ES trees and monotone ES trees described in Sect. 2.4.

**ES-trees rooted at nodes of** $A_1$. First, for any node $u \in A_1$, we start by creating an ES-tree rooted at *u* in the graph *G*, maintained up to distance d+3k. After creating this tree, for any $v \in A_i$ for i>1, we add an edge $\{u,v\}$ with weight $\ell _u(v)$ to the set of edges $E_v^i$. During the algorithm, every time an edge is deleted, we delete it from the ES-tree and update it accordingly. For any *v* where $\ell _u(v)$ has increased after the deletion and where $v \in A_i$ for i>1, we update the corresponding edge in $E_v^i$, and save it in a list of edges in $E_v^i$ that were updated after the deletion. This completes the description for $u \in A_1$.

**Monotone ES-trees rooted at nodes of** $$A_i$$. For $$u \in A_i$$ for larger values of *i*, we maintain the monotone ES-trees according to the order of the index *i*, from 2 to *k*. We work as follows. Initially, we create a monotone ES-tree rooted at *u* up to distance d+3k in the graph $$H_u^i=(V,E_i \cup E^* \cup E_u^i)$$, with the edges $$E_i,E^*$$ at the beginning of the algorithm, and with the edges $$E_u^i$$ that were obtained when creating the monotone ES-trees in previous levels. Note that all these edges of $$E_u^i$$ are of the form $$\{u,v\}$$ where $$v \in A_{i'}$$ for $i^{\prime }<i$, so we indeed already created the monotone ES-tree rooted at the relevant nodes *v*. After creating the tree, for any node $$v \in A_{i'}$$ for $i^{\prime }>i$, we add a new edge to the set $$E_v^{i'}$$ with weight $$\ell _u(v)$$. During the algorithm, when an edge *e* is deleted, we update the monotone ES-trees in several steps as follows, again we work according to the order of the index *i*.

- *First step: adding the new edges of *$E^*$ *and* $E_i$ *to* $H_u^i$. The deletion of the edge *e* can lead to adding new edges to $E^*$ or $E_i$ in steps 3 and 4 of the algorithm. As explained in these steps, we maintain the newly added edges, and can add them to a list of newly added edges (to $E^*$ and to $E_i$ separately). When we update the monotone ES-tree rooted at $u \in A_i$, we start by adding all these newly added edges that were added to $E^*$ and $E_i$ to $H_u^i$.
- *Second step: updating the edges in* $E_u^i$. Next, there are possibly edges in $\{u,v\} \in E_u^i$ that their weight is increased after the deletion. This is already observed when we update the monotone ES-tree rooted at *v*, and if it happens, we add the edge $\{u,v\}$ with new weight $\ell _v(u)$ to a list of edges that were updated in $E_u^i$. When we update the monotone ES-tree rooted at *u*, we update the weights of all these edges (we already have their list after we finish maintaining the ES-trees in previous levels).
- *Third step: deleting the edge* *e*. Lastly, we delete the edge *e* that was deleted in this step from the monotone ES-tree.

Note that the weight increases and deletion can increase the levels $\ell _u(v)$ of some nodes in the tree. For any *v* in $A_j$ for j>i such that $\ell _u(v)$ increases, when the value increases, we add an edge $\{v,u\}$ with the new weight $\ell _u(v)$ to the list of newly updated edges in $E_v^j$. This completes the description of the algorithm. From the above description, we can now prove Claim 6.8.

##### Proof of Claim 6.8

Let $$u \in A_i, v \in V$$. At the beginning of the algorithm, if $$i_v < i$$, then there exists an edge $$\{v,w\} \in E^* \subseteq H_u^i$$ where $$w \in A_{i'}$$ for $i^{\prime }<i$. Otherwise, $$i_v \ge i$$, and all the edges adjacent to *v* are in $$E_i \subseteq H_u^i$$. During the algorithm, every time an edge *e* of the form $$\{v,w\} \in E^*$$ is deleted, then if $$i_v < i$$ after the deletion, we add a new edge $$\{v,w'\}$$ to $$E^*$$ for $$w' \in A_{i'}$$ for $i^{\prime }<i$, and if $$i_v \ge i$$, we add all the edges adjacent to *v* to $$E_i \subseteq H_u^i$$. In the algorithm, we first add these new edges to $$H_u^i$$, and only then delete the edge *e*, which proves the claim. □

To bound the time complexity, we need the following useful claims that bound the total number of edges added to the sets $E_i$ and $E^*$ during the algorithm.

##### Claim 6.13

For i≥2, the total number of edges added to $E_i$ during the algorithm is bounded by $O(ns_{i-1})$ with high probability.

##### Proof

The edges $$E_i$$ are all the edges adjacent to nodes *v* where $$i_v \ge i$$. We show that at the first time in the algorithm that $$i_v \ge i$$, the degree of *v* is at most $$s_{i-1}$$ with high probability. This implies that each node *v* adds at most $$s_{i-1}$$ edges to the set $$E_i$$, proving the claim. To bound the degree of *v*, note that by definition the set $$A_{i-1}$$ is obtained by sampling each node with probability $$\frac{c \ln {n}}{s_{i-1}}$$, all these nodes are added to the set $$A_{i-1}$$, unless they are already part of a set $$A_{i'}$$ for $i^{\prime }<i-1$. As long as the degree of *v* is at least $$s_{i-1}$$, the probability that none of the neighbors of *v* is sampled to $$A_{i-1}$$ is at most $$(1-\frac{c\ln {n}}{s_{i-1}})^{s_{i-1}} \le e^{-c\ln {n}}=\frac{1}{n^c}$$. Hence, with high probability *v* has a neighbor sampled to $$A_{i-1}$$, implying that $$i_v \le i-1$$ as long as $$\deg (v) \ge s_{i-1}$$. From union bound, this holds for all nodes with high probability. This proves that the total number of edges added to $$E_i$$ in the algorithm is bounded by $$O(ns_{i-1})$$. Note that the proof assumes that we have an oblivious adversary and that the order in which edges are deleted does not depend on the random choices of the algorithm. □

##### Claim 6.14

In expectation, the total number of edges added to the set $E^*$ in the algorithm is bounded by

$$\begin{aligned} O(kn^{1-1/k}m^{1/k}(\log {n})^{1-1/k}). \end{aligned}$$

##### Proof

We analyze the number of edges *v* adds to the set $E^*$ when $i_v=i$ for all *i*. First, when $i_v=1$, then in the worst case *v* adds to $E^*$ edges to all its neighbors in $A_1$. By the definition of $A_1$, each node is added to $A_1$ with probability $\frac{c\ln {n}}{s_1}$. Hence, in expectation *v* has $O(\frac{\deg (v) \log {n}}{s_1})$ neighbors in $A_1$, where deg(v) is the degree of *v* at the beginning of the algorithm. When we sum up over all *v*, this adds at most $O(\frac{m \log {n}}{s_1})=O(n^{1-1/k}m^{1/k}(\log {n})^{1-1/k})$ edges in expectation, where the last equality follows from the definition of $s_1$.

When $i_v=i$ for i>1, as we proved in Claim 6.13, with high probability the degree of *v* is at most $s_{i-1}$. Note that each one of the neighbors of *v* is added to $A_i$ with probability $\frac{c\ln {n}}{s_i}$. Hence, in expectation, *v* has $O(s_{i-1} \cdot \frac{\ln {n}}{s_i})$ neighbors in $A_i$. In the worst case, it adds edges to all of them during the algorithm. In total, the expected number of edges added by all nodes to $E^*$ in the case that $i_v \ge 2$ is bounded by $\sum _{i=2}^{k} O(\frac{\ n \log {n} s_{i-1}}{s_i})$. By the definition of $s_i$, we have that $\frac{s_{i-1}}{s_i}=(\frac{m}{n})^{1/k}(\ln {n})^{-1/k}$. Hence, in total the expected number of edges added is bounded by $O(k n^{1-1/k}m^{1/k}(\log {n})^{1-1/k}).$ □

Next, we prove the complexity of the algorithm.

##### Lemma 6.15

Maintaining the ES-trees takes $O(kn^{2-1/k}m^{1/k}(\log {n})^{1-1/k} d)$ time in expectation.

##### Proof

Maintaining the ES-trees rooted at nodes in $A_1$ takes $O(|A_1|(md+n))$ time by Lemma 2.3. Since $|A_1| = O(\frac{n\log {n}}{s_1})$ in expectation, and by the choice of $s_1$, this adds $O(n^{2-1/k}m^{1/k}(\log {n})^{1-1/k} d)$ term to the complexity, when m=Ω(n).

From Lemma 2.5, maintaining a monotone ES-tree rooted at $u \in A_i$ takes $O(\mathcal {E}(H_u^i)\cdot d+\mathcal {W}(H_u^i)+n)$ time, where $\mathcal {E}(H_u^i)$ is the total number of edges ever contained in $H_u^i$ and $\mathcal {W}(H_u^i)$ is the total number of updates to $H_u^i$. To complete the proof, we should analyze these values. Recall that $H_u^i=(V, E_i \cup E^* \cup E_u^i)$. Note that $E_u^i$ has at most n-1 edges, that are updated *O*(*d*) times during the algorithm, as we maintain distances up to distance d+3k, hence these edges add *O*(*nd*) term to the complexity of maintaining one tree, and $O(n^2 d)$ for all *n* trees. The edges in $E^*$ and $E_i$ are only added and removed once from a tree during the algorithm. From Claim 6.13, the total number of edges added to $E_i$ during the algorithm is bounded by $O(ns_{i-1})$ with high probability. Since these edges are part of all the ES-trees rooted at nodes in $A_i$, maintaining these edges adds $O(|A_i| n s_{i-1} d)$ term to the complexity. This equals

$$\begin{aligned} O \left( \frac{n \log {n}}{s_i} \cdot n s_{i-1} \cdot d \right) = O( n^{2-1/k}m^{1/k}(\log {n})^{1-1/k} d), \end{aligned}$$

following the same calculations done in Claim 6.14 (but with an extra *nd* term). Summing over all *k* adds another multiplicative *k* term to the complexity. From Claim 6.14, the total number of edges added to $E^*$ is bounded by $O(kn^{1-1/k}m^{1/k}(\log {n})^{1-1/k})$ in expectation. Summing over all *n* trees, it adds another $O( kn^{2-1/k}m^{1/k}(\log {n})^{1-1/k} d)$ term to the complexity, completing the proof. □

##### Claim 6.16

The query complexity of the algorithm is constant.

##### Proof

To answer a distance query for a pair of nodes *u*, *v*, we work as follows. If $u \in A_i, v \in A_j$, and i<j, our distance estimate is $\ell _u(v)$, otherwise our distance estimate is $\ell _v(u)$. Note that it is easy to maintain the values $\ell _u(v)$ in a way that the query time is constant. For example, we can maintain for each node *u* an array with the values $\ell _u(v)$, and update the value in constant time each time it was changed in the algorithm. □

#### Putting Everything Together

In the last sections we showed an algorithm that for any pair of nodes at distance at most *d* maintains a +2(k-1)-additive approximation in $\tilde{O}(n^{2-1/k}m^{1/k}d)$ time. To get our final result, we combine it with Theorem 2.6 to handle large distances, proving the following.

**Theorem** 1.4*Given an undirected unweighted graph* *G*, *a constant* 0<ϵ<1 *and an integer* $2 \le k \le \log {n}$, *there is a decremental data structure that maintains* (1+ϵ,2(k-1))-*approximation for APSP with constant query time. The algorithm works against an oblivious adversary. The expected total update time is bounded by* $\min \{O(n^{2-1/k+o(1)}m^{1/k}), \tilde{O}((n^{2-1/k}m^{1/k}) O(1/\epsilon )^{k/\rho })\}$ *where* $m=n^{1+\rho }$.

Note that for small values of *k* and if $m=n^{1+\rho }$ for a constant ρ>0, we get a complexity of $\tilde{O}(n^{2-1/k}m^{1/k})$, where in the general case we have an extra $n^{o(1)}$ term.

##### Proof

We maintain simultaneously our dynamic data structure to handle short distances of at most *d*, and the data structure from Theorem 2.6 to handle large distances of more than *d*, for a parameter *d* which we specify next. We run the algorithm from Theorem 2.6 with parameters $$\epsilon ' = \epsilon /2$$, and $k^{\prime }$ to be specified later. Recall that this algorithm gives a $$(1+\epsilon ', \beta )$$-approximation for $$\beta = 2(1+2/\epsilon ')^{k'-2}$$. We define $$d=\beta /\epsilon '$$.

To answer a distance query for a pair of nodes *u*, *v*, we return the minimum between the values obtained in both algorithms. As in both algorithms the query time is constant, we get a constant query time.

For the stretch analysis, if d(u,v)≤d, then by Lemma 6.11, we get an additive stretch of at most +2(k-1). If $d(u,v) \ge d = \beta /\epsilon '$, then the distance estimate we get from the $(1+\epsilon ',\beta )$-approximation algorithm is at most

$$\begin{aligned} (1+\epsilon ')d(u,v) + \beta \le (1+\epsilon ')d(u,v) + \epsilon ' d(u,v) = (1+2\epsilon ')d(u,v)=(1+\epsilon )d(u,v). \end{aligned}$$

Hence, overall we get a (1+ϵ,2(k-1))-approximation, as needed.

We now analyze the time complexity. From Lemma 6.15, the time complexity of our algorithm (for small distances) is $$O(kn^{2-1/k}m^{1/k}(\log {n})^{1-1/k} d)$$, and from Theorem 2.6 the time complexity of the near-additive approximation algorithm is $$\tilde{O}(n^{2+1/k'} (37/\epsilon )^{k'-1})$$. We distinguish between two choices for the value $k^{\prime }$. First we choose $k^{\prime }=k/\rho$, where $$m=n^{1+\rho }$$. In this case, the complexity of both expressions is bounded by $$\tilde{O}((n^{2-1/k}m^{1/k}) O(1/\epsilon )^{k/\rho })$$, as $$d=\beta /\epsilon ', \beta = 2(1+2/\epsilon ')^{k'-2}$$, and $$O(n^{2-1/k}m^{1/k})=O(n^{2-1/k}n^{(1+\rho )/k})=O(n^{2+\rho /k})=O(n^{2+1/k'})$$.

Second, we choose $k' = \sqrt{\log {n}}$. In this case, $\tilde{O}(n^{2+1/k'} (37/\epsilon )^{k'-1})= O(n^{2+o(1)})$. Similarly, $d= \beta /\epsilon ' = O(1/\epsilon ')^{k-1}= O(1/\epsilon )^{O(\sqrt{\log {n}})}=n^{o(1)}$, where the last equality follows since we assume ϵ to be a constant. Hence, in total we get a complexity of $O(n^{2-1/k+o(1)}m^{1/k})$, when m=Ω(n). □

## Conclusion

We presented new tradeoffs for decremental approximate APSP in undirected graphs. For weighted graphs, we obtained a (2+ε)-approximation with total update time $\widetilde{O}(m^{1/2}n^{3/2})$ and a faster $(2+\varepsilon ,W_{u,v})$-approximation with total update time $\widetilde{O}(nm^{3/4})$. For unweighted graphs, we obtained a (2+ε)-approximation with total update time $\widetilde{O}(m^{7/4})$, yielding the first subquadratic algorithm for sufficiently sparse graphs, as well as a family of (1+ε,2(k-1))-approximation algorithms with total update time $\widetilde{O}(n^{2-1/k}m^{1/k})$. These results demonstrate that approximation guarantees between 1+ε and 3, which have been extensively studied in the static setting [26–28, 35, 37, 45, 46], can also be exploited to obtain substantially faster decremental APSP algorithms.

Despite this progress, the landscape of decremental APSP remains far from fully understood. One of the central open questions is to characterize for which approximation guarantees the total update time of a decremental APSP algorithm can match, up to subpolynomial factors, the running time of the best corresponding static algorithm. For single-source shortest paths, a remarkably complete picture has emerged through a long line of work culminating in near-linear update times for a wide range of approximation guarantees [1, 7, 8, 14, 18, 20, 74]. In contrast, for APSP there remain substantial gaps. For example, our (2+ε)-approximate weighted algorithm achieves total update time $\widetilde{O}(m^{1/2}n^{3/2})$, matching the classical static construction of Cohen and Zwick [27] up to polylogarithmic factors. However, since then faster static 2-approximations have been developed: Baswana and Kavitha [28] give an algorithm for 2-APSP taking $\tilde{O}(m\sqrt{n}+n^2)$ time. For dense graphs, Dory et al. [37] give (2+ϵ)-APSP in $O(n^{2.214})$ time and a distance oracle for 2-APSP taking $\tilde{O}(mn^{2/3})$ time. Determining whether these gaps between decremental and static can be closed, or whether they reflect inherent barriers, remains an open question.

A second major direction concerns robustness against stronger adversaries and deterministic computation. Most of the fastest decremental APSP algorithms, including ours, rely on randomization and are analyzed against an oblivious adversary [2, 4, 13]. On the other hand, substantial progress has been made in the deterministic setting for large-stretch approximations and related shortest-path problems [16–18, 21, 50, 73, 77, 89].

Nevertheless, no deterministic or adaptive-adversary decremental APSP algorithms are known with update times comparable to those achieved in the oblivious-adversary setting for approximation guarantees around 2+ε. Closing these gaps would represent a major advance in dynamic graph algorithms.

Finally, the fully dynamic setting remains largely unexplored in the approximation regime considered in this paper. Fully dynamic APSP has a long history, beginning with exact algorithms [48, 57, 58, 75, 76] and continuing with approximate algorithms [5, 12, 22]. However, the best known fully dynamic (2+ε)-approximate APSP algorithms still require roughly $m^{1+o(1)}$ amortized update time per operation [5], and there has been comparatively little progress toward achieving the kinds of improvements that are now known in the decremental setting. It remains open whether approximation guarantees between 2 and 3 admit substantially faster fully dynamic algorithms and whether techniques such as approximate bunches or hopsets can be extended to support insertions efficiently.

We hope that the techniques introduced in this work—particularly the dynamic maintenance of approximate bunches and the use of bunch-overlap thresholds—provide useful tools for addressing these questions and for further narrowing the gap between static, decremental, and fully dynamic shortest-path algorithms.

## Figures and Tables

**Algorithm 3:**
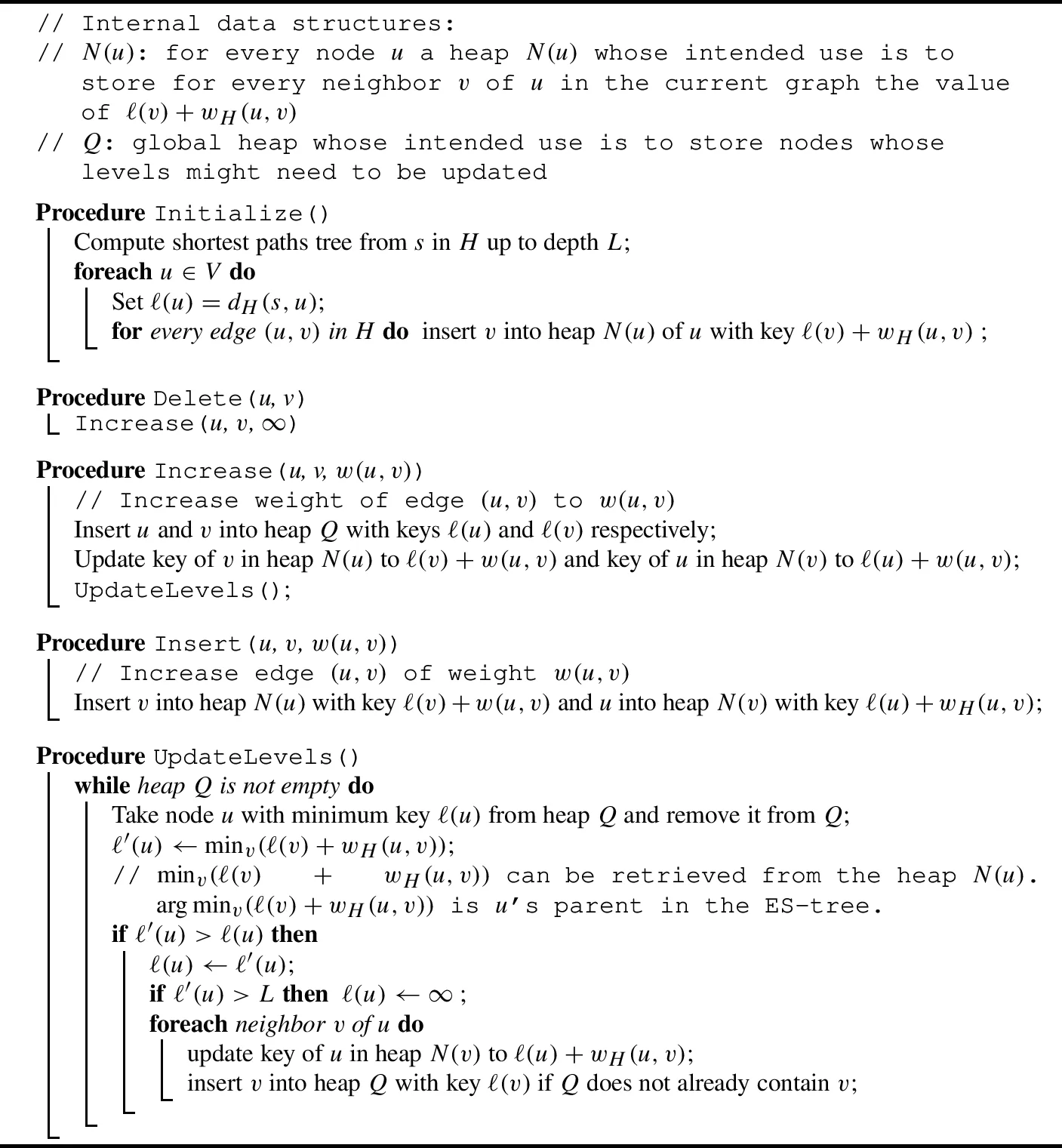
Monotone ES-tree

## Data Availability

No datasets were generated or analysed during the current study.

## Monotone ES-tree

Algorithm 4 provides pseudocode for the monotone ES-tree algorithm, this is taken from [8].

## Maintaining Bunches and Clusters

In this section we give a sketch of the algorithm of Łącki and Nazari [20] that allows us to maintain bunches and clusters needed for the decremental algorithm in Sect. 4. One tool used is a decremental algorithm by Roditty and Zwick [4] that maintains Thorup-Zwick clusters up to a bounded depth. This result can be summarized as follows:

### Lemma B.1

([4]). Let 0<p≤1 and d≥1 be parameters. Given a dynamic graph G=(V,E,w), that has edge deletions, weight increases, and edge insertions that do not decrease distances, we can maintain clusters (with sampling probability *p*) and bunches up to depth *d* in total update time $\tilde{O}(|E(G)|d/p)$. Moreover, we maintain distances from each node to its cluster and its bunch, and these distances are monotone (non-decreasing).

This time dependence on *d* is too expensive for many applications, and thus [20] improve the time for maintaining *approximate* bunches and clusters by maintaining a hopset, combined with a well-known scaling tool.

**Hopsets.** Hopsets, originally introduced by Cohen and Zwick [27] in the context of parallel algorithms, are widely used in faster algorithms in decremental settings. We briefly define this concept and sketch its application.

### Definition 2

Let G=(V,E,w) be a weighted undirected graph, and let 0<ϵ<1 be a parameter. A $(\beta ,1+\epsilon )$-hopset is a graph $H=(V,E(H),w_H)$ such that for each u,v∈V we have $d_G(u,v)\le d^{(\beta )}_{G\cup H}(u,v) \le (1+\epsilon ) d_G(u,v)$, where $d^{(\beta )}_{G\cup H}(u,v)$ denotes the length of the shortest path between *u* and *v* in G∪H that uses at most β hops. We say β is the *hopbound* of the hopset and 1+ϵ is the *stretch* of the hopset.

### Theorem B.2

([20]) Let $0< \rho< 1/2, 0< \epsilon <1$ be parameters. We can maintain a $(\beta ,1+\epsilon )$-hopset *H* for G=(V,E,w) with hopbound $\beta = O(\tfrac{\log (nW)}{\epsilon })^{2/\rho +1}$ in total update time $\tilde{O} (\tfrac{\beta \log ^2(nW)}{\epsilon }mn^{\rho })$. Moreover, we can assume that the distances incurred by the additional edges of the hopset are monotone. Further we have that *H* is of size $\tilde{O}(n^{1+\rho })$.

By setting $\rho =\frac{\log \log n}{\sqrt{\log n}}$, we can maintain a hopset with hopbound $2^{\tilde{O}(\sqrt{\log n})}$ in $2^{\tilde{O}(\sqrt{\log n})}$ amortized time. We often choose this parameter setting when the graph is sparse for balancing the hopset maintenance time with the shortest path computation time. By setting ρ to a constant, we can maintain a hopset of polylogarithmic hopbound in time $O(mn^{\rho })$. We often use this parameter setting when we are maintaining distances from many sources and the graph is slightly denser (e.g. $m=n^{1+\Omega (1)}$).

**Scaling.** The following scaling technique of Klein and Subramanian [90] changes a bounded hop distance problem into a bounded depth distance problem on a *scaled graph*. Similar ideas have been widely used in dynamic algorithms, see e.g., [5, 6, 8, 20]. As we will see, the bounded depth works well together with the cluster and bunch maintaining algorithm of Roditty and Zwick [4]

### Lemma B.3

([90]) Let G=(V,E,w) be a weighted undirected graph. Let R≥0, d≥1, ϵ>0 be parameters. We define a *scaled graph* to be a graph $\textsc {Scale}(G, R, \epsilon , d)$ $:= (V, E, \hat{w})$, such that $\hat{w}(e) = \big \lceil \tfrac{w(e)d}{R \epsilon } \big \rceil$. Then for any path π in *G* such that π has at most *d* hops and weight $R \le w(\pi ) \le 2R$ we have: 1) $\hat{w}(\pi ) \le \big \lceil 2d/\epsilon \big \rceil$, and 2) $w(\pi ) \le \tfrac{\epsilon R}{d} \cdot \hat{w}(\pi ) \le (1+\epsilon ) w(\pi )$.

Hence given a hopset, we can ‘cover’ the entire graph by scaled graphs, by considering separate scaled graphs $G_i$ for the distance scale $[2^i, 2^{i+1}]$ for $i=1,\dots , \log (nW)$. In order to combine this with a hopset algorithm, we need to scale the graph after adding edges corresponding to a sequence of $2^i$-*restricted hopsets*, i.e. a graph that satisfies the hopset condition only for pairs within distance at most $2^i$. Slightly more formally, we define a scaled graph as follows: $G^j:= \textsc {Scale}\left( G \cup \bigcup _{r=0}^{j} H_{r}, 2^j, \epsilon , O(\beta )\right)$, where $H_{r}$ is a $2^r$-restricted hopset. The final hopsets of [20] are the union of all these restricted hopsetes, however for maintaining distances efficiently we need to run the algorithm in Lemma B.1 on all these scaled graphs simultaneously and take the minimum estimate over all the scales. Importantly, to handle edge insertions they use monotone ES trees which implies that the distances obtained from this algorithm is monotone. This is important for us in bounding the running time of our decremental data structures.

The following lemma shows that using the specific hopsets by [20], we can maintain approximate single-source distances.

**Lemma** 2.1*Let* G=(V,E,w) *be a decremental graph, and let* 0<ϵ<1 and 0<ρ<1/2 *be parameters, let* 0<p<1 *be a sampling probability, and let* A⊆V *be a set of sampled nodes, where each element of* *V* *is sampled uniformly at random with probability* *p*. *There exists an algorithm that maintains for each* v∈V: *(i) an approximate pivot* $\tilde{p}(v)\in A$; *(ii) an approximate bunch* $\tilde{B}(v)$ *; and (iii) distance estimates* $\delta _{\tilde{B}}(v,u)$ *for every* $u\in \tilde{B}(v)$. *These objects satisfy:*

- $$d_G(v,p(v)) \le \delta _{\tilde{B}}(v,\tilde{p}(v)) \le (1+\epsilon ) d_G(v,p(v))$$;
- $$ \{ w \in V: (1+\epsilon )d_G(v,w) < d_G(v, p(v)) \}\subseteq \tilde{B}(v) $$;
- $\delta _{\tilde{B}}$ *is monotone (non-decreasing over updates);*
- *for any* $u \in \tilde{B}(v)$: $d_G(u,v) \le \delta _{\tilde{B}}(u,v) \le (1+\epsilon ) d_G(u,v)$; *and*
- *with high probability,* $|\tilde{B}(v)| = O\left( \frac{\log n\log nW}{p}\right)$.

*The algorithm has total update time* $\tilde{O}(\tfrac{mn^\rho }{p}\cdot \log ^2(nW)) \cdot O(\frac{1}{\rho \epsilon })^{1+2/\rho }$.

### Proof sketch

First, using the algorithm of [20], we can maintain a $(\beta ,1+\epsilon )$-hopset *H* of size $\tilde{O}(m+n^{1+\rho })$ in update time $\tilde{O}(mn^{\rho } \beta )$ with hopbound $\beta =\tilde{O}(\frac{1}{\rho \epsilon })^{2\rho }$, which consists of a sequence of $2^i$-restricted hopsets $H_1,...,H_{\log nW}$. We then use the framework of [20] to maintain *approximate* clusters and hence approximate bunches on the scaled graphs. Since for all u,v∈V in G∪H there is a path with β-hops that with length at most $(1+\epsilon )d_G(u,v)$, they show that it is enough to maintain $O(\beta /\epsilon )$-hop bounded bunches/clusters on scaled graphs $G_1,...,G_{\log n W}$. We consider $(4\beta /\epsilon )$-*bounded bunches* $B_{G_i}(v)= \{ w: d_{G_i}(w,v) < d_{G_i}(v, p_i(v))\}$ where $p_i(v)$ is the closest sampled node in $G_i$ and $d_{G_i}(v, p_i(v)) \le 4\beta /\epsilon$. The scaling ensures that for each node *v* there exists $1 \le i \le \log nW$ such that $p_i(v)$ satisfies the property $d_{G_i}(v, p_i(v)) \le 4\beta /\epsilon$. Let *d* be the distances obtained by scaling back the estimates corresponding to $B_{G_i}(v) \cup p_i(v)$. There exists *i* such that $\tilde{p}(v) =p_i(v)$ satisfies $d_G(v,p(v)) \le d(v,\tilde{p}(v)) \le (1+\epsilon ) d_G(v,p(v))$. Also by choosing the appropriate error parameters (to account for the scaling and hopset errors) in Theorem 14 of [20] we can ensure that for any $u \in \tilde{B}(v)$ we have $d_G(u,v) \le d(u,v) \le (1+\epsilon ) d_G(u,v)$. Consider any $u \in \hat{B}(v)$. We have $d(u,v) \le (1+\epsilon )d_G(u,v) \le d_G(v,p(v)) \le d(v, \tilde{p}(v))$. Hence we can see that the bunch $B_{G_i}(v) \subseteq \hat{B}(v)$ and thus $\tilde{B}(v):=\bigcup _{1 \le i \le \log nW} B_{G_i}(v)$ contains all the nodes in $\hat{B}(v)$.

Finally since the algorithm of [20] (that is based on [4]) maintains bunches of size O(logn/p) on O(lognW) scaled graphs, we also have the bound on the size of the bunch. □

## Footnotes

1 In the introduction, we make two simplifying assumptions: (a) ϵ is a constant and (b) the ratio *W* between the maximum and minimum weight is polynomial in *n*. Unless otherwise noted, the cited algorithms have constant or polylogarithmic query time. Throughout we use $\tilde{O} (\cdot )$ notation to omit factors that are polylogarithmic in *n*.

2 Note that the algorithms of Roditty and Zwick [4] for unweighted, undirected graphs precede the more general algorithm of Bernstein [11] for weighted, directed graphs.

3 Although the picture implies *B*(*u*) and *B*(*v*) are disjoint, this also includes the case where they overlap, or even where u∈B(v) or v∈B(u).

4 The actual pivot only matters when a distance is queried, where we can use our explicitly maintained approximate pivots. See Sect. 4 for details.

5 We write each approximation with its own $\epsilon _i$. In the end, we set $\epsilon _i=\epsilon /3$ for all *i*, which gives us (2+ϵ)-APSP.

6 In the high-level overview *B*(*v*) has been used for the exact bunches. Since exact bunches do not occur in our actual algorithm, *B*(*v*) will denote these approximate bunches from here on, following conventions in prior work [1, 8, 88]

7 Recall that we denote $\tilde{w}$ for $(1+\epsilon _3)^{\lceil \log _{1+\epsilon _3}{w}\rceil }$.

8 If *w* becomes heavy we remove all the corresponding estimates.

9 In Sect. 4 and 5.1 we needed the distance between nodes *v* and their closest node in $A_1$, called the pivot. In this section, we only need an edge between *v* and an arbitrary neighbor $u\in A_1$.
